# Bitter taste signaling in tracheal epithelial brush cells elicits innate immune responses to bacterial infection

**DOI:** 10.1172/JCI150951

**Published:** 2022-07-01

**Authors:** Monika I. Hollenhorst, Rajender Nandigama, Saskia B. Evers, Igor Gamayun, Noran Abdel Wadood, Alaa Salah, Mario Pieper, Amanda Wyatt, Alexey Stukalov, Anna Gebhardt, Wiebke Nadolni, Wera Burow, Christian Herr, Christoph Beisswenger, Soumya Kusumakshi, Fabien Ectors, Tatjana I. Kichko, Lisa Hübner, Peter Reeh, Antje Munder, Sandra-Maria Wienhold, Martin Witzenrath, Robert Bals, Veit Flockerzi, Thomas Gudermann, Markus Bischoff, Peter Lipp, Susanna Zierler, Vladimir Chubanov, Andreas Pichlmair, Peter König, Ulrich Boehm, Gabriela Krasteva-Christ

**Affiliations:** 1 Institute of Anatomy and Cell Biology, Saarland University, School of Medicine, Homburg, Germany.; 2 Institute of Anatomy and Cell Biology, Julius-Maximilians-University, Würzburg, Germany.; 3 Institute for Experimental and Clinical Pharmacology and Toxicology, Saarland University, School of Medicine, Homburg, Germany.; 4 Institute of Anatomy, University of Lübeck, and Airway Research Center North (ARCN), Lübeck, Germany.; 5 German Center for Lung Research (DZL), Germany.; 6 Immunopathology of Virus Infections Laboratory, Institute of Virology, School of Medicine, Technical University of Munich, Munich, Germany.; 7 Walther-Straub-Institute for Pharmacology and Toxicology, Ludwig-Maximilians-University, Munich, Germany.; 8 Department of Internal Medicine V-Pulmonology, Allergology, Intensive Care Medicine, Saarland University Hospital, Homburg, Germany.; 9 FARAH Mammalian Transgenics Platform, Liège University, Liège, Belgium.; 10 Institute of Physiology and Pathophysiology, Friedrich-Alexander University Erlangen-Nürnberg, Erlangen, Germany.; 11 Clinic for Pediatric Pneumology, Allergology and Neonatology, Hannover Medical School, and Biomedical Research in Endstage and Obstructive Lung Disease Hannover (BREATH), Hannover, Germany.; 12 Department of Infectious Diseases and Respiratory Medicine and Division of Pulmonary Inflammation, Charité – University Medicine Berlin, corporate member of Free University Berlin and Humboldt-University Berlin, Berlin, Germany.; 13 Center for Molecular Signaling,; 14 Institute for Medical Microbiology and Hygiene, and; 15 Institute for Molecular Cell Biology and Research Center for Molecular Imaging and Screening, Saarland University, Homburg, Germany.; 16 Institute of Pharmacology, Johannes Kepler University Linz, Linz, Austria.; 17 Immunopathology of Virus Infections Laboratory, Institute of Virology, School of Medicine, Technical University of Munich, German Center for Infection Research (DZIF), Munich, Germany.

**Keywords:** Immunology, Pulmonology, Innate immunity

## Abstract

Constant exposure of the airways to inhaled pathogens requires efficient early immune responses protecting against infections. How bacteria on the epithelial surface are detected and first-line protective mechanisms are initiated are not well understood. We have recently shown that tracheal brush cells (BCs) express functional taste receptors. Here we report that bitter taste signaling in murine BCs induces neurogenic inflammation. We demonstrate that BC signaling stimulates adjacent sensory nerve endings in the trachea to release the neuropeptides CGRP and substance P that mediate plasma extravasation, neutrophil recruitment, and diapedesis. Moreover, we show that bitter tasting quorum-sensing molecules from *Pseudomonas aeruginosa* activate tracheal BCs. BC signaling depends on the key taste transduction gene *Trpm5*, triggers secretion of immune mediators, among them the most abundant member of the complement system, and is needed to combat *P*. *aeruginosa* infections. Our data provide functional insight into first-line defense mechanisms against bacterial infections of the lung.

## Graphical abstract

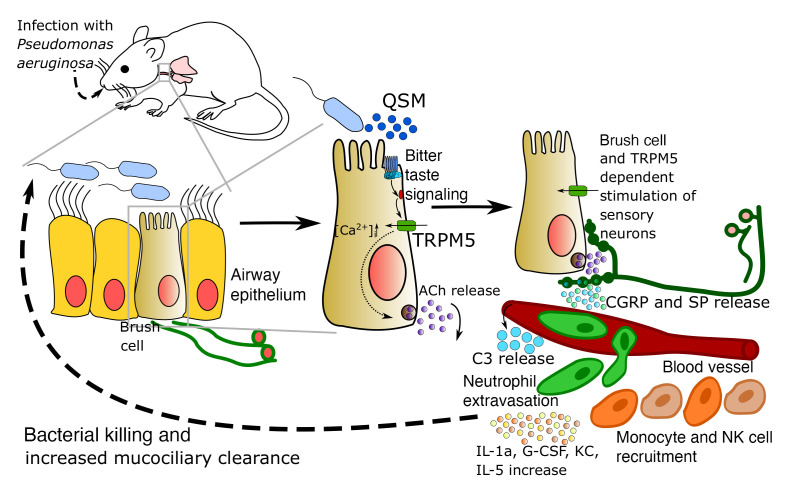

## Introduction

Constant exposure of the airways to inhaled particles and pathogens requires efficient early immune responses protecting against infections. These processes are largely mediated by the airway epithelium that is equipped with a broad spectrum of mechanisms to detect and eliminate inhaled pathogens. During recent years, airway epithelial chemosensory cells such as solitary chemosensory cells (SCCs) in the nose and brush cells (BCs) in the trachea have been proposed as possible candidates to detect pathogens and initiate protective innate immune responses. While both SCCs and BCs express members of the canonical bitter signaling cascade, including taste receptors, the G-protein α-gustducin (GNAT3), phospholipase C β2 (PLCβ2), and the transient receptor potential melastatin 5 channel (TRPM5) (1–3) that is permeable to monovalent cations (4), these 2 chemosensory cell types are differentially innervated by either trigeminal (SCCs) or vagal (BCs) sensory nerve fibers (1, 2, 5). Recently, we and others have shown that tracheal BCs express functional bitter taste receptors (Tas2Rs) (2, 6–9). BCs respond to stimulation with the bitter substance denatonium and quorum-sensing molecules (QSMs) with a release of acetylcholine (ACh), acting in a paracrine manner on neighboring ciliated epithelial cells and nerve fibers, enhancing mucociliary clearance in the lower airways via muscarinic ACh receptors (mAChRs) (2, 10) and eliciting protective breathing reflexes via nicotinic AChRs (nAChRs) (11).

A subset of the cholinoceptive nerve fibers associated with BCs contains the neuropeptide calcitonin gene–related peptide (CGRP) (1), which is often stored together with the proinflammatory neuropeptide substance P (SP) (12). Both peptides play a major role in protective inflammatory processes known as neurogenic inflammation, characterized by vasodilation, plasma extravasation, and mast cell recruitment (5, 13). Activation of the nerve fibers followed by CGRP and SP release is mediated by direct activation of the ion channels TRPV1 and TRPA1 by capsaicin (colon, airways), bacterial lipopolysaccharides (intestine), or epithelial shedding (asthmatic airways) (14–17).

Sensory nerve fibers are not in direct contact with the luminal content in an intact airway epithelium (18, 19). How bacteria on the epithelial surface are detected and first-line protective mechanisms are initiated to combat bacterial infections are not known. Pathogens or bacterial molecules are in contact with sensory nerve endings only in pathophysiological situations upon damage of the epithelium or in severe infections. Interestingly, nicotine and ACh are able to induce the release of CGRP from rat trachea (20), raising the possibility that ACh released from BCs after pathogen detection might be involved in the induction of neurogenic inflammation. In the nose, activation of SCCs with denatonium is able to induce plasma extravasation and mast cell degranulation that depend on the presence of intact trigeminal innervation of the nose (5). However, it remains unknown whether this response is linked to increased survival of infected animals and whether it occurs in the lower airways.

Here, we tested the hypothesis that BCs are epithelial sensors for pollution with *Pseudomonas aeruginosa* and *Streptococcus pneumoniae*, bacteria challenging the respiratory tract that can both be responsible for nosocomial infections. Specifically, we asked whether BC activation with the bitter substance denatonium or with bacterial Tas2R agonists could induce neurogenic inflammation and thus be a first-line defense against bacterial infections.

## Results

### Bitter taste signaling in BCs induces plasma leakage.

**Figure 1:**
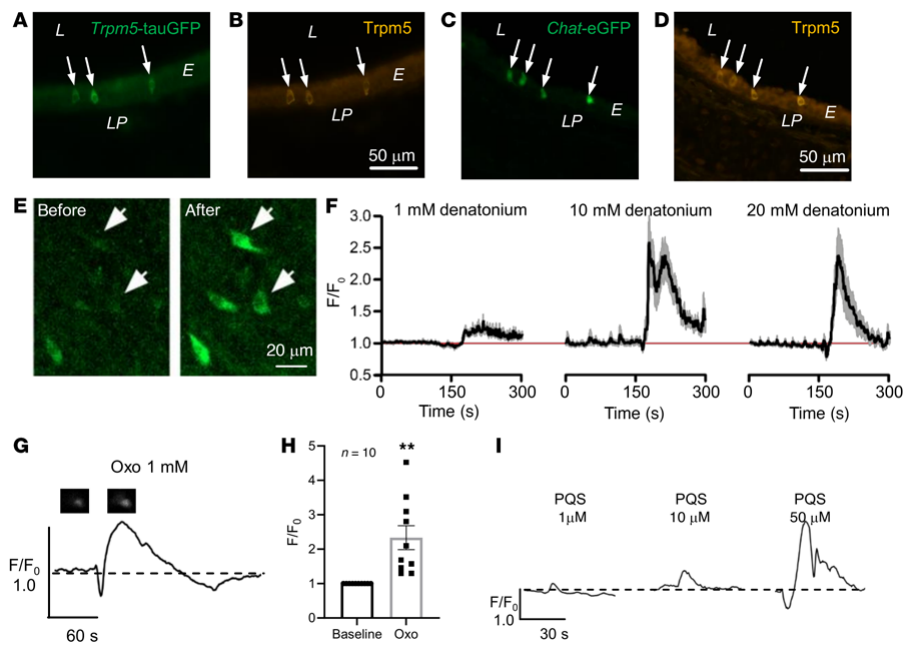
Measurements of intracellular calcium levels in *Trpm5*-GCaMP3 cells. (**A** and **B**) Immunohistochemical staining of tracheal sections from a *Trpm5*-tauGFP (**A** and **B**) or *Chat*-eGFP (**C** and **D**) mouse using an antiserum against Trpm5. L, lumen; E, epithelium; LP, lamina propria. (**E**) Image of the trachea of a *Trpm5*-GCaMP3 mouse showing an increase in GCaMP3 fluorescence in individual Trpm5^+^ cells after denatonium application (20 mM). Scale bars: 50 μm (**A**–**D**) and 20 μm (**E**). (**F**) Graphs showing Ca^2+^ transients of Trpm5^+^ tracheal cells from *Trpm5*-GCaMP3 mice responding to 1, 10, or 20 mM denatonium applied at 180 seconds (left, central, and right graph, respectively). Data shown as mean ± SEM of the normalized (F/F_0_) GCaMP3 fluorescence intensities. Red line shows the initial (start recording, *t* = 0, F/F_0_ = 1) level of the normalized intensities. *n* cells = 23 from 3 mice. (**G**) Representative curve for the Ca^2+^ response of a Trpm5^+^ cell stimulated with 1 mM *N*-(3-oxododecanoyl)-L-homoserine lactone (Oxo). Images of the Trpm5^+^ cell show GCaMP3 baseline fluorescence and fluorescence after stimulation. (**H**) Oxo (1 mM) led to a significant transient increase in [Ca^2+^]_i_ in Trpm5^+^ cells from *Trpm5*-GCaMP3 mouse tracheae. Data shown as single values and mean ± SEM of the normalized (F/F_0_) GCaMP3 fluorescence intensities (*n* cells = 10 from 3 mice). **P < 0.01 by 2-tailed, unpaired Student’s *t* test. (**I**) Representative curve for the Ca^2+^ response of Trpm5^+^ cells to 1, 10, or 50 μM *Pseudomonas aeruginosa* quinolone signal (PQS) (1 μM: *n* cells = 28 from 3 tracheal pieces; 10 μM: *n* cells = 30 from 3 tracheal pieces; 50 μM: *n* cells = 40 from 4 tracheal pieces).

We first confirmed Trpm5 expression exclusively in BCs using an antiserum against Trpm5 in tracheal sections of *Trpm5*-tauGFP and *Chat*-eGFP mice (Figure 1, A–D). The *Trpm5*-tauGFP mice express tauGFP from the Rosa26 locus following Cre-mediated activation only in *Trpm5*-expressing cells. *Chat*-eGFP mice (21) express GFP under the control of the choline acetyltransferase (*Chat*) promoter exclusively in BCs in the tracheal epithelium (6). Trpm5 expression was detected only in ChAT^+^ BCs in the trachea. To be able to analyze BC activation, we then generated mice expressing an optogenetic Ca^2+^ indicator specifically in these cells (*Trpm5*-GCaMP3). Stimulation with denatonium, a Tas2R agonist (22, 23), increased intracellular Ca^2+^ ([Ca^2+^]_i_) levels in GCaMP3^+^ cells (Trpm5^+^ BCs) in freshly explanted intact tracheae and this response was progressively stronger with increasing denatonium concentrations from 1 mM to 20 mM (Figure 1, E and F). The effect of denatonium on BCs was dependent on G-protein signaling, as it was abolished in the presence of the G_βγ_-subunit inhibitor gallein (Supplemental Figure 1A; supplemental material available online with this article; https://doi.org/10.1172/JCI150951DS1). Intriguingly, application of the *P*. *aeruginosa* quorum-sensing molecules (QSMs) *N*-(3-oxododecanoyl)-L-homoserine lactone (Oxo-HSL) and the *Pseudomonas* quinolone signal (PQS), belonging to the *las* and *pqs* quorum-sensing system, respectively, significantly increased [Ca^2+^]_i_ levels in Trpm5^+^ BCs in intact tracheae (Figure 1, G–I). The response to PQS was also concentration dependent (1, 10, and 50 μM; Figure 1I). Oxo-HSL and supernatants of the cystic fibrosis *P*. *aeruginosa* isolate NH57388A (24) act on Tas2R105 and Tas2R108, which are the most commonly expressed Tas2Rs in tracheal BCs (Supplemental Figure 1, B and C, and refs. 6, 7).

**Figure 2:**
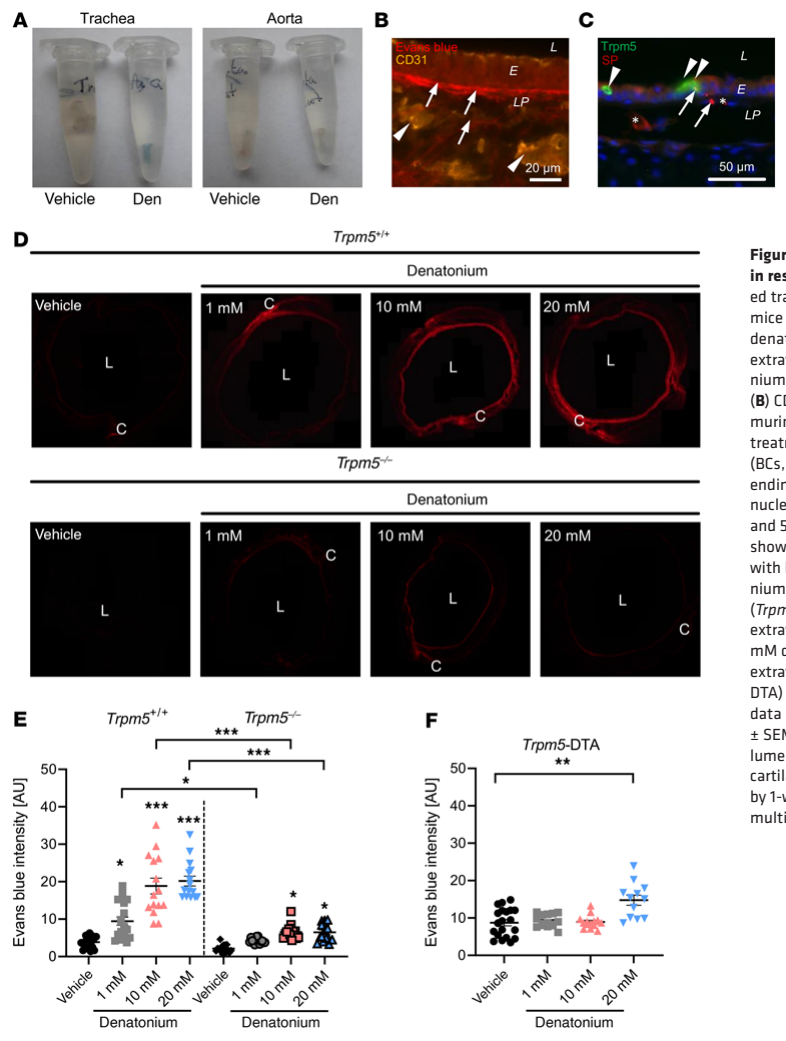
Evans blue (EB) extravasation in response to denatonium. (**A**) Explanted tracheae (left) and aortae (right) from mice treated with vehicle control (PBS) or denatonium (den) show blue color due to EB extravasation in the trachea after denatonium treatment. EB is absent in the aorta. (**B**) CD31 staining with EB fluorescence in murine tracheal sections after denatonium treatment. (**C**) Costaining against Trpm5 (BCs, arrowheads) and SP (sensory nerve endings) showing blood vessels (*) and nuclei (blue, DAPI). Scale bars: 20 μm (**B**) and 50 μm (**C**). (**D**) Images of tracheal rings showing EB fluorescence of animals treated with PBS (vehicle), 1, 10, or 20 mM denatonium in WT (*Trpm5^+/+^*) or *Trpm5*-knockout (*Trpm5^–/–^*) mice. (**E**) Quantification of EB extravasation in response to 1, 10, or 20 mM denatonium. (**F**) Quantification of EB extravasation in BC-depleted mice (*Trpm5*-DTA) in response to denatonium. In **E** and **F**, data are shown as single values and mean ± SEM (*n =* 12–20 rings from 3–4 mice). L, lumen; E, epithelium; LP, lamina propria; C, cartilage. *P < 0.05; **P < 0.01; ***P < 0.001 by 1-way ANOVA followed by Bonferroni’s multiple-comparison correction.

To study plasma leakage from blood vessels, a hallmark of neurogenic inflammation, we next evaluated Evans blue (EB) extravasation in response to denatonium application (Figure 2). Thirty minutes after inhalation of a single dose of denatonium (4 μL) into the trachea, we observed EB staining restricted to the trachea (Figure 2A), thus excluding further systemic effects on other organs (Figure 2A). In order to define the precise localization of the dye as well as to quantify it in situ, we next established an approach for the evaluation of EB extravasation based on the quantification of its emission of red fluorescence in tissue sections (Supplemental Figures 2 and 3A). The dye was observed outside of blood vessels labeled for CD31 and bound to collagen fibers in the basal membrane and in the lamina propria (Figure 2B). These CD31^+^ blood vessels were innervated by peptidergic nerve fibers (Figure 2C). Denatonium application (1, 10, or 20 mM) induced a dose-dependent increase in EB extravasation (Figure 2, D and E) in wild-type (WT) control mice. In contrast, the extravasation induced by 1 mM denatonium was completely abolished in *Trpm5*-knockout (*Trpm5^–/–^*) mice, indicating that the EB extravasation was mediated by BCs and required activation of the Trpm5 channel. Consistent with this, responses to 10 and 20 mM denatonium stimulation were highly reduced in *Trpm5^–/–^* mice when compared with controls (Figure 2, D and E). To demonstrate that the observed plasma extravasation in response to denatonium was due to BC activation, we generated *Trpm5*-DTA mice, in which BCs were ablated by the expression of diphtheria toxin A (DTA), a protein component of DT that is produced exclusively in these *Trpm5*-expressing cells. Indeed, we did not observe any staining for BC-specific marker proteins in tracheal sections from *Trpm5*-DTA mice (Supplemental Figure 4). More importantly, EB extravasation was absent in response to 1 or 10 mM denatonium in *Trpm5*-DTA mice (Figure 2F). However, application of 20 mM denatonium still led to a moderate increase in EB extravasation in *Trpm5*-DTA mice (Figure 2F). Since free nerve endings themselves are chemosensitive, we addressed the possibility that BC-independent extravasation resulted from a direct neuronal activation. Employing *Trpm5*-tauGFP mice and specific antibodies against Trpm5, we confirmed publicly available single-cell RNA sequencing data (www.imgen.org) demonstrating the absence of Trpm5 in the mouse jugular-nodose-complex (JNC) and dorsal root ganglia (DRG) neurons (Supplemental Figure 5, A and B). Supportively, we did not observe any changes in [Ca^2+^]_i_ in response to 1 mM denatonium in sensory neurons responding to cinnamaldehyde and capsaicin (Supplemental Figure 5C). Indeed, we detected expression of *Tas2r108* and *Tas2r119*, but not *Trpm5*, in DRG neurons (Supplemental Figure 5D), and a small population of neurons showed an increase in [Ca^2+^]_i_ in response to 20 mM denatonium (Supplemental Figure 5, E and F). This indicates that the residual neurogenic inflammation observed with 20 mM denatonium in mice lacking BCs is induced by direct neuronal stimulation, while the effects at lower doses can be specifically attributed to Trpm5 activation in BCs.

### Denatonium induces neutrophil recruitment and diapedesis through blood vessels.

**Figure 3:**
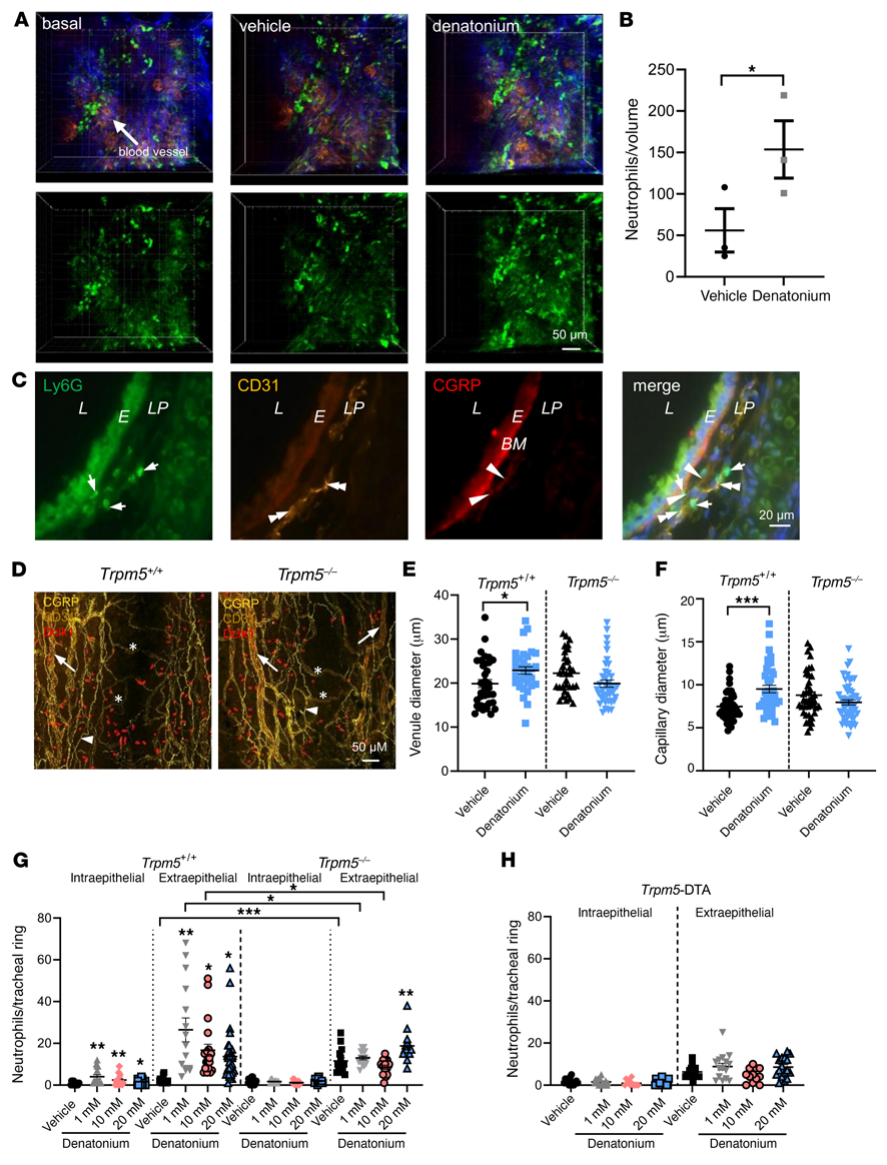
Denatonium evokes neutrophil recruitment and blood vessel dilation in the trachea. (**A** and **B**) In vivo 2-photon microscopy of neutrophils and blood vessels in the trachea of *Ly6G*-GFP mice. (**A**) Denatonium-increased neutrophil (green) extravasation from blood vessels (red) (blue: second harmonic generation signal, collagen fibers) in WT mice compared to basal and vehicle-treated (HEPES-treated) controls. (**B**) Evaluation of the results in **A** (*n =* 3 mice). Volume: 300 × 300 × 60 μm. Denatonium: 20 mM. (**C**) Tracheae stained for Ly6G, CD31, and CGRP showed neutrophil recruitment (Ly6G, green) in proximity to blood vessels (CD31, yellow) and CGRP^+^ nerve endings (red) at the same site (merge). Evans blue bound to the basal membrane (BM, bright red). L, lumen; E, epithelium; LP, lamina propria. Merge: nuclei stained for DAPI (blue). (**D**) Whole-mount staining of tracheae from *Trpm5^+/+^* and *Trpm5^–/–^* mice. BC: Dclk1 (red); blood vessels: CD31 (yellow), venules = arrows, capillaries = stars; nerves: CGRP (yellow, arrowheads). Scale bars: 50 μm (**A** and **D**) and 20 μm (**C**). (**E** and **F**) Quantification of the diameter of venules (**E**) and capillaries (**F**) (*n =* 32–53 vessels from 4 mice). (**G** and **H**) Analysis of neutrophils per tracheal ring in WT (*Trpm5^+/+^*) mice, *Trpm5^–/–^* mice, and BC-deficient *Trpm5*-DTA mice (*n =* 12–25 rings from 4–5 mice). In **B** and **E**–**H**, data are shown as single values and mean ± SEM. *P < 0.05; **P < 0.01; ***P < 0.001 by 2-tailed, unpaired Student’s *t* test (**B**) or 1-way ANOVA followed by Bonferroni’s multiple-comparison correction (**E**–**H**).

To investigate whether neutrophils are recruited to the site of plasma extravasation upon BC stimulation, we imaged blood vessels in the lamina propria of the trachea in intubated and ventilated mice using in vivo 2-photon microscopy before and 30 minutes after a single inhalation of either denatonium or vehicle. We observed neutrophil recruitment 30 minutes after denatonium inhalation but not in the vehicle-treated control group (Supplemental Videos 1 and 2). Moreover, 3D reconstructions of the image stacks revealed a higher number of neutrophils in the tissue surrounding the blood vessels in the denatonium-treated animals, suggestive of an extravasation of neutrophils (Figure 3, A and B).

In order to determine the exact site of neutrophil recruitment and diapedesis, we performed immunohistochemical analyses on serial tracheal sections using markers for neutrophils (Ly6G), endothelial cells (CD31), and peptidergic sensory nerve fibers (CGRP). We found that CGRP^+^ nerve endings projected to small blood vessels in the lamina propria that contained neutrophils (Figure 3C). Neutrophils were found predominantly in the immediate vicinity of the blood vessels. Vasodilation, another feature of neurogenic inflammation, was observed in venules and capillaries after stimulation with 1 mM denatonium in WT, but not in *Trpm5^–/–^* mice (Figure 3, D–F).

Denatonium-treated WT mice exhibited increased numbers of neutrophils in the intra- as well as extraepithelial layers when compared with vehicle-treated controls (Figure 3G). The increase in neutrophil numbers already plateaued at a denatonium concentration of 1 mM. In contrast, the denatonium-induced increase in neutrophil extravasation at 1 and 10 mM was completely abolished in *Trpm5^–/–^* mice and was significantly reduced compared with WT mice (Figure 3G). Consistent with this, denatonium also failed to evoke changes in neutrophil numbers in the tracheae of *Trpm5*-DTA mice lacking BCs (Figure 3H). Extraepithelial neutrophil numbers were higher in vehicle-treated *Trpm5^–/–^* mice in comparison with WT controls (Figure 3G). Taken together, these data demonstrate that the activation of Trpm5-dependent signal transduction in tracheal BCs induces neutrophil recruitment in the trachea.

### The denatonium-induced plasma extravasation and neutrophil recruitment are dependent on cholinergic transmission to sensory neurons.

**Figure 4:**
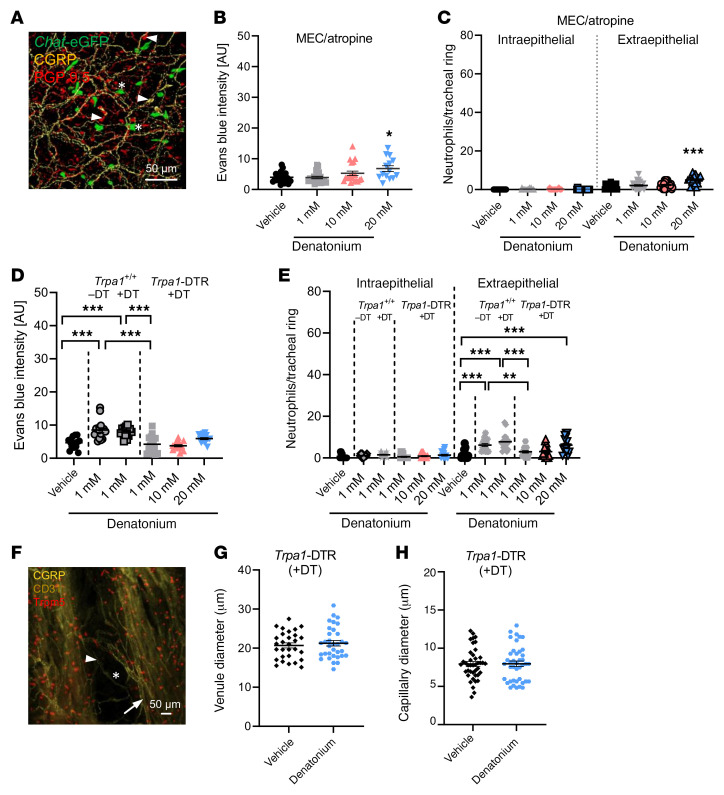
The denatonium-induced neurogenic inflammation is mediated via cholinergic signaling and sensory nerve activation. (**A**) Nerve fibers (PGP9.5^+^: red) containing the neuropeptide CGRP (yellow) in *Chat*-eGFP mice (ChAT^+^ cells: green, stars). Arrowheads: neuroendocrine cells labelled for PGP9.5 and/or CGRP. Scale bar: 50 μm. (**B**) Quantification of Evans blue (EB) extravasation in response to 1, 10, or 20 mM denatonium in WT mice treated with the AChR antagonists mecamylamine (MEC) and atropine. (**C**) Analysis of neutrophil numbers per tracheal ring in WT mice treated with MEC and atropine. (**D**) Quantification of EB extravasation in response to 1, 10, or 20 mM denatonium in *Trpa1*-DTR mice treated with diphtheria toxin (DT) and in response to 1 mM denatonium in naive WT or DT-treated WT (*Trpa1^+/+^*) mice. (**E**) Analysis of neutrophil number per tracheal ring in response to 1, 10, or 20 mM denatonium in *Trpa1*-DTR mice treated with DT. Naive or DT-treated WT (*Trpa1^+/+^*) mice stimulated with 1 mM denatonium served as controls. (**F**) Staining of venules (arrows) and capillaries (stars) in tracheae from DT-treated *Trpa1*-DTR mice. BC: Trpm5 (red), blood vessels: CD31 (yellow), nerves: CGRP (yellow, arrowheads). Scale bar: 50 μm. (**G** and **H**) Quantification of the venule (**G**) and capillary diameter (**H**). *n =* 29–41 vessels from 4 mice. In **B**–**E**, **G**, and **H**, data are shown as single values and mean ± SEM (*n =* 14–24 rings from 3–4 mice). *P < 0.05; **P < 0.01; ***P < 0.001 by 1-way ANOVA followed by Bonferroni’s multiple-comparison correction (**B**–**E**) or 2-tailed, unpaired Student’s *t* test (**G** and **H**).

We observed a dense nerve fiber network labeled for the pan-neuronal marker PGP9.5 and for CGRP in whole-mount trachea preparations (Figure 4A and Supplemental Figure 6A). ACh-producing tracheal BCs (*Chat*-eGFP^+^) appeared to be in close proximity to CGRP^+^ nerve fibers (Figure 4A). We also detected PGP9.5^+^ and CGRP^+^ tracheal epithelial cells that likely represent neuroendocrine cells (Figure 4A and Supplemental Figure 6B). Previously, we have shown that inhalation of bitter substances induced breathing reflexes, being sensitive to nAChR blockade and epithelium removal. To explore whether cholinergic transmission of BC-released ACh on BC-approaching nerve fibers is responsible for the observed denatonium-induced effects, we treated WT mice with the nAChR antagonist mecamylamine and the mAChR antagonist atropine prior to tracheal inhalation of denatonium. Cholinergic inhibition abolished EB extravasation and recruitment of extraepithelial or intraepithelial neutrophils in response to 1 and 10 mM denatonium (Figure 4, B and C), while moderate effects were still detected at 20 mM denatonium (Figure 4, B and C). To further elucidate the role of peptidergic sensory neurons in the BC-dependent neurogenic inflammation, we generated *Trpa1*-DTR mice, in which *Trpa1*-expressing neurons were specifically ablated upon DT administration (Supplemental Figure 7, A–K). Intriguingly, in these mice deficient for sensory innervation (Trpa1 expression in sensory neurons largely overlaps with Trpv1; Supplemental Methods and Supplemental Figure 7) we did not observe an EB extravasation and a recruitment of extraepithelial or intraepithelial neutrophils in response to intratracheal administration of 1 or 10 mM denatonium compared with naive WT mice and DT-treated WT mice (Figure 4, D and E). A moderate effect was observed at 20 mM stimulation that can most likely be explained by a population of Trpv1^+^ neurons that remained after the depletion of Trpa1^+^ neurons (Supplemental Figure 7, I–K). In support of this, we did not observe changes in blood vessel diameter in mice depleted of Trpa1^+^ sensory neurons (DT-treated *Trpa1*-DTR) (Figure 4, F–H). This further emphasizes the interaction between BCs and sensory nerves as a prerequisite for the neurogenic vasodilatation in the airways.

### Denatonium-induced plasma extravasation and neutrophil recruitment is dependent on CGRP and SP release.

**Figure 5:**
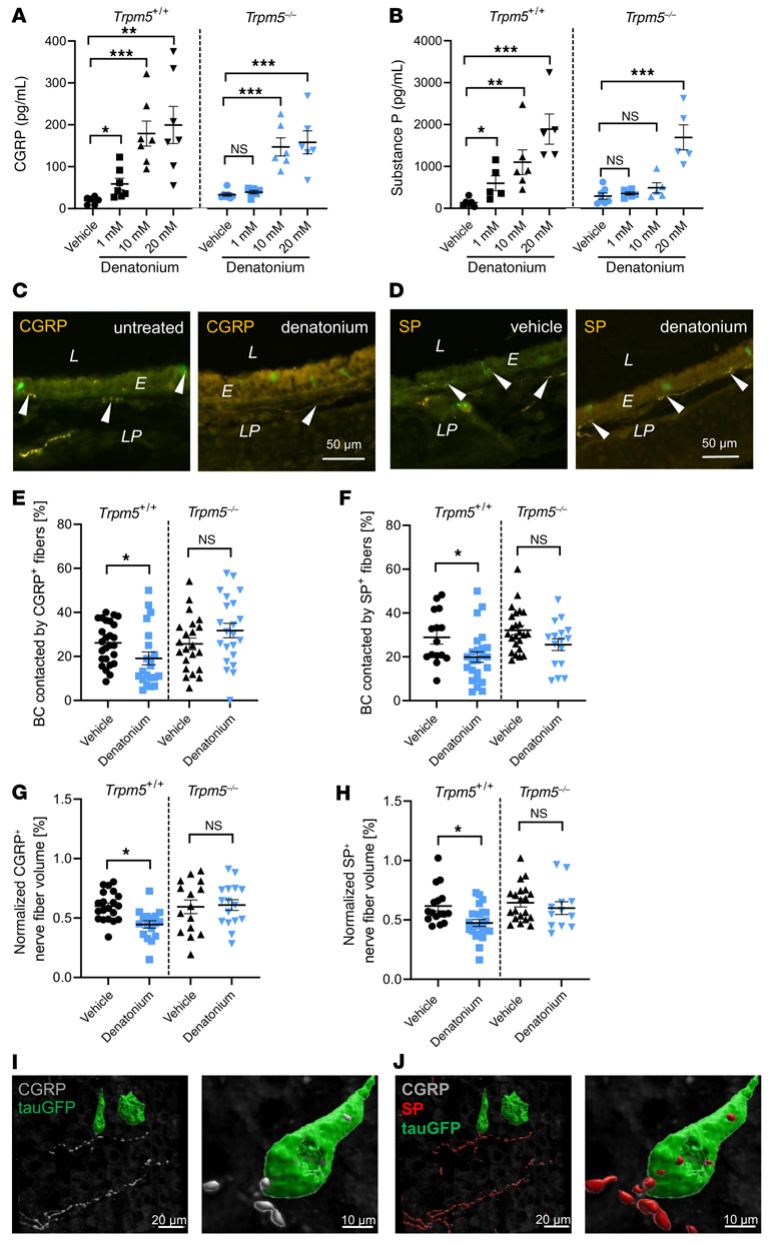
CGRP and SP trigger tracheal neurogenic inflammation in response to denatonium. (**A** and **B**) CGRP and SP measurements in excised tracheae with and without denatonium stimulation in WT (*Trpm5^+/+^*) and *Trpm5^–/–^* mice (*n =* 5–7 samples from 6–10 mice). (**C** and **D**) Staining of CGRP^+^ (**C**) and SP^+^ (**D**) nerve fibers (arrowheads) in tracheal slices of control (vehicle) and denatonium-treated WT mice. L, lumen; E, epithelium; LP, lamina propria. Scale bars: 50 μm (**C** and **D**). (**E** and **F**) Quantification of contacts between BCs and CGRP^+^ (**E**) and SP^+^ (**F**) nerve endings in whole-mount preparations of tracheae treated with vehicle or 1 mM denatonium (*n* = 15–29 image stacks containing 16–77 contacts/sample from 8 mice). (**G** and **H**) Treatment of tracheae with 1 mM denatonium significantly reduced the CGRP^+^ (**G**) and the SP^+^ (**H**) nerve fiber volume (normalized to the total stack volume) in *Trpm5^+/+^* but not in *Trpm5^–/–^* mice (*n =* 12–23 volumes from 4 mice). (**I**) 3D reconstruction of a whole-mount tracheal preparation immunofluorescence with Imaris software (see Supplemental Methods). BCs (GFP, green) are approached by CGRP^+^ nerves. (**J**) Nerve endings approaching BCs are CGRP^+^ and SP^+^. Scale bars: 20 μm (left images in **I** and **J**) and 10 μm (right images). In **A**, **B**, and **E**–**H**, data are shown as single values ± SEM. *P < 0.05, **P < 0.01, ***P < 0.001 by 1-way ANOVA followed by Bonferroni’s multiple-comparison correction (**A** and **B**) or 2-tailed, unpaired Student’s *t* test (**E**–**H**).

To test whether BC activation induces neuropeptide release that results in neurogenic inflammation, we next studied the direct release of CGRP and SP from the mouse trachea. Denatonium stimulation of explanted tracheae resulted in a robust increase in CGRP and SP release in the trachea already after 5 minutes (Figure 5, A and B). The release of CGRP and SP in response to 1 mM denatonium as well as of SP to 10 mM denatonium were completely abolished in *Trpm5^–/–^* mice. At 20 mM denatonium, the increase in the concentration of both peptides was only slightly reduced in *Trpm5^–/–^* mice. Since the number of CGRP^+^ epithelial cells was not altered after denatonium inhalation (1, 10, or 20 mM; Supplemental Figure 8A), we hypothesized that the peptides had been released from nerve endings upon activation by BCs. SP^+^ and CGRP^+^ nerve fibers were abundant in naive WT and *Trpm5^–/–^* mice (Figure 5, C and D, and Supplemental Figure 8, B–E). We found in tracheal whole-mount preparations that about a quarter of BCs exhibit contacts to SP^+^ and CGRP^+^ nerve fibers (Figure 5, E and F). After denatonium inhalation, the amount of intraepithelial CGRP^+^ and SP^+^ nerve fibers was largely reduced in WT mice (Figure 5, C and D). The number of BCs contacted by CGRP^+^ as well as by SP^+^ fibers decreased 30 minutes after stimulation with 1 mM denatonium by 30% in WT, but not in *Trpm5^–/–^* mice. Contacts with PGP9.5^+^ fibers remained unchanged (Figure 5, E and F, and Supplemental Figure 8, F–H), thus pointing toward a BC-mediated depletion of CGRP and SP stores in the sensory nerve endings. Notably, stimulation with 1 mM denatonium also reduced the CGRP^+^ as well as SP^+^ nerve fiber volume in the lamina propria in WT, but not *Trpm5^–/–^* mice (Figure 5, G and H). Double immunostaining for SP and CGRP on tracheal sections and whole mounts revealed the presence of both neuropeptides in the very same nerve fibers and varicosities that were approaching BCs (Figure 5, I and J, and Supplemental Figure 8I). Given the high overlap between CGRP and SP observed in the neurons in the sensory ganglia and in the peripheral sensory nerve fibers (Supplemental Figure 9), and the Trpm5 dependency of the release of both peptides to 1 mM denatonium, a simultaneous corelease of both neuropeptides is anticipated.

**Figure 6:**
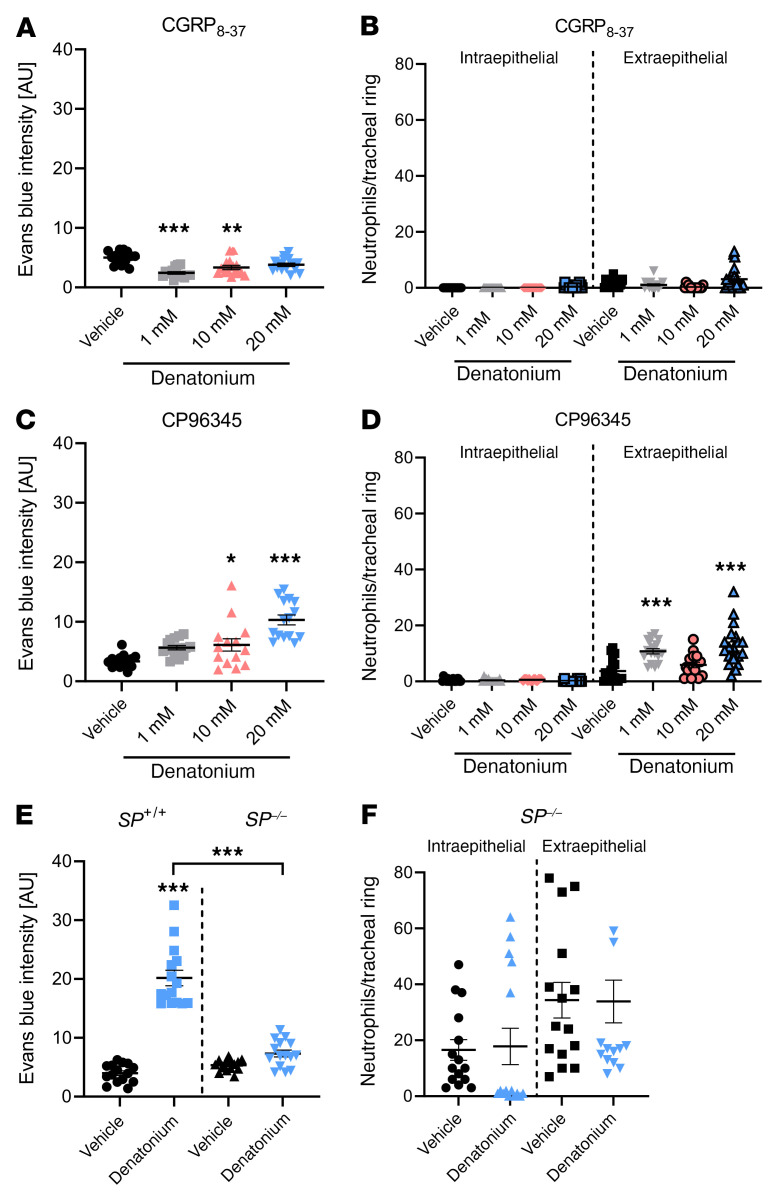
Denatonium-induced neurogenic inflammation is CGRP and SP dependent. (**A**) Quantification of Evans blue (EB) intensity in response to 1, 10, or 20 mM denatonium in *Trpm5^+/+^* mice after treatment with the CGRP receptor antagonist CGRP_8–37_. (**B**) Analysis of neutrophils per tracheal ring in *Trpm5^+/+^* mice treated with CGRP_8–37_. (**C**) Evaluation of EB intensity in response to 1, 10, or 20 mM denatonium in *Trpm5^+/+^* mice treated with the NK1-R inhibitor CP96345. (**D**) Analysis of neutrophils per tracheal ring in *Trpm5^+/+^* mice treated with CP96345. (**E**) Evaluation of EB intensity in *SP*^–/–^ and WT mice (*SP*^+/+^) stimulated with vehicle or denatonium (20 mM) revealed reduced EB extravasation in *SP*^–/–^ mice in response to denatonium. (**F**) Analysis of neutrophils per tracheal ring in *SP*^–/–^ mice. In **A**–**F**, *n =* 14–20 tracheal rings of 3–4 mice. Data are shown as single values ± SEM. *P < 0.05, **P < 0.01, ***P < 0.001 by 1-way ANOVA followed by Bonferroni’s multiple-comparison correction.

Next, we investigated the role of CGRP and SP in denatonium-induced neurogenic inflammation. When CGRP receptors were inhibited with the peptide CGRP_8–37_ (800 ng), administered 24 hours and 2 hours before stimulation with denatonium (25), the denatonium-induced increase in EB extravasation and intra- or extraepithelial neutrophil recruitment in WT mice were completely abolished (Figure 6, A and B). Inhibition of the SP receptor neurokinin-1 receptor (NK1-R) with CP96345 (2.5 μg/g bodyweight), injected i.p. 30 minutes before denatonium application, antagonized the EB extravasation after stimulation with 1 mM denatonium (Figure 6C). Responses to 10 and 20 mM denatonium were significantly reduced compared with WT mice and in the range of those observed in *Trpm5^–/–^* mice (10 mM) (Supplemental Figure 10A). In contrast to previous observations following denatonium stimulation (Figure 6D), extraepithelial neutrophil numbers were significantly reduced after CP96345 treatment compared with stimulation with 1 mM (*P =* 0.0003) and 10 mM denatonium (*P =* 0.01) without inhibitor (Supplemental Figure 10B) and comparable with the responses in *Trpm5^–/–^* mice. However, in conditional SP-deficient (*SP^–/–^*) mice, EB extravasation as well as the number of extra- and intraepithelial neutrophils did not increase in response to denatonium inhalation even at 20 mM (Figure 6, E and F). The lack of increase in EB extravasation in *SP^–/–^* mice, i.e., an absence of SP and neurokinin A (NKA) peptide release, and the moderate residual response in CP96345-treated mice (NK1-R inhibition) suggest a possible role of NK2-R stimulation with SP and NKA at high concentrations of the released peptides.

Immunostaining for CD31 in *SP^–/–^* mice showed that blood vessels were dilated in denatonium-treated mice, indicating that CGRP mediates the vessel dilation (Supplemental Figure 11, A and B). Together, all findings indicate that SP and CGRP are coreleased in the trachea upon BC stimulation, and both are essential for mediating BC-induced plasma extravasation and neutrophil recruitment, whereas CGRP mediates the onset and SP the potentiation of the effects.

Since CGRP and SP impact degranulation of mast cells, which secondarily recruit other immune cells, we investigated the denatonium-induced recruitment and degranulation of mast cells in the trachea. Interestingly, we did not observe mast cells in the lamina propria or the epithelium of paries cartilagineus either in vehicle-treated WT mice or after stimulation with 1 or 20 mM denatonium (Supplemental Figure 12). Mast cells were mainly localized in the paries membranaceus trachealis and their number and degranulation increased significantly only in response to 20 mM denatonium and not to 1 mM, which suggests that BC-induced effects do not involve mast cells.

### Bacterial substances induce neurogenic inflammation dependent on Trpm5 signaling.

**Figure 7:**
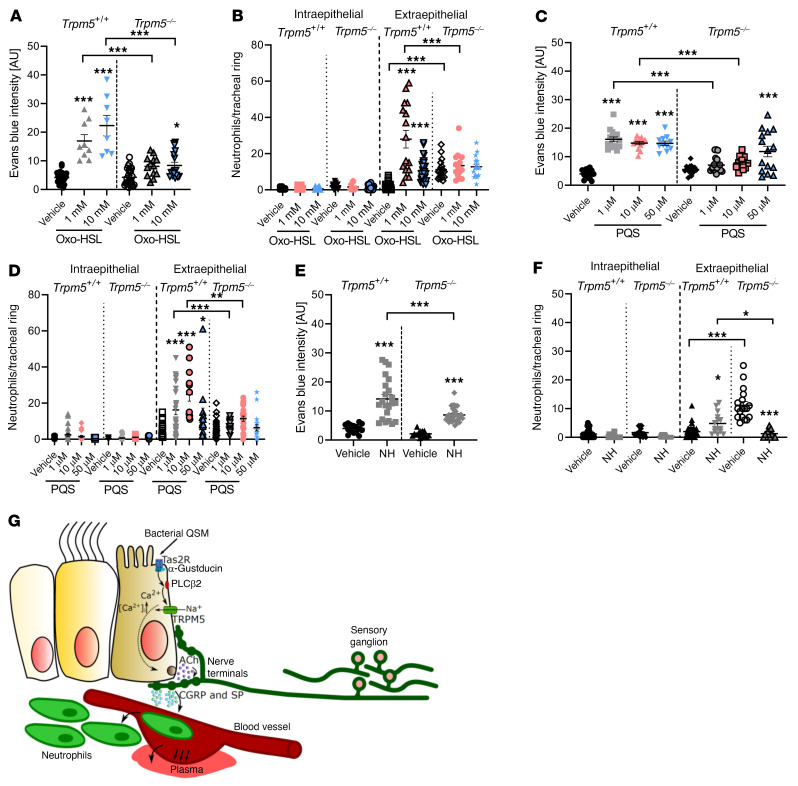
Evans blue (EB) extravasation and neutrophil recruitment in response to bacterial QSMs or bacterial culture supernatants. (**A**, **C**, and **E**) Quantification of EB intensity in response to the QSMs *N*-(3-oxododecanoyl)-L-homoserine lactone (Oxo-HSL) and *Pseudomonas* *aeruginosa* quinolone signal (PQS) as well as supernatants of the *P*. *aeruginosa* strain NH57388A (NH) in WT (*Trpm5^+/+^*) and *Trpm5*-deficient (*Trpm5*^–/–^) mice. (**B**, **D** and **F**) Analysis of intra- and extraepithelial neutrophils per tracheal ring in WT (*Trpm5^+/+^*) and *Trpm5*^–/–^ mice. In **A**–**F**, data are shown as single values ± SEM (*n =* 8–30 rings from 3–5 mice). *P < 0.05; **P < 0.01; ***P < 0.001 by 1-way ANOVA followed by Bonferroni’s multiple-comparison correction. (**G**) Proposed mechanism of induction of neurogenic inflammation after stimulation of the bitter signaling cascade in tracheal epithelial BCs with bitter or bacterial substances. Substances bind to bitter taste receptors, which activates α-gustducin, leading to Ca^2+^ release from intracellular stores that activates Trpm5 and ACh release from BCs. The released ACh then binds to ACh receptors on sensory neurons, leading to plasma extravasation and neutrophil recruitment via CGRP and SP release and to neurogenic inflammation.

*P*. *aeruginosa* QSMs such as Oxo-HSL display bitter tastant characteristics and are agonists to Tas2R (22, 26), through which they induce epithelium-mediated respiratory reflexes dependent on cholinergic signaling (11). We therefore tested whether BC activation by bacterial signaling molecules induces neurogenic inflammation. Application of Oxo-HSL induced an EB extravasation that was significantly reduced in *Trpm5^–/–^* mice (Figure 7A). Furthermore, inhalation of either dose of Oxo-HSL led to increased extraepithelial neutrophils in control, but not in *Trpm5^–/–^* mice. In contrast, the number of the intraepithelial neutrophils was not different between *Trpm5^–/–^* mice and controls (Figure 7B). Since *P*. *aeruginosa* QSMs belong to different systems of bacterial quorum sensing, we then investigated whether PQS, a member of the *pqs* system, has an impact on neurogenic inflammation. Indeed, inhalation of different concentrations of PQS led to a significant increase in EB extravasation, which plateaued at 1 μM in WT mice and was absent using both 1 and 10 μM in *Trpm5^–/–^* mice (Figure 7C). Notably, the number of extraepithelial neutrophils was increased by inhalation of 1, 10, or 50 μM PQS in WT mice and abolished in *Trpm5^–/–^* mice, whereas there was no difference in intraepithelial neutrophil numbers at any concentration (Figure 7D). Furthermore, treating mice with supernatants of the mucoid *P*. *aeruginosa* strain NH57388A (24), isolated from a cystic fibrosis patient (the strain also used in the infection experiments), and supernatants from the *P*. *aeruginosa* strain PA103 (27), increased EB extravasation in WT mice (Figure 7E and Supplemental Figure 13A). EB extravasation to NH57388A was significantly reduced by 40% in *Trpm5^–/–^* mice compared with WT mice. Supernatants from the QSM-deficient *P*. *aeruginosa* mutant strain D8A6, a virulence-attenuated transposon mutant (a detailed description of this strain is given in the Supplemental Methods), induced less EB extravasation compared with PA103 (Supplemental Figure 13A). Extraepithelial neutrophil numbers significantly increased by 60% in WT mice treated with NH57388A supernatants (Figure 7F). In contrast, application of the NH57388A supernatant to *Trpm5^–/–^* mice led to a significant decrease in the extraepithelial number of neutrophils (Figure 7F), although numbers were already significantly higher in *Trpm5^–/–^* vehicle-treated mice in comparison with WT. The number of intraepithelial neutrophils was not altered in either group. In WT mice treated with PA103 supernatants, the extraepithelial number of neutrophils was significantly increased, while the intraepithelial neutrophil number did not change (Supplemental Figure 13B). In contrast to this, D8A6 supernatants did not change intra- or extraepithelial neutrophil numbers (Supplemental Figure 13B).

Since *P*. *aeruginosa* is a gram-negative bacterium, we then asked whether secreted molecules from gram-positive bacteria also exert effects on BC signaling. To test this, we treated mice with culture supernatants of 2 different *S*. *pneumoniae* strains, D39 (serotype 2) and PN36 (serotype 3), containing a mixture of bacterial compounds. Both supernatants induced EB extravasation in WT mice (Supplemental Figure 13C). However, EB extravasation evoked by PN36 and D39 supernatants was reduced in *Trpm5^–/–^* mice. These data demonstrate that BC-mediated responses are not limited to a single bacterial species and that BCs induce acute innate immune responses via Trpm5 signaling. The residual EB extravasation in *Trpm5^–/–^* mice in response to D39 and PN36 supernatants may be explained by the fact that the supernatants contain a complex mixture of bacterial molecules that activate additional signaling pathways besides the bitter signaling cascade in BCs. Neutrophil recruitment in response to inhalation of the 2 *S*. *pneumoniae* supernatants did not change the intraepithelial neutrophil numbers in the trachea (Supplemental Figure 13D). D39 supernatants increased extraepithelial neutrophils in WT but not in *Trpm5^–/–^* mice, whereas PN36 supernatants did not affect extraepithelial neutrophil numbers (Supplemental Figure 13D).

These results demonstrate that bacterial secretion factors induce a neurogenic inflammation in the airways that is elicited by stimulation of tracheal BCs and mediated by Trpm5 signaling (Figure 7G).

### BCs are needed to combat P. aeruginosa infections.

**Figure 8:**
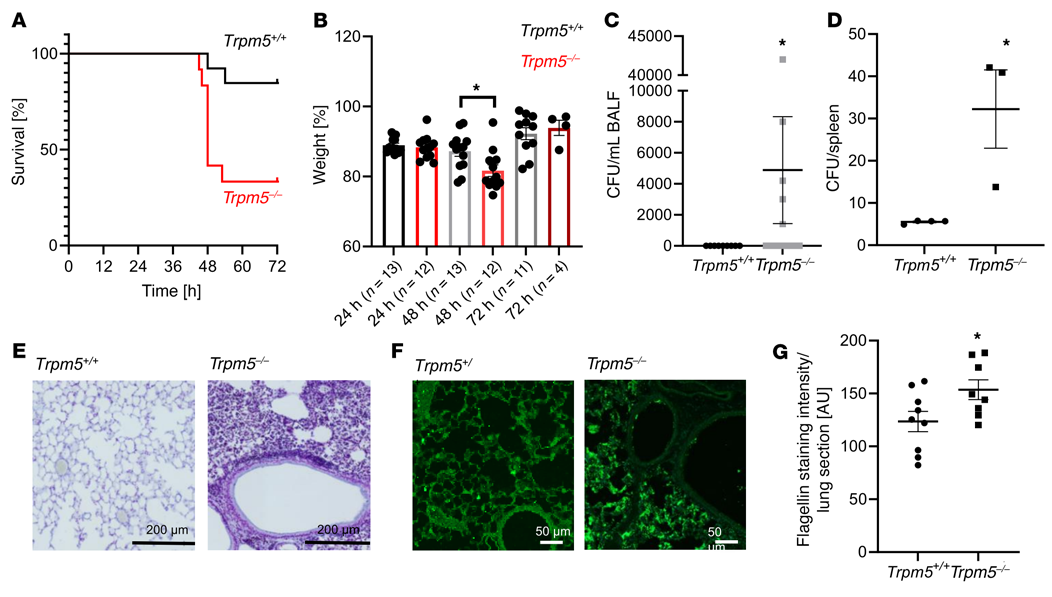
Infection of mice with the *P. aeruginosa* strain NH57388A. (**A**) Survival rate of WT (*Trpm5^+/+^*) and *Trpm5*-deficient (*Trpm5*^–/–^) mice revealed decreased survival rates in *Trpm5*^–/–^ mice after infection with *P*. *aeruginosa* NH57388A in the first 72 hours (*n =* 12–13 mice). (**B**) *Trpm5*^–/–^ mice showed significantly greater weight loss 48 hours after infection with *P*. *aeruginosa* NH57388A compared with WT controls. Note that the weight is shown only for the mice that survived. (**C**) Seventy-two hours after infection, an increased bacterial load (CFU) was detected only in bronchoalveolar lavage fluid (BALF) samples of *Trpm5*^–/–^ mice (*n =* 9–12 mice). (**D**) *Trpm5*^–/–^ mice had an increased bacterial load (CFU/mouse) in the spleen 2 days after infection (*n =* 3–4 mice). (**E**) Representative images of gram staining of WT and *Trpm5*^–/–^ lung sections 3 days after infection revealed more bacterial cells and destruction of alveoli in *Trpm5*^–/–^ animals. (**F**) Lung sections of WT and *Trpm5*^–/–^ mice stained for *P*. *aeruginosa* (green) 3 days after infection showed more flagellin staining, indicative of a higher number of bacteria and biofilm formation in *Trpm5*^–/–^ animals. Scale bars: 200 μm (**E**) and 50 μm (**F**). (**G**) Quantification of results in **F** (*n =* 8–9 mice). In **B**–**D** and **G**, data are depicted as mean ± SEM. *P < 0.05 by 2-tailed, unpaired Student’s *t* test.

Next, we set out to investigate whether the BC-mediated neurogenic inflammation represents a crucial early innate immune mechanism for protection against bacterial infections of the lower respiratory tract. For this, we performed in vivo infection experiments with the *P*. *aeruginosa* strain NH57388A in WT and *Trpm5^–/–^* mice. The animals were intubated and the tracheae were directly inoculated with the bacteria. In an experiment using 4.3 × 10^7^ colony forming units (CFU) to infect the mice (*n =* 15), mice had to be euthanized after 2 days due to severity of the infection. In 3 other experiments with lower infection doses (2.4 × 10^6^ CFU, 4.6 × 10^6^ CFU, and 6.2 × 10^6^ CFU), 11 of 13 control mice survived 3 days after infection, while 8 out of 12 of the *Trpm5^–/–^* mice died or had to be euthanized during the first 3 days (Figure 8A). Lastly, we infected additional WT mice and *Trpm5^–/–^* mice with 6.2 × 10^6^ CFU (*n =* 4) and kept them for 6 days. Only 1 *Trpm5^–/–^* mouse survived the infection. In comparison, only 1 WT mouse died over the course of infection. Overall, *Trpm5^–/–^* mice lost more weight 48 hours after infection compared with controls (Figure 8B) and exhibited a higher bacterial load 3 days after infection (Figure 8C). In the experiment with the higher inoculum (4.32 × 10^7^), we found higher loads of bacteria in the spleens of *Trpm5^–/–^* mice in comparison with controls, indicating a systemic spreading of the infection in *Trpm5^–/–^* mice (Figure 8D). No systemic effects were observed with the lower inocula; however, lung sections of the infected mice showed a much stronger bacterial load and inflammation, including lung structure destruction in *Trpm5^–/–^* mice compared with controls (Figure 8E). Staining for *P*. *aeruginosa* flagellin in these lungs revealed a high bacterial load, whereas *P*. *aeruginosa* staining in WT mice showed scarce labeling (Figure 8, F and G). Taken together, these results indicate that functional bitter signaling in tracheal BCs has a fundamental role to fight and clear *P*. *aeruginosa* infections.

**Figure 9:**
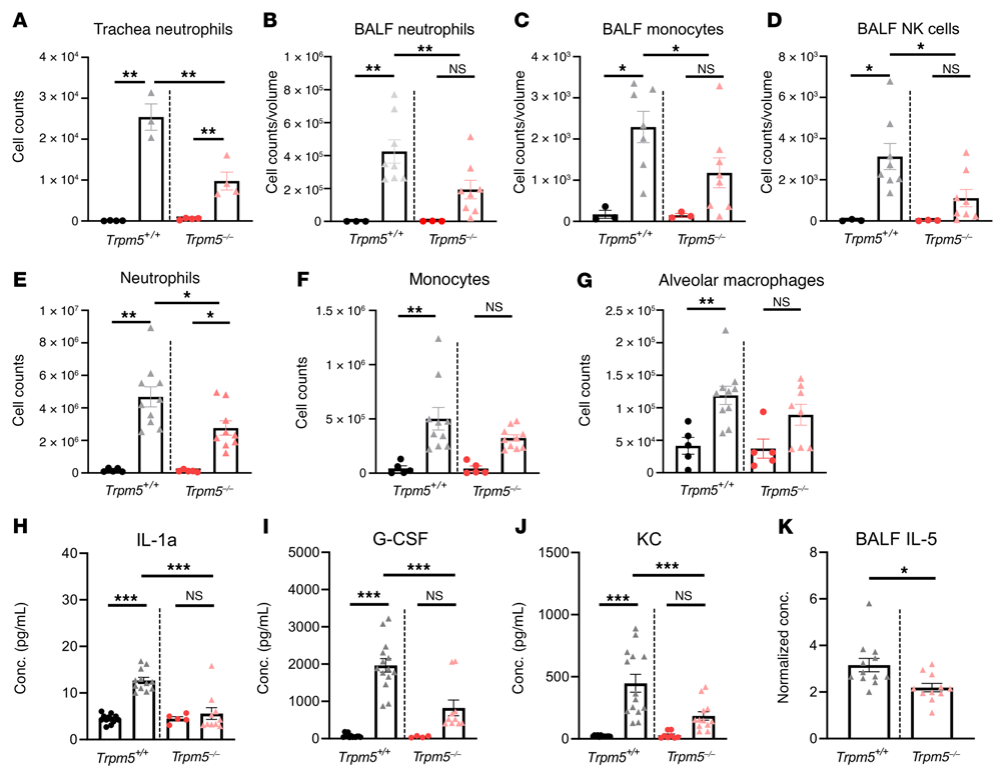
Characterization of the inflammatory response of mice infected with the *P. aeruginosa* strain NH57388A. (**A**) FACS analysis of neutrophils in *Trpm5^+/+^* and *Trpm5*^–/–^ mouse tracheae 4 hours after infection (triangles) and in controls (circles). (**B**–**D**) FACS analysis of neutrophils, monocytes, and natural killer (NK) cells in bronchoalveolar lavage fluid (BALF) of *Trpm5^+/+^* and *Trpm5*^–/–^ mice infected with *P*. *aeruginosa* NH57388A for 4 hours (triangles) and healthy controls (circles). (**E**–**G**) FACS analysis of neutrophils, monocytes, and alveolar macrophages of homogenized lungs of *Trpm5^+/+^* and *Trpm5*^–/–^ mice infected with *P*. *aeruginosa* NH57388A for 4 hours (triangles) and healthy controls (circles). (**H**–**J**) Multiplex ELISA of the cytokines IL-1α, G-CSF, and KC in plasma samples of mice before (circles) and after infection (triangles) with *P*. *aeruginosa* NH57388A for 4 hours. (**K**) ELISA of IL-5 of BALF of *Trpm5^+/+^* and *Trpm5*^–/–^ mice 4 hours after infection. In **A**–**K**, data are depicted as mean ± SEM (*n =* 3–14 samples). *P < 0.05; **P < 0.01; ***P < 0.001 by 1-way ANOVA followed by Bonferroni’s multiple-comparison correction.

FACS analysis of bronchoalveolar lavage fluid (BALF) cells, tracheal cells, and lung cells collected 4 hours after infection revealed differences between the immune cell populations in WT and *Trpm5^–/–^* mice. In the trachea, the increase in neutrophil number was significantly higher in WT than in *Trpm5^–/–^* mice (Figure 9A). An increased number of neutrophils, monocytes, and natural killer (NK) cells in BALF was observed in WT but not in *Trpm5^–/–^* mice (Figure 9, B–D). In the lung, the neutrophil number increased substantially in WT mice compared with *Trpm5^–/–^* mice (Figure 9E). In WT mouse monocytes, alveolar macrophages and NK cell numbers increased after infection, whereas in *Trpm5^–/–^* mice only NK numbers significantly increased (Figure 9, F and G, and Supplemental Figure 14A). The levels of dendritic and interstitial macrophage cell counts remained unaltered in the lungs of infected WT and *Trpm5^–/–^* mice (Supplemental Figure 14, B and C). Thus, in the early stage of infection, neutrophil recruitment to the lung as well as the migration of neutrophils, monocytes, and NK cells into the airspace is dependent on BC signaling.

Cytokine analysis of blood plasma samples collected 4 hours after infection revealed an increase in concentrations of IL-1α, IL-6, the CXC chemokine KC, G-CSF, MCP-1, and the CC chemokine eotaxin in WT but not in *Trpm5^–/–^* mice compared with concentrations before infection (Figure 9, H–J, and Supplemental Figure 14, D–F). MIP-1α expression increased after infection, although there was no difference between WT and *Trpm5^–/–^* mice (Supplemental Figure 14G). In general, levels of TNF-α, IL-5, and RANTES remained unaltered (Supplemental Figure 14, H–J). BALF samples collected 4 hours after infection showed higher IL-5, MCP-1, and MIP-1β concentrations in WT as compared with *Trpm5^–/–^* mice (Figure 9K and Supplemental Figures 14K and 15). These results indicate that Trpm5-dependent signaling in BCs is required for initiation of innate immune responses.

### BC stimulation leads to enhanced secretion of proteins associated with immune responses.

**Figure 10:**
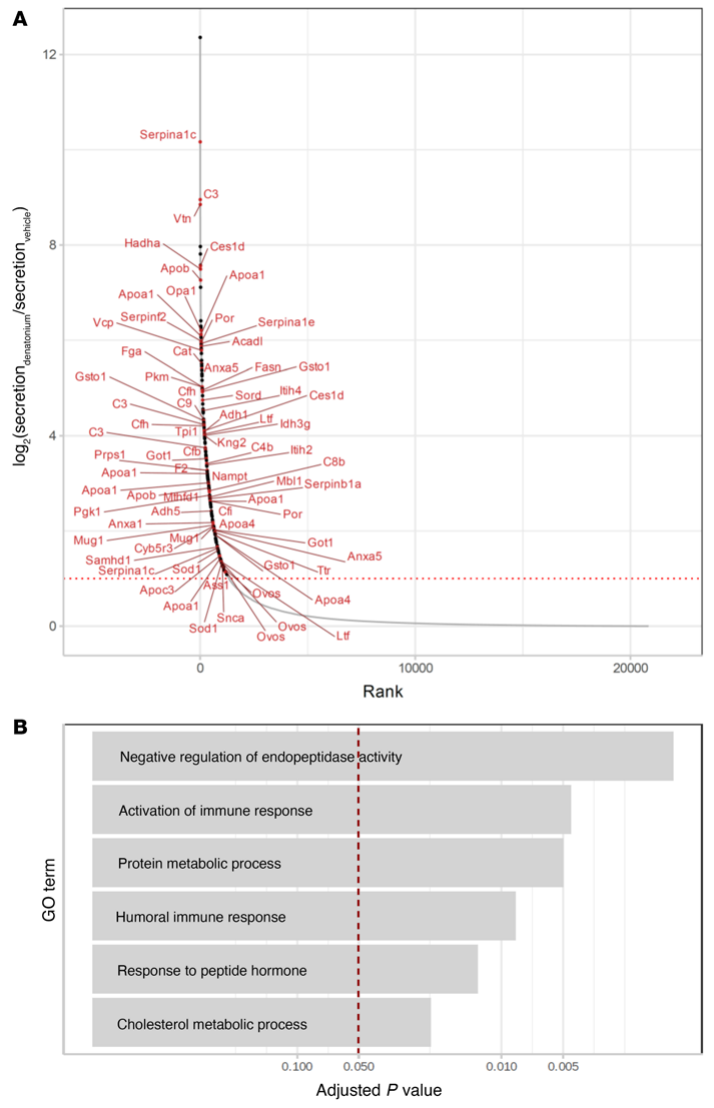
Secretome/proteome analysis of tracheae stimulated with denatonium. (**A**) Log_2_(fold change) of peptide enrichment in the supernatants (relative to their concentration in the tracheal cells) after denatonium treatment (*y* axis) and the corresponding rank (*x* axis; derived from Bayesian linear model, unadjusted, *n =* 6). Large dots denote peptides with supernatant concentration after denatonium treatment significantly upregulated both absolutely (in comparison with their concentration in the supernatants of vehicle-treated cells) and relative to their cellular concentration (FDR-corrected *P* value ≤ 0.05, log_2_[fold change] ≥ 1 in both tests). Peptides belonging to the biological processes shown in **B** are drawn in red and labeled by the names of their genes. (**B**) Gene Ontology Biological Processes significantly enriched (FDR-corrected Fisher exact test *P* value ≤ 0.05) among the peptides upregulated by denatonium treatment.

We next performed mass spectrometric analysis, comparing supernatants from tracheae stimulated with denatonium or vehicle, and found increased secretion of 226 out of 72,009 identified peptides (Figure 10A and Supplemental Tables 1 and 2). Gene enrichment analysis showed that many of these peptides belong to proteins underlying negative regulation of peptidase activity, such as serpins, and proteins involved in metabolic processes (Figure 10B and Supplemental Table 1). Strikingly, secretion of peptides representing proteins involved in the immune response, especially components of the complement system (C2, C3, C4b, C9, Cfh, Cfi, Cfb, Ltf, Mbl1, and C8b), which provides an intermediate link between the innate and adaptive immune system, was also highly upregulated (Figure 10A).

**Figure 11:**
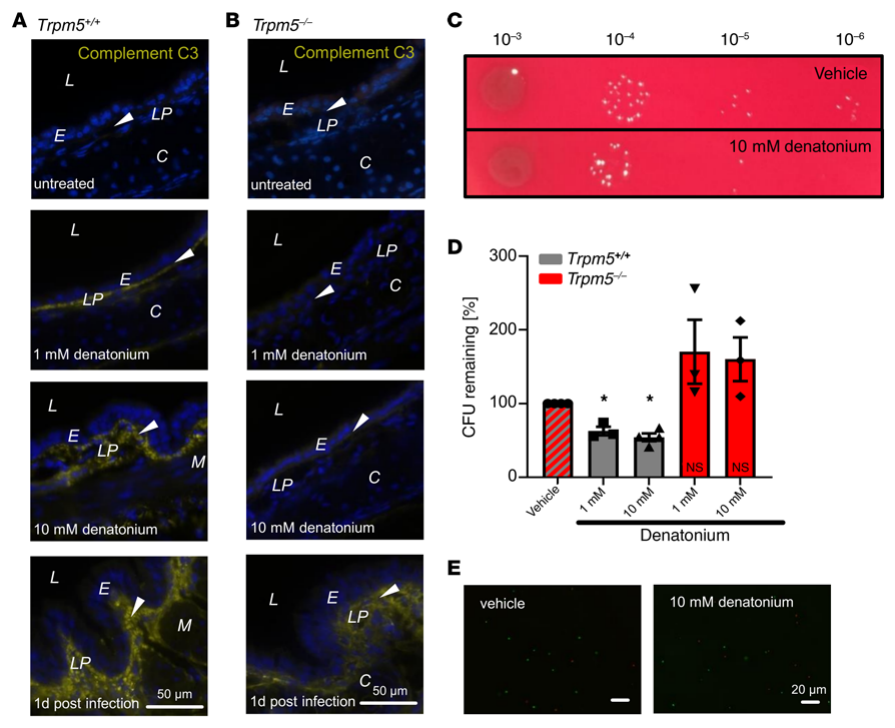
BC activation with denatonium induces an increase in the complement component C3 and reduces growth of *P. aeruginosa* strain NH57388A. (**A**) Staining for the complement component C3 (yellow) in naive or denatonium-treated (1 or 10 mM) mouse tracheal sections of *Trpm5^+/+^* mice or 1 day after infection with the *P*. *aeruginosa* strain NH57388A. (**B**) Staining for complement component C3 (yellow) in tracheal sections of naive denatonium-treated (1 or 10 mM) *Trpm5*^–/–^ mice or 1 day after infection with NH57388A. Blue: DAPI; arrowheads: basal membrane. L, lumen; E, epithelium; LP, lamina propria; C, cartilage; M, muscle. (**C**) *P*. *aeruginosa* killing assay. *P*. *aeruginosa* colonies after 2-hour incubation with supernatant from tracheae treated either with vehicle (RPMI, upper) or 10 mM denatonium (lower). (**D**) CFU of bacteria treated with supernatants from tracheae of WT and *Trpm5*^–/–^ mice after stimulation with 10 mM denatonium normalized to CFU of bacteria after RPMI (vehicle) treatment. Bars indicate the mean ± SEM with single values of *n =* 3–4 independent experiments. Data were analyzed with 1-way ANOVA followed by Bonferroni’s multiple-comparison correction. (**E**) Syto9/propidium iodide staining (see Supplemental Methods) of *P*. *aeruginosa* after incubation with supernatants from WT tracheae treated either with RPMI (vehicle, left) or 10 mM denatonium (right). Scale bars: 50 μm (**A** and **B**) and 20 μm (**E**).

We then stained tracheal sections of mice treated with denatonium (1 or 10 mM) for the complement component C3. WT mice revealed a strong C3 signal in the lamina propria when treated with denatonium (Figure 11A), which was even more pronounced in mice treated with 10 mM compared with 1 mM denatonium and absent in untreated mice. Interestingly, C3 staining was absent in *Trpm5^–/–^*mice treated with 1 and 10 mM denatonium (Figure 11B). C3 staining in tracheal sections from mice infected with the *P*. *aeruginosa* strain NH57388A on day 1 after infection revealed a strong signal in the subepithelial layer in WT tracheae, whereas the signal in *Trpm5^–/–^* mice was less pronounced (Figure 11, A and B).

### Activation of BCs inhibits bacterial growth.

Since denatonium increased components of the complement system in the trachea, we finally tested whether the molecules secreted by tracheal cells upon denatonium treatment suppress bacterial growth or promote bacterial death. Therefore, we incubated *P*. *aeruginosa* cultures with denatonium-treated tracheal supernatants. Bacteria incubated with supernatants of WT tracheae stimulated for 30 minutes with 1 or 10 mM denatonium showed a significant reduction in *P*. *aeruginosa* CFU by 37.2% and 45.7%, respectively, compared with vehicle-treated controls (Figure 11, C and D). Supernatants of tracheae of *Trpm5^–/–^* mice treated with the same denatonium concentrations did not affect bacterial growth (Figure 11D). Live/dead staining of the bacteria did not reveal a major increase in bacterial death in cultures incubated with supernatants of WT tracheae treated with 1 or 10 mM denatonium versus supernatants from vehicle-treated tracheae (Figure 11E), indicating that the activation of BCs exerts bacteriostatic effects.

## Discussion

We and others have recently shown that *P*. *aeruginosa* QSMs from the *pqs* system activate bitter taste signaling (2, 26). QSMs are important for bacterial growth and expansion. *P*. *aeruginosa* is a gram-negative opportunistic pathogen that can induce severe infections in immunocompromised patients or those with cystic fibrosis. Infections with the gram-positive *S*. *pneumoniae* cause severe pneumonia and are a major cause of morbidity and even mortality throughout the world. Antibiotic resistance to these bacteria has become a major therapeutic problem. Therefore, early steps of pathogen detection and stimulation of the host’s immune responses via BC activation that leads to bacterial killing by neutrophils and bacterial transport out of the airways by increased mucociliary clearance represent important mechanisms to fight these infections.

Here, we have shown that inhalation of the bitter substance denatonium induces neurogenic inflammation in the trachea characterized by plasma extravasation and neutrophil recruitment. Neutrophils represent the first line of defense in the onset of inflammation and are considered to be a major hallmark of neurogenic inflammation (28). Recently, we have shown that BCs are capable of releasing ACh in response to stimulation with denatonium, acting downstream on mAChRs and nAChRs in a paracrine manner (2, 29). Here, we demonstrate that BC-mediated neurogenic inflammation in the trachea depends on the transmission of cholinergic signaling from BCs to sensory nerves, since the plasma extravasation and neutrophil recruitment were abolished in the presence of the AChR antagonists mecamylamine and atropine. This is in line with previous observations that peptidergic sensory nerves projecting to the airways express functional α3 nAChRs (1). Nevertheless, a moderate residual effect was observed with 20 mM denatonium after inhibition of cholinergic signaling. The failure of denatonium to induce neurogenic inflammation in mice with depleted Trpa1^+^ sensory neurons emphasized the neurogenic nature of the observed inflammatory response.

Besides being cholinergic, BCs also synthesize cysteinyl leukotriene (CysLT) in response to aeroallergens (6, 30). Recently, CysLT receptor 2 expression was demonstrated to be present in approximately 40% of Trpv1^+^ neurons in mouse and human DRG where it contributed to itch in a model of dermatitis (31). However, it remains to be elucidated whether CysLTs are generated upon BC stimulation with bacterial products and, if so, whether they can consequently trigger the neural sensory network and attendant functions in the airways that BCs are poised to regulate via Trpm5-dependent cholinergic signaling (15, 16, 29). Nevertheless, since 20 mM denatonium led to an increase in [Ca^2+^]_i_ levels in Trpa1^+^ and Trpv1^+^ neurons and sensory neurons express Tas2R, it is plausible that the effects observed with high concentrations of denatonium or QSMs are a consequence of a direct stimulation of Tas2R in sensory neurons.

We have previously shown that BCs are approached by CGRP^+^ nerve fibers (1). Here, we have demonstrated that BC activation via bitter taste signaling leads to CGRP and SP release from sensory nerve endings. Although SP and CGRP are often colocalized (12, 32, 33), some recent studies in skin and urethra have documented both CGRP^+^SP^+^ and CGRP^–^SP^+^ fibers (34, 35). In our study, we found that tracheal BCs were commonly approached by CGRP^+^SP^+^ nerve endings. Supportively, the number of contacts between BCs and CGRP^+^ and SP^+^ nerve endings was comparable, and stimulation of BCs led to a comparable level of depletion of CGRP^+^ and SP^+^ nerve fibers contacting BCs. Additive and even opposing effects of CGRP and SP have been described in different tissues. In the cardiovascular system, CGRP is a more potent vasodilator than SP, whereas SP is more potent in inducing plasma extravasation (13, 36). Based on our findings, we suggest that CGRP and SP are involved in mediating the denatonium-induced acute neurogenic inflammation, although CGRP seems to play a crucial role. Both neuropeptides were released from the trachea upon stimulation with denatonium. However, denatonium completely failed to induce plasma extravasation and neutrophil recruitment only in those mice treated with the CGRP receptor antagonist CGRP_8–37_. The complete lack of response after inhibition of the CGRP receptors is probably due to a loss of induction of vasodilation in the arterioles, and thus to the lack of increase of the intramural pressure in the capillaries required for the SP-induced plasma extravasation (37). SP is also involved in neutrophil recruitment in skin (13, 38, 39). In the trachea, no recruitment of extra- and intraepithelial neutrophils and no plasma extravasation were found in the *SP^–/–^* mice even at 20 mM denatonium stimulation. Intriguingly, antagonizing the high-affinity receptor for SP, NK1-R, with its selective inhibitor CP96345, completely abolished the EB extravasation seen with 1 mM denatonium and strongly reduced the extravasation and recruitment of extraepithelial neutrophils at 10 and 20 mM concentrations. This residual response after NK1-R inhibition seems to arise at least partly from SP rather than from CGRP activity alone since this response is missing in *SP^–/–^* mice where CGRP is still expressed in sensory neurons (40). In our study, CGRP-dependent vasodilation was still present in *SP^–/–^* mice. This further underlines the fundamental role of CGRP in vasodilation, whereas SP is needed for the plasma extravasation and neutrophil recruitment in the presence of CGRP. Since depletion of the sensory innervation abolished the neurogenic inflammation, both BC-dependent and -independent SP and CGRP release involve sensory nerve fiber stimulation.

Previously, it has been shown that denatonium-induced mast cell degranulation in the nose is dependent on NK1-R activation (5). Our study demonstrates that in the trachea the BC-dependent extravasation and neutrophil diapedesis involves NK1-R, although this is independent of mast cell activation since stimulation of BCs did not evoke mast cell recruitment and degranulation. Very recently, SP was also found to mediate inflammatory responses by activation of the MrgprB2 receptor on mast cells independently of its canonical NK1-R (41). Such a scenario, which involves mast cell stimulation, is also conceivable for the SP-induced neurogenic inflammation in response to 20 mM denatonium in the trachea. It is tempting to speculate that high concentrations of SP due to direct stimulation of sensory nerve fibers on top of a cholinergic-, Trpm5-mediated SP release might act on the MrgprB2 receptor on mast cells and account for the BC-independent recruitment of neutrophils in our study.

Here, we demonstrate that the *P*. *aeruginosa* QSMs Oxo-HSL and PQS induce a taste signaling–dependent (Trpm5-dependent) neurogenic inflammation. Also, *P*. *aeruginosa* supernatants induce plasma extravasation and neutrophil recruitment that are at least partly dependent on Trpm5-mediated quorum sensing. The reduced plasma extravasation observed with the D8A6 supernatants lacking QSMs underlines the importance of the recognition of QSMs for induction of innate immune processes. The residual effect in *Trpm5^–/–^* mice and the effect of the *P*. *aeruginosa* QSM-mutant strain can be attributed to complementary pathogen detection pathways such as LPS recognition by TLR4. Trpm5-independent effects of denatonium have previously been described (42, 43). Very strikingly, *S*. *pneumoniae* culture supernatants of 2 different serotypes also led to plasma extravasation that was at least partially dependent on Trpm5. *S*. *pneumoniae* is supposed to have quorum-sensing systems, although different from those used by *P*. *aeruginosa* (44). We thus propose that BC-mediated induction of protective innate immune responses is a general mechanism needed to combat a broad range of gram-negative as well as gram-positive bacteria. Four hours after infection with the *P*. *aeruginosa* cystic fibrosis isolate NH57388A, the number of the recruited neutrophils in the tracheae of WT mice was significantly higher than in *Trpm5^–/–^* mice. Consistently, infections with NH57388A were more severe in *Trpm5^–/–^* mice, with more *Trpm5^–/–^* mice dying during the first 3 days of infection, further underlining the importance of BC-dependent bitter signaling in fighting airway infections. Additionally, *Trpm5^–/–^* mice showed intense *P*. *aeruginosa* staining, indicating biofilm formation. This might be due to an impaired activation of innate immune responses in these animals based on the lack of functional BC signaling leading to an impaired neurogenic inflammation and less mucociliary clearance (2) with reduced bacterial elimination. Several studies have shown that SP exhibits potent antimicrobial activity against various bacteria, including *P*. *aeruginosa,* *Staphylococcus aureus*, and *Escherichia coli* in vitro (45–47). It is plausible that SP acts on other immune cells besides neutrophils, since SP induces IFN-γ production in NK cells in the cornea, which helps to fight corneal *P*. *aeruginosa* infections (48). The lack of recruitment of NK cells that we detected in the BALF of *Trpm5^–/–^* mice 4 hours after *P*. *aeruginosa* infection and the higher mortality of *Trpm5^–/–^* mice when compared with controls strongly indicate a role of BC in regulation of NK function.

Here, we show for the first time to our knowledge that tracheal BCs directly impact the early innate immune responses in the lung, since an increase in recruitment of neutrophils, monocytes, and NK cells to the airspace was detected 4 hours after infection with NH57388A only in BALF of infected WT mice and not in *Trpm5^–/–^* mice. Yet, the failure of monocyte recruitment in *Trpm5^–/–^* mice might be a consequence of the lack of neutrophil recruitment (49). Since monocytes might differentiate into alveolar macrophages at later stages of inflammation (50), the reduced monocyte number might further result in reduced recruitment of alveolar macrophages. Recently, monocyte-derived alveolar macrophages were shown to conceal bacteria from the immune system to control inflammation (51). Therefore, it is tempting to speculate that BCs play an important role in preventing overshooting immune responses at later stages of inflammation.

Additionally, we show here that BCs impact the early cytokine response in an onset of *P*. *aeruginosa* infection. The increase in IL-1α, IL-6, KC, MCP-1, G-CSF, and eotaxin was abolished in *Trpm5^–/–^* mice after infection. Since KC and eotaxin play an essential role in the chemoattraction of neutrophils, and IL-6 and G-CSF are important for survival, proliferation, and differentiation as well as for neutrophil function, we assume that BCs are involved in the recruitment of neutrophils after stimulation not only by orchestration of sensory neuron–immune cell communication but also through changes in cytokine release (52–54). Moreover, supportive of our findings, IL-1α and G-CSF deficiency reduces immune responses and neutrophil recruitment as well as decreases survival and bacterial clearance after *P*. *aeruginosa* infection (55, 56). The Trpm5-dependent increase in eotaxin and IL-5 in BALF is congruous with recent findings that a subpopulation of airway BCs and their sister cells in the gut, tuft cells, activate group 2 innate lymphoid cells (ILC2s) (6, 57, 58). Very intriguingly, in the gut, ILC2-derived IL-5 results in the production of IgA, which is implicated in stomach protection by eliminating IgA-coated bacteria (59). ILC2s can be stimulated by CGRP and, more importantly, a subpopulation of ILC2s expresses *Calca*, which encodes CGRP (60), suggesting a possible role for these cells in BC-induced neurogenic inflammation. The significance of lymphoid-derived versus neural-derived CGRP needs to be addressed in future studies. Strikingly, the loss of tuft cells led to increased biliary infiltration of neutrophils that was linked to changes in the microbiota (61). Since an accumulation of inflammatory neutrophils was not observed in other tissues, it is suggestive of a biliary system–specific consequence and further implies that while chemosensory cells in different tissues all seem to be involved in regulating the immune system, the exact impact might be tissue specific.

Our secretome analysis revealed that after BC stimulation, peptides involved in negative regulation of endopeptidase activity, which consist of serpins and complement system components, notably C3, were highly increased. Neutrophils and the complement system are functionally linked (62). *C3^–/–^* mice show decreased survival in *P*. *aeruginosa* infections (63). Serpin B1 is necessary for neutrophil survival and for protection of the mature neutrophil reserve in the bone marrow (64, 65) and is essential for the clearance of *P*. *aeruginosa* (64). Another serpin, α-1 antitrypsin, has been shown to reduce lung tissue damage and to protect epithelial barrier integrity (66), thereby improving survival in *P*. *aeruginosa* infections. Here we directly link increased serpin and C3 expression to BC stimulation. This is supported by our findings that survival and bacterial clearance were disturbed in *Trpm5^–/–^* mice lacking functional BC signaling.

Since endopeptidases are also produced by and may contribute to virulence and growth of a wide range of bacteria, including *P*. *aeruginosa*, it is possible that the secreted negative regulators of endopeptidases observed in our study also inhibit bacterial endopeptidases. We propose that the reduction in bacterial growth in the presence of denatonium-treated tracheal supernatants in WT but not in *Trpm5^–/–^* mice is due to the secretion of bacteriostatic substances such as negative regulators of endopeptidase activity upon BC activation. Supernatants from human sinonasal airway epithelial cell cultures treated with denatonium decreased *P*. *aeruginosa* CFU by killing the bacteria (67). Lee and colleagues (67) attributed the bactericidal effects to low-molecular-weight antimicrobial peptides, while we observed an increase in complement system components. The differences between the bactericidal effects and the observed bacteriostatic effects in our study might originate from the physiological and functional disparity between upper and lower airways. Nevertheless, for the treatment of pneumonia, a meta-analysis did not find differences in cure rates when comparing bacteriostatic and bactericidal antibiotics (68).

In summary, we interpret the initial neurogenic inflammation as an essential acute protective mechanism. Our results provide evidence for a BC-dependent activation of neurogenic inflammation to rapidly eliminate inhaled pathogens via neutrophil recruitment, thereby ameliorating infection outcome. BC stimulation could be targeted as an endogenous pathway to mobilize the body’s defenses and, thus, as a possible alternative or addition to antibiotic treatment to overcome antibiotic resistance.

## Methods

### Animals.

All experiments were performed on adult (8–20 weeks old) C57BL/6J (The Jackson Laboratory, stock 000664), *Trpm5*-GCaMP3 (69), *Trpm5*-DTA (10), *Trpm5*-tauGFP (70), *Chat*-eGFP (21), *Trpm5^–/–^* (71), *lys*-*eGFP-ki* (Ly6G-GFP) (72) *Tac1^–/–^* (*SP^–/–^*) (40), *Trpa1*-tauGFP-DTR, *Trpa1*-GCaMP3, *Trpa1*-tauGFP (73), and *Chat-*eGFP/*Trpm5^–/–^* mice of either sex. A description of the mouse models is given in the Supplemental Methods. Animals were maintained in specified pathogen–free laboratory conditions. Handling and care of the animals was conducted in accordance with the German guidelines for the care and use of laboratory animals.

### Calcium imaging experiments.

Calcium imaging experiments were performed as described previously (2, 29). A detailed description of the Ca^2+^ imaging experiments in tracheae from *Trpm5*-GCaMP3 mice and primary sensory neurons is given in Supplemental Methods.

### Intratracheal administration of bitter substances and vascular permeability in the airways.

To address tracheal plasma extravasation, we developed a new experimental system. EB (Sigma-Aldrich) was injected into the retro-orbital sinus and then a cannula was inserted in the trachea and mice inhaled a single dose of denatonium (Supplemental Figure 1). A detailed description of the procedure is given in Supplemental Methods.

### Immunohistochemistry.

We performed immunohistochemistry on tracheal, lung, DRG, and JNC tissue sections as well as on tracheal whole-mount preparations. A detailed description of the antibodies used and the procedure is given in Supplemental Methods.

### SP and CGRP measurements.

Measurements of SP and CGRP were performed according to the manufacturers’ protocols of ELISA kits for SP and CGRP (R&D Systems and Bertin Pharma, respectively). For further details see Supplemental Methods.

### Intravital imaging.

Mice were anesthetized with urethane (1.5 g/kg i.p.), placed on a heated microscope stage (37°C), and the trachea was exposed as described previously (74). For further details see Supplemental Methods.

### Infection of mice with P. aeruginosa.

Mice were intratracheally infected with an inoculum of the mucoid *P*. *aeruginosa* cystic fibrosis isolate NH57388A (provided by Niels Hoiby, Department of Clinical Microbiology, Rigshospitalet, University of Copenhagen, Denmark) with slight modifications as described by Lawrenz and coworkers (75). For further details see the Supplemental Methods.

### FACS analysis.

A detailed description of the sample collection and preparation and the flow cytometry can be found in the Supplemental Methods.

### Cytokine measurements.

Cytokines were measured with a multiplex ELISA kit (Bio-Plex Pro Mouse Cytokine 23-plex Assay, Bio-Rad Laboratories GmbH) according to the manufacturer’s protocol. For further details see Supplemental Methods.

### Bacterial killing assay.

A description of the bacterial killing assay is provided in the Supplemental Methods.

### Live/dead stain.

Experiments were performed as previously described (76). For further details see Supplemental Methods.

### Mass spectrometric analysis of secretome and proteome.

The MS-based proteomics data are deposited at the ProteomeXchange Consortium (http://proteomecentral.proteomexchange.org) via the PRIDE partner repository with the data set identifier PXD018170. For further details see Supplemental Methods.

### Statistics.

Data of neurogenic inflammation, infections, and bacterial kill assay were analyzed with Prism software version 8 (GraphPad Software). The normality of data distribution was tested using the Shapiro-Wilk test. When more than 2 groups were compared, normally distributed data were then analyzed with 1-way ANOVA followed by Bonferroni’s correction for multiple comparisons. When 2 groups were compared, they were analyzed with the 2-tailed, unpaired Student’s *t* test. All data in the figures are expressed as single values with mean ± SEM. A multiple hypothesis–corrected *P* value of less than 0.05 was considered statistically significant.

### Study approval.

All experimental procedures were approved by the Animal Welfare Committee of the Regional Councils in Lübeck (V244-7224.121-9-34 (18-1/15), Würzburg (55.2.2532-2-98), and Homburg, Germany (69-2015, 04-2018, 25-2021).

## Author contributions

RN, SE, IG, NAW, MP, MIH, WN, SK, A Salah, AG, WB, TIK, LH, SMW, and GKC performed experiments. RN, SBE, IG, NAW, MP, A Salah, MIH, A Stukalov, SZ, and GKC analyzed the data. SZ, MB, PK, AP, UB, MIH, SBE, and GKC interpreted the results. PL, MW, CH, CB, RB, PR, AM, VC, VF, and TG provided experimental tools. SK, AW, FE, and UB generated the *Trpm5*-DTA, *Trpm5*-GCaMP3, *Trpm5*-tauGFP, *Trpa1*-tauGFP, *Trpa1*-GCaMP3, and *Trpa1*-DTR mice. GKC conceived and supervised the study. MIH, UB, and GKC wrote the manuscript. All authors approved the manuscript. The authorship order among the first authors was assigned according to seniority.

## Supplementary Material

Supplemental data

Supplemental table 1

Supplemental table 2

Supplemental video 1

Supplemental video 2

## Version 1 05/03/2022

In-Press Preview

## Version 2 07/01/2022

Electronic publication
