# Supplementary material for: Bitter taste signaling in tracheal epithelial brush cells elicits innate immune responses to bacterial infection
Source: J Clin Invest. 2022 Jul 1;132(13):e150951. doi: 10.1172/JCI150951 (PMC9246383; doi:10.1172/JCI150951)
Supplement: Supplemental data [file jci-132-150951-s075.pdf]

SUPPLEMENTAL METHODS

Mouse models

Chat-eGFP/*Trpm5*$^{-/-}$ mice were derived from breedings between *Chat*-eGFP and *Trpm5*$^{-/-}$ mice. *Trpa1*-tauGFP-DTR mice were produced by crossing *Trpa1*- tauGFP mice with Rosa26iDTR mice (1). The *Trpa1*-DTR mice were bred from *Trpa1*-IRES-Cre mice (2) and floxed Rosa26-DTR mice (1). The *Trpa1*-IRES-Cre mice express cre under the control of the endogenous Trpa1 promoter. The IRES-Cre cassette inserted downstream of the stop codon of the Trpa1 coding sequence results in the expression of Cre-recombinase in Trpa1-expressing cells. To generate this mouse, an 8.7-kb targeting construct comprising a total of 5.2 kb of homologous sequence flanking an IRES-Cre-PGK-Neomycin cassette was incorporated into the genome of ES cells by homologous recombination. This led to incorporation of the inserted cassette 5 base pairs downstream of the stop codon of *Trpa1*. Following targeting, correctly targeted stem cells were injected into blastocysts to generate the *Trpa1*-IRES-Cre knock-in mouse line. These mice were then crossed to floxed Rosa26-DTR mice which in turn leads to the expression of the diphtheria toxin receptor in the Rosa26-DTR mice via cre-mediated removal of floxed STOP signals. The *Trpa1*-GCaMP3 and the *Trpa1*-tauGFP mice were bred following the same principle. The *Trpa1*-IRES-Cre mice were bred with floxed Rosa26-GCaMP3 mice (3), leading to expression of the GCaMP3 calcium sensor from the *Rosa26* locus in Trpa1-expressing cells in order to generate the *Trpa1*-GCaMP3 mouse line. For the generation of the *Trpa1*-tauGFP mouse line, the *Trpa1*-IRES-Cre mice were bred with floxed Rosa26-tauGFP mice (4), leading to expression of tauGFP in Trpa1-expressing cells. The *Trpm5*-DTA mice (*Trpm5*-IRES-Cre-R26:lacZbpA$_{flox}$DTA) were generated from *Trpm5*-IRES-Cre (5) and R26:lacZbpAfloxDTA mice (6). In these mice the IRES-Cre-cassette inserted downstream of the stop codon of the Trpm5 coding sequence results in the expression of Cre-recombinase in Trpm5-expressing cells leading to expression of Diphteria Toxin A from the *Rosa26* locus.

Intratracheal administration of bitter substances and vascular permeability in the airways

Animals were anesthetized via intraperitoneal injection with 10% urethane (Sigma-Aldrich) or ketamine (85 mg/kg) / xylazine (15 mg/kg) (both obtained from Serumwerk Bernburg AG, Bernburg, Germany). Twenty minutes after the onset of anesthesia, 200 µl Evans blue (20 mg/kg) was injected into the systemic circulation through the retro-orbital venous sinus. A cricothyrotomy surgical approach was used for intratracheal administration of various substances to the lower airways. An incision of the skin was made in the ventromedial cervical region, and the salivary glands were positioned laterally to the trachea. The right infrahyoidal muscles were moved gently to the side of the trachea. Then, the median cricothyroid ligament of the larynx was separated by a small incision and a small breathing cannula was inserted into the tracheal lumen. 4 µl of vehicle or the following substances were administered into the trachea via the cannula: denatonium benzoate (1, 10 or 20 mM; Sigma-Aldrich), 3-Oxo-C12-homoserine lactone (3-Oxo-C12-HSL, 1 or 10 mM; Sigma-Aldrich), *Pseudomonas* quinolone signal (PQS, 1, 10 or 50 µM), as well as supernatants from three different strains of *Pseudomonas aeruginosa* (NH57388A, PA103 or D8A6) (D8A6 was kindly provided by Lutz Wiehlmann, Clinic for Pediatric Pneumology, Allerology and Neonatology, Hannover Medical School) or two different strains of *Streptococcus pneumoniae* (D39 or PN36). A detailed description on the preparation of the bacterial supernatants can be found below. Animals were sacrificed 30 min after treatment using isoflurane (Abbott, Wiesbaden, Germany) and transcardially perfused with 4% paraformaldehyde (PFA). Tracheal segments (1-5 cartilage ring of the trachea, 6-11 and 12-bifurcation) were dissected, postfixed in 4% PFA, washed, incubated with 18% sucrose and shock-frozen in liquid nitrogen.

Trpa1-tauGFP-DTR mice were injected intraperitoneally with 20 ng/kg diphtheria toxin (DT) on three consecutive days. Intratracheal administration of denatonium or vehicle was performed as described above on the seventh day after the last DT injection. The NK1 receptor antagonist CP96345 (2.5 µg/g mouse body weight, Tocris) was administered intraperitoneally 30 min and the CGRP receptor blocker $CGRP_{8-37}$ (800 ng, GenScript Biotech, Leiden, The Netherlands) 24 h and 2 h before the intratracheal stimulation with denatonium. These doses of CP96345 and $CGRP_{8-37}$ were previously proven to be effective (CGRP: (7), CP: (8–10)). The combination of mecamylamine and atropine (1 mM for each substance) was applied intratracheally 5 min prior to denatonium application. 30 min after intratracheal instillation of the substances (denatonium, QSM or supernatants), the animals were sacrificed using isoflurane and perfused transcardially with 4% PFA. Tracheal segments (1-5 cartilage rings of the trachea, 6-11 and 12-bifurcation) were dissected, post-fixed in 4% PFA for 1 h and washed for 2 h with 10 mM phosphate buffer. Finally, the tissue was incubated in a solution of 18% sucrose/10 mM phosphate buffer for cryoprotection for 4 h and shock-frozen in liquid nitrogen.

Cryosectioning and Evans blue extravasation

Evans blue fluorescence was evaluated on 10 µm-thick serial cryosections of the middle part of the trachea (segment 6-11) using a Texas Red filter (585/29 nm excitation; 624/40 nm emission) on an epifluorescence microscope (Axioplan 2 imaging, Zeiss). The distance between two sections was 100 µm and altogether 5 sections per animal (n = 3-5) and condition were included in the evaluation. Tracheal ring images were acquired and Evans blue intensity of the experimental groups was recorded with the same exposure time in each total tracheal ring for all groups. The fluorescence of each ring was then quantified using the ImageJ software by measuring the area of the fluorescence of a composite picture of the tracheal ring.

Immunohistochemistry

For immunofluorescent staining of tracheal and lung slices of Trpm5-tauGFP, Trpm5$^{-/-}$, ChAT-eGFP or C57Bl6/J mice the tissue samples were blocked for 1 h and then incubated over night with primary antisera (single or in combinations) against Trpm5 (11–13), Trpm5 794 (generated in house), GNAT3 (covalab, pab73402, Cambridge, UK), DCLK1 (abcam, ab31704, Cambridge UK, RRID: AB_873537), CD31 (Dianova, Hamburg, Germany, DIA310, RRID: AB_2631039), CGRP (Abbiotec, Escondido, USA, 250602, RRID: AB_2068659), SP (Santa Cruz Biotechnology, Dallas, USA, SC-21715, RRID: AB_628299), complement C3 (Novus Biologicals, Wiesbaden, Germany, NB200-540, RRID: AB_10003444) and *P. aeruginosa* (abcam, ab74980, Cambridge, UK, RRID: AB_1281081) at room temperature followed by three washing steps in PBS (Supplemental Table 3). Sections were then incubated for 1 h with the respective secondary antisera coupled to either Cy3 or Cy5, washed one time with PBS and incubated for 10 min with DAPI followed by three washing steps in PBS. The specificity of GNAT3, DCLK1, CGRP and SP antibodies was tested by pre-absorption of the antibodies with the corresponding peptides. Trpm5 antibody specificity was verified on *Trpm5*$^{-/-}$ mouse tissue (Supplemental Figure 16 and 17). Images from flagellin stained lung sections obtained from infection experiments with wt (n=9) and *Trpm5*$^{-/-}$ mice (n=8) were acquired at the same image settings using an Axio Scan.Z1 slide scanner (Zeiss). The mean fluorescence intensity in left lung lobe coronal sections was analyzed by the ZEN3.0 software (Zeiss). For whole-mount stainings employing CD31, CGRP, and SP antibodies, tracheae were processed as described previously (14).

For neutrophil staining, tracheal sections were incubated for 1 h at room temperature with 0.1% bovine serum albumin, 10% horse serum, and 0.2% Tween-20 to block non-specific protein binding sites followed by an incubation step in anti-Ly6G-FITC antiserum (Invitrogen, Thermo

Fisher Scientific, 11-5931-85, RRID: AB_465315) at room temperature. Numbers of infiltrated neutrophils in tracheal ring sections of denatonium treated and controls as well as from animals treated with 3-oxo-C12- HSL, PQS, NH57388A, PA103, D8A6, D39 or PN36 supernatants were counted manually using an epifluorescence microscope (Supplemental Figure 3B). Gram-staining of lung cryo-sections was conducted with a commercial kit according to the manufacturer's protocol (Carl Roth, Karlsruhe, Germany).

Trpa1-tauGFP, *Trpa1*-GCamP3 and *Trpa1*-tauGFP-DTR mice were euthanized with an overdose of isoflurane and perfused transcardially with ice-cold phosphate buffer (0.1 M PB; 0.2 M Na_2HPO_4 and 0.2 M NaH_2PO_4), followed by Zamboni fixative. Dissected dorsal root ganglia (DRG) and jugular-nodose-complexes (JNC) were immediately post-fixed for 30 min in Zamboni, washed in 0.1 M PB and then treated with 30% sucrose in 0.1 M PB. Cryo-sections of DRG and JNC (10 μm) were mounted on slides and stored at -20°C. Nonspecific binding sites were blocked with 5% fetal bovine serum, 0.3% TritonX-100 in PBS for 2 h, followed by over-night incubation with primary antisera (single or in combinations) against rabbit anti-TRPV1 (1:2,000; Alomone ACC-030, RRID: AB_2313819), CGRP (Abbiotec, Escondido, USA, 250602, RRID: AB_2068659), SP (Santa Cruz Biotechnology, Dallas, USA, SC-21715, RRID: AB_628299) at room temperature followed by three washing steps in PBS. Sections were then incubated for 1h with the respective secondary antisera, counter-stained with DAPI and mounted with a cover-slip.

Confocal Laser Scanning Microscopy Analysis

Z-stacks of either vehicle or 1 mM Denatonium treated tracheal whole-mount preparations were taken with a Leica TCS SP5 Confocal Laser Scanning Microscope (Leica Microsystems, Wetzlar, Germany). Each stack was scanned with an XY-resolution of 512 × 512 and a size of 387.5 x 387.5 x 30 μm, respectively, starting from the luminal side of the tracheal epithelium.

Analysis from one to four image stacks from randomly chosen regions of interest from the membranous part of the tracheae were performed using Imaris 9.9.0 (Oxford Instruments) or ImageJ (NIH) software.

Quantification of brush cell–nerve fiber contact sites and volume analysis of tracheal sensory nerve fibers

For the quantification of brush cell – nerve fiber contact sites image stacks containing at least 15 brush cells were examined with ImageJ. Within a stack all brush cells were manually examined whether they have a contact site with either CGRP+ or SP+ sensory nerve fibers. The number of brush cells with contacts to sensory nerve fibers was then normalized to the total number of brush cells in each stack. Volumes of CGRP+- and SP+- nerve fibers were quantified from the 3D reconstructed surfaces. Each surface volume was normalized to its corresponding stack volume.

Calcium imaging experiments

Trpm5-GCaMP3 mice were killed by inhalation of an overdose of isoflurane followed by aortic exsanguination and the trachea was dissected and cut into three pieces and opened longitudinally. Tracheal pieces were placed into the recording chamber (Warner Instruments RC-27LD, Hamden, USA) coated with ELASTOSIL®RT 601 A (Wacker Silicones, Burghausen, Germany) and continuously perfused with experimental solutions pre-heated to 33°C. A confocal imaging system microscope (Zeiss LSM710, Jena, Germany) with a water immersion objective (W Plan Apochromat 20x/1.0 DIC VIS-IR, Zeiss) and an argon laser (488 nm) was used to excite and an emission filter at 493-598 nm to collect fluorescent signals from tracheal cells expressing GCaMP3 with a 2 Hz frame rate. Solutions were based on standard

Tyrode III solution consisting of (in mM): 130 NaCl, 10 HEPES, 10 Glucose, 5 KCl, 1 $MgCl_2$, 8 $CaCl_2$, 10 sodium pyruvate, 5 $NaHCO_3$ in which 1 mM, 10 mM or 20 mM denatonium or 1 mM C12-Oxo-HSL were freshly dissolved before the experiment. Denatonium was applied for 5 min followed by a 5 min washing step with Tyrode solution. C12-Oxo-HSL was applied for 3 min followed by a washing step with Tyrode solution and application of 100 µM ATP. Acquired video files underwent spatial drift correction using cross-correlation function using slightly modified freely available script (http://onlinedigeditions.com/publication/?i=223321&p=40) from MATLAB (MathWorks®). Further analysis was performed using ImageJ and Prism (GraphPad).

For calcium imaging experiments of isolated tracheal brush cells, tracheae were treated and measurements were performed as previously described (12, 15).

Neuronal culture and calcium imaging of neurons

Sensory ganglia (DRG and JNC) were dissected from freshly sacrificed mice and collected in DH10 medium on ice (90% DMEM/F-12, 10% FBS, 100 U/ml penicillin, 100 mg/ml streptomycin, Gibco). Dissected DRGs/JNCs were digested in a protease solution (5 mg/ml dispase, Roche; 1 mg/ml collagenase type I, Sigma) in HBSS without Ca^{2+} and Mg^{2+} (Gibco) for 30 min at 37°C. Afterwards mechanical disruption was applied to allow dissociation into single neurons, which were collected by centrifugation at 220*g*. Pelleted neurons were resuspended in DH10 medium and plated onto glass coverslips coated with poly-L-lysine (0.1 mg/ml, Sigma) and laminin (10 mg/ml, Gibco). Neurons were cultured in an incubator at 37°C (without CO_2) for 2 h before proceeding to calcium imaging. Neurons were loaded with Fura-2 (Invitrogen, Thermo Fisher Scientific) in Locke's buffer (NaCl, 136 mM; KCl, 5.6 mM; $MgCl_2$, 1.2 mM; $CaCl_2$, 2.2 mM; NaH_2PO_4, 1.2 mM; glucose, 10 mM; pH 7.4) for 30 min at 37°C. After washing, Fura-2-loaded neurons were imaged at 340 and 380 nm excitation to detect the intracellular calcium. Cells were considered as responding if their fluorescence ratio

was increased by at least 20% compared to baseline. A 340/380-nm ratio of light was used from a DG4 wavelength switching xenon arc lamp (Sutter Instruments, Novato, California, USA). An Orca Flash 4.0 camera was used for fluorescence detection (Hamamatsu, Herrsching, Germany). Analyses were performed using the NIS-Elements software (Nikon Instruments, Amsterdam, Netherlands). Neurons isolated from TRPA1-GCamP3 mice were measured directly after washing with Locke's buffer, the changes in GCamP3 fluorescence were measured at emission of 525 nm before and after application of capsaicin and cinnamaldehyde (Sigma-Aldrich, Taufkirchen, Germany).

SP and CGRP measurements

Mice were euthanized with an overdose of isoflurane followed by aortic exsanguination. Tracheae were explanted and collected in 200 µl buffer consisting of (in mM): 136 NaCl, 5.6 KCl, 10.7 Glucose, 10 Hepes, 1 $MgCl_2$, 2.2 $CaCl_2$. Tracheae were stimulated with 1 mM or 20 mM denatonium for 5 min at 37°C and then kept on ice. Tissues were homogenized using a tissue ruptor (Qiagen, Hilden, Germany) and centrifuged at 1500 g for 5 min at room temperature. The clear supernatant was further processed using commercial Substance P and CGRP ELISA kits (R&D systems, Biotechne, Abingdon, UK and Bertin Pharma, Montigny-le-Bretonneux, France, respectively) according to the manufacturers' protocols. Immunoassay plates were then assessed photometrically with a microplate reader (Bio-Rad, Laboratories GmbH, Feldkirchen, Germany) and results were presented in pg/ml.

Intravital imaging

Mice were anaesthetized with urethane (1.5 g/kg i.p., Sigma-Aldrich), placed on a heated microscope stage (37°C) and the trachea was exposed as described previously (16). To ventilate the mice (n = 4), we opened the trachea near the thyroid cartilage, inserted a flexible cannula into the first third of the trachea and fixed it with a surgical suture. The cannula was connected

to a FlexiVent ventilator (SCIREQ INC., Montreal, Canada) and the mouse was ventilated with 10 ml/kg room air with a frequency of 120 min^{-1}. Images of the trachea were recorded using a TriM Scope II multiphoton microscope (LaVision BioTec GmbH, Bielefeld, Germany) using a XLPLNXWMP2 N 25x dip-in objective, NA 1.05 (Olympus, Hamburg, Germany) and two titanium-sapphire lasers (InSight and Mai Tai both from Newport Spectra Physics GmbH, Darmstadt, Germany). Ventilation triggered images were taken to generate time series to analyze cell movement. 3D-stacks were recorded during 2 s pressure holds of the ventilator that followed after a deep inflation maneuver using the DIEPH protocol. GFP and autofluorescence were excited at 740 nm and emitted light was detected in two spectral channels (<495 nm and 495-560 nm). Second harmonic generation signals were excited at 1100 nm and emitted light was detected in the spectral channel 495-560 nm.

Preparation of bacterial supernatants

S. pneumoniae PN36 serotype 3 strain A66 (NCTC 7978) and D39 serotype 2 (NCTC 7466) from cryo-stocks were grown on Columbia agar supplemented with 5% sheep blood (BD Biosciences, Heidelberg, Germany) for 8 - 9 h (37°C, 5% CO2). Single colonies were inoculated in Todd-Hewitt broth containing 0.5% yeast extract (BD Biosciences). PN36 cultures were additionally supplemented with 10% heat inactivated fetal calf serum. Bacteria were grown until mid-logarithmic phase at 37°C, centrifuged (3,100 rpm, 10 min, w/o brake) and the supernatant was collected and stored at -80°C until further usage. *P. aeruginosa* strains NH57388A and D8A6 were grown from cryo-stocks on Trypticase Soy Agar II plates with 5% sheep blood (BD Bioscience). Single colonies were transferred to LB medium (Sigma Aldrich) and incubated over night at 37°C (16 h and 12 h respectively). Supernatants were then obtained by sterile filtration (non-pyrogenic, 0.2 µm filter, Sarstedt) of the bacterial cultures. *P. aeruginosa* PA103 (ATCC29260) was directly inoculated from cryo-stocks into Trypticase Soy

Broth (TSB) (BD Bioscience) and incubated overnight at 37°C in a shaking incubator. Supernatant was collected after centrifugation and stored at -80°C.

Infection of mice with *P. aeruginosa*

Preparation of the inoculum for infection: 20 ml of Lysogeny Broth (LB) medium was inoculated with the mucoid *P. aeruginosa* cystic fibrosis isolate NH57388A (kindly provided by Niels Hoiby, Department of Clinical Microbiology, Rigshospitalet, University of Copenhagen, Denmark). On the next day, the bacterial suspension was mixed with alginate dissolved in 0.9% NaCl. Single-use aliquots from this inoculum were prepared on the day of the experiment. Controls were plated on LB-agar plates to verify the concentration of the inoculum.

Intratracheal infection: Mice were infected by intubation with slight modifications to the previously described method (18). Mice were anesthetized by intraperitoneal injection with xylazine (6 – 8 mg/kg, Bayer, Leverkusen, Germany) and ketamine (90 – 120 mg/kg, Zoetis, Berlin, Germany). Mice were intubated and 40 µl bacteria were applied. The mice were inoculated once and then kept alive for different time periods. In total, ten independent experiments were performed, six over a time period of 4 h with inocula of $1.7*10^6$ CFU, $1.8*10^6$ CFU, $3.6*10^6$ CFU, $3.8*10^6$ CFU, $4.4*10^6$ CFU, $9.3*10^6$ CFU (in total n=13 mice per group), one over a time period of two days with a higher dosed inoculum ($4.3*10^7$ CFU, n=4 wt and and n=3 *Trpm5*$^{-/-}$ mice), two experiments for three days with inocula of $4.6*10^6$ CFU or $2.4*10^6$ CFU (in total n=9 mice per group), and one over a time period of six days ($6.2*10^6$ CFU, n=5 mice per group). For determination of the CFU in bronchoalveolar lavage fluid (BALF) six additional experiments were conducted over a time period of three days with inocula of $1.3*10^6$ CFU, $1.44*10^6$ CFU, $1.5*10^6$ CFU, $1.74*10^6$ CFU, $1.78*10^6$ CFU and $3*10^6$ CFU (in total n=9 wt and n=12 *Trpm5*$^{-/-}$ mice). Survival of Trpm5$^{-/-}$ and wild type mice is depicted by Kaplan-Meier curves.

Animal preparation: At the end of the experiment, the mice were killed by an overdose of Xylazin/Ketamin and blood was taken by intracardiac puncture with an EDTA-coated needle. 20 µl blood samples were used for the quantification of leukocytes. EDTA-plasma samples were stored at -80°C until further use. The lungs and other organs were removed, and treated according to subsequent analysis.

Bacterial load: Bacterial load was determined from dilutions of homogenized (Ultra-Turrax) tissue. The homogenate was diluted and plated on LB-agar plates by the drop-plate method (10 µl/spot) (19). The bacterial plates were incubated at 37°C in a humidified incubator to prevent drying in case of slow growing strains. The CFU/ml was quantified by counting all colonies visible in 10 spots per dilution for which 10-50 colonies were produced.

FACS analysis

Sample collection and preparation: Bronchoalveolar lavage fluid (BALF) was obtained by lavaging the whole lungs with 1ml of PBS with 0.1 mM EDTA and 1% fetal bovine serum (FBS) through a tracheal cannula. The lungs were perfused with PBS through the right ventricle then the trachea and lungs were removed carefully for processing. Single cell suspensions were made from the lungs and the trachea by cutting them into small pieces then incubating them in digestion solution containing collagenase type II (1 mg/ml, Gibco) and DNase I (1 µl/ml, Invitrogen) for 60 min at 37°C then filtered through 70 µm cell strainer (Falcon, BD bioscience). BALF and the single cell suspensions were then incubated with ACK RBCs lysis buffer (Thermo Fisher Scientific, Karlsruhe, Germany). Total cell counts and viability percentages were calculated using an automatic cell counter (NucleoCounter® NC-200™, Chemomatec, Kaiserslautern, Germany). BALF and the single cell suspensions were fixed with 1% paraformaldehyde in PBS solution. The cells were then collected and resuspended in FACS buffer (0.1mM EDTA plus 1% fetal bovine serum (FBS) in PBS).

Flowcytometry: BALF and single cell suspension from the lungs and trachea were incubated in Fc-blocking solution (CD16/CD32 Antibody, 1µg/10^6cells, Thermo Fisher Scientific) for 30 min at 4°C then stained using the following fluorochrome conjugated antibodies: F4/80-PE-Cy7 (RRID: AB_469653), CD45-PerCP-Cy5.5 (RRID: AB_1107002), CD11b-FITC (RRID: AB_464935), CD86-PE (RRID: AB_465768), CD11c- Super Bright 436 (RRID: AB_2722928) and LY6G-APC (RRID: AB_2573307) for 45 min at 4°C. All fluorochrome conjugated antibodies were purchased from e bioscience (Thermo Fisher Scientific). The data were acquired with BD FACSverse machine and analysed using BD FACSuite™ Software (BD Biosciences). The analysis of the data was performed using Flowjo v10.6.2.

Cytokine measurements

Cytokines were measured with a multiplex ELISA kit (Bio-Plex pro mouse cytokine 23-plex assay, Bio-Rad Laboratories GmbH, Feldkirchen, Germany) according to the manufacturer's protocol. Briefly 50 µl of beads were added to the pre-wet wells and washed two times. Then 50 µl of plasma samples, controls or blanks were added and incubated 30 min at room temperature with shaking. After washing three times, 25 µl of detection antibody was added and incubated for 30 min at room temperature with shaking followed by three more washing steps. Then wells were incubated with 50 µl streptavidin-PE for 10 min at RT with shaking and again washed three times. Finally, 125 µl resuspension buffer was added and the plate was shaken for 30 sec, followed by data acquisition.

Mass Spectrometry Analysis of Secretome and Proteome

To investigate the secretome of denatonium-stimulated tracheae, mice were euthanized using isoflurane and tracheae were explanted. The tracheae of six mice were stimulated for 30 min with either 1 mM denatonium or vehicle solution (Ringer's lactate solution). Supernatant and trachea epithelium were collected and protein purification and enrichment for liquid

chromatography coupled to MS (LC-MS/MS) analysis was performed as described previously in Hughes et al. (20) by using paramagnet beads (GE Healthcare, Cat. 09-981-123) and performing the Single-Pot Solid-Phase-enhanced Sample Preparation (SP3) method.

In brief, supernatant and tracheal epithelium samples were diluted 3-fold in lysis buffer (6% SDS, 30 mM EDTA, 30 mM EGTA and 60 mM NaOH in 60 mM HEPES buffer at pH 8.5) and incubated for 15 min. Afterwards, 100 µg of beads and 10% of trifluoroethanol were added and incubated for 10 min before sonicating for 10 min in a Bioruptor (10 cycles: 30 s ON, 30 s OFF) at high settings. The solution was neutralized by adding formic acid and incubated for 5 min at 95 °C. Proteins were alkylated with 30 mM iodoacetamide (IAA) for 30 min followed by quenching with 13 mM dithiothreitol (DTT).

Protein binding to the beads was achieved by acidifying the solution with 1% formic acid to a pH range of 2-3 by immediate addition of 50% (v/v) acetonitrile (ACN). The solution was incubated for 10 min at RT and mixing at 700 rpm. The beads were washed twice with 70% (v/v) ethanol and twice with 100% ACN. Beads were resuspended in 20 mM HEPES (pH 8) and bound proteins digested with trypsin (Promega) and LysC (Wako Chemicals, USA) overnight at RT and 500 rpm mixing. The Bead-Peptide mixture was homogenized by sonification for 6 min in a Bioruptor (6 cycles: 30 s ON, 30 s OFF) at high settings. The peptides were eluted in 2% DMSO and sample volume reduced using a SpeedVac Concentrator (Thermo Fisher Scientific) and subsequent resuspension in 2% ACN, 0.1% Trifluoroacetic acid (TFA) and 2% DMSO. Total peptide concentration was quantified and adjusted to 0.5 mg/ml; 1.5 µg total peptide was analyzed by LC-MS/MS on a Q Exactive HF instrument (Thermo Fisher Scientific). To enhance the identification of short peptides the samples were run twice, once including and once excluding singly charged ions. Raw mass-spectrometry data were processed with the MaxQuant software (version 1.5.7.9) and then further analyzed by a combination of in-house R (version 3.5) and Stan (version 2.14) scripts. The gene set enrichment analysis was

performed using the topGO Bioconductor package (version 2.38.1) with the Gene Ontology annotations provided by the org.Mm.eg.db package (version 3.10).

Data processing of LC-MS/MS samples: Raw mass-spectrometry data were processed with the MaxQuant software (version 1.5.7.9) using the built-in Andromeda engine and mouse protein sequences (UniprotKB release 2016_08 including isoforms and unreviewed sequences) for peptide identification. Carbamidomethylation was set as fixed and methionine oxidation and N-acetylation as variable modifications, using a mass tolerance of 4.5 ppm for the precursor ion and 0.5 Da for the fragment ions. Minimal peptide sequence length was set to 5. To improve peptide quantitation, matching between MS runs, which utilized the same MS protocol settings, was enabled. Search results were filtered with a false discovery rate (FDR) of 0.01 for peptide and protein identifications. All other MaxQuant settings were set to their default values.

Statistical analysis of MS data: MaxQuant output (evidence.txt) was processed by a combination of in-house R (version 3.5) and Stan (version 2.14) scripts.

To account for the variation in the analyzed biological sample amounts, sample preparation, MS protocol and performance, the peptide intensities were first normalized. Specifically, approximately 5000 peptides with enough quantitative information were randomly selected, and, by solving the least absolute deviation optimization problem, we have inferred the intensity-correcting multipliers α_i (one for each MS experiment) that minimized the absolute deviation of peptide intensities across all MS experiments:

$$\sum_{i,j} \left| \alpha_i Intensity_{j,i} - median_k\left(\alpha_k Intensity_{j,k}\right) \right| \to min,$$

where i (or k) is the index of MS experiment and j – the peptide index. For the analysis of peptide concentration changes, their normalized intensities were fit to a generalized linear Bayesian random-effects statistical model for each peptide individually (peptides that share the same sequence, but have different post-translational modifications were treated as different entities). The model used horseshoe priors for the model effects, and natural logarithm as a

linking function for peptide intensities. The MS intensities were set to follow Laplace distribution. The experimental design matrix of the GLM model was (using R GLM formula notation):

$$\log_2(\text{intensity}) \sim 1 + \text{treatment*extraction} + \text{msprotocol},$$

where "treatment" denotes the effect of cells treatment (denatonium or vehicle), "extraction" denotes the peptide extraction protocol (either from whole cells or supernatant lysates), and "msprotocol" denotes the MS settings (inclusion or exclusion of singly-charged ions).

The posterior distributions of model parameters were inferred using Hamiltonian Markov Chain Monte Carlo (MCMC) method (Stan version 2.14), employing 8 parallel MCMC chains, 2000 warmup and 2000 sampling iterations taking every 4th sample from each chain. The statistical significance (*p*-value) of a hypothesis that given model effect X is different from zero (min $(P(X \leq 0), P(X \geq 0))$), was calculated by approximating the posterior distribution from MCMC samples using Gaussian kernel with Silverman's rule-of-thumb bandwidth. Multiple hypothesis correction of P-values was done using Benjamini-Yakutieli method (p.adjust(method="BY") R function). The model effect X was considered significant, if $|\text{median}(X)| \geq 1$, and the corresponding corrected P-value was below 0.05. The gene set enrichment analysis was done using topGO Bioconductor package (version 2.38.1) with the Gene Ontology annotations provided by org.Mm.eg.db package (version 3.10). Classical Fisher's exact test and Holm's multiple hypothesis correction method were used to test for the gene set enrichment significance. All analysis scripts are available upon request.

Live/dead-Stain

Experiments were performed as previously described (21). Briefly, bacteria were grown in overnight culture and diluted to an OD_{600} of 0.1 directly prior to the incubation with supernatants of explanted wt tracheae that were treated either with vehicle (RPMI) or denatonium (1 mM or 10 mM) for two hours. Viability of bacteria was examined using the

LIVE/DEAD *BacLight* Bacterial Viability Kit (Invitrogen). Equal amounts of Syto9 and Propidium Iodide were mixed and 1:10 diluted with RPMI. 5 µl was then added to 40 µl of bacteria and incubated for 5 minutes at room temperature. Stained bacteria were visualized using epifluorescence microscopy.

Bacterial killing assay

P. aeruginosa (strain NH57388A) was grown in Tryptic Soy Broth (TSB, BD Biosiences) for 16 h followed by two washing steps with Roswell Park Memorial Institute (RPMI, Thermo Fisher Scientific) 1640 medium. Bacteria were diluted to an OD_{600} of 0.1. Simultaneously, tracheae from either TRPM5$^{+/+}$ or TRPM5$^{-/-}$ mice were explanted and incubated in 120 µl RPMI medium containing different concentrations of denatonium for 30 min at 37°C. 50 µl of each tracheal supernatant was mixed with 50 µl of bacteria. Samples were incubated in a shaker (150 rpm) for 2 h at 37°C. Afterwards, 10-fold serial dilutions (up to 10^{-6}) were prepared and added to Trypticase Soy Agar plates with 5% sheep blood in a dropwise manner. CFU/ml were determined after 24 h of incubation at 37°C.

Quantitative real-time PCR analysis

Quantitative real-time PCR was performed as previously described (17). Briefly, RNA was isolated from DRGs and JNC tissue using an RNAeasy Isolation Kit (Qiagen, Hilden, Germany) in accordance with the manufacturer's protocol, and cDNA was synthesized using Superscript II Reverse Transcriptase (Thermo Fisher Scientific). A CFX Connect System was utilized to quantify gene expression with SYBR Green Master Mix using default settings (Bio-Rad Laboratories Inc., Hercules, CA, USA), . The reaction cycles were as follows: 95°C for 600 s, then 95°C and 59°C for 20 s each for 45 total cycles, followed by 65°C for 5s, and finally 95°C for 5s. Fluorescence was quantified following each cycle. (Cq) values for each sample were determined using Bio-Rad CFX Software. Generated Cq values were used to determine copy

number and ratio analysis was undertaken by comparing these values to those generated for Gapdh. A summary of the oligonucleotides (Eurofins Genomics, Ebersberg, Germany) that were used to amplify the respective genes can be found in Supplementary Table 4.

PCR

Epithelial tracheal cells, HEK293 cells transfected with the T2R108, DRGs and JNC tissues were collected for RNA extraction using an RNAeasy Isolation Kit (QIAGEN) in accordance with the manufacturer's protocol, cDNA was synthesized using Superscript II Reverse Transcriptase (Thermo Fisher Scientific). AmpliTaq Gold master mix was used to amplify a total 2 µL of DNA per sample in accordance with the manufacturer's protocol. The reaction cycles were: 95°C for 720 s, then 95°C, 60°C, and 72°C for 20 s each for 40 total cycles, followed by 72°C for 420 s.

SUPPLEMENTAL INFORMATION

The quorum sensing deficient *P. aeruginosa* strain D8A6

The D8A6 strain is a *Tn*5 transposon mutant of *P. aeruginosa* strain TBCF10839, a cystic fibrosis isolate taken from a CF patient at Hannover Medical School in 1983. A description of the generation of transposon mutants from TBCF10839 can be found in Wiehlmann *et al.* (22). While the wildtype strain TBCF10839 is able to survive in neutrophil granulocytes, D8A6 lost this ability. The D8A6 mutant is quorum sensing negative, secreting neither C12- and C4-homoserinelactones of the *las* and *rhl* systems nor the quinolone signal PQS, although some PQS precursor molecules could be detected. In accordance with the lack of QS signals, the D8A6 mutant displayed lack of protease, elastase and siderophore secretion and other quorum sensing dependent traits such as impaired haemolysis and reduced motility. The gene affected by the transposon insertion in mutant D8A6 is a predicted ORF with a size of nearly 3 kb, with an incompletely elucidated function. The ORF 5PG21 in the genomic island PAGI-5 is inactivated in this mutant (23). This ORF is not part of the *P. aeruginosa* core genome. PAGI-5 is characterized by a set of several dozen conserved genes ORFs which are also annotated as type IV secretion system-like components. The conserved ORFs are supposed to be involved in mobilisation, transfer and genomic integration of the genomic island with regard to their descendance from mobile DNA elements. The question, how the knock-out of such a gene can affect the quorum sensing system and cause the described phenotypes, is still not answered, but an surprising influence of this D8A6/5PG21 gene from the accessory genome on this core regulatory system can be postulated. The mutation of the 5PG21-ORF as the actual cause of the observed effects was proven by subsequent generation of target mutants.

Characterization of the *Trpa1*-DTR mouse model

In order to characterize the Trpa1$^+$ neurons, we performed Ca^{2+} imaging experiments of primary neurons isolated from the Th1-Th5 dorsal root ganglia (DRG) of *Trpa1*-GCaMP3 mice (Supplemental Figure 7A-C). *Trpa1*-GCaMP3 neurons showed an increase in $[Ca^{2+}]_i$ to 200 μM cinnamaldehyde and to 100 nM capsaicin, a TRPA1 and TRPV1 agonists respectively. TRPA1$^+$ neurons accounted for approximately 27 % of the DRG neurons in *Trpa1*-GCaMP3 mice. Trpa1$^+$ neurons were almost completely abolished in DRGs from *Trpa1*-tauGFP-DTR mice after DT treatment as estimated by the absence of GFP-fluorescent neurons as well as from transcript levels evaluated by real-time PCR (Supplemental Figure 7D-G). Only a very low number of neurons was found to respond to cinnamaldehyde or capsaicin after the depletion of Trpa1-expressing neurons (Supplemental Figure 7H-I). Staining of DRGs with a TRPV1 antibody revealed approximately 80% decrease in the number of neurons expressing TRPV1 after the depletion of Trpa1$^+$ neurons innervating the airways (Supplemental Figure 7J-K). Moreover, CGRP$^+$/SP$^+$ sensory nerve fibers in the trachea were almost completely depleted in *Trpa1*-tauGFP-DTR mice after DT treatment (Supplemental Figure 9). Taken together, this shows that the Trpa1-DTR/DT mouse model is suitable for studying sensory nerve-mediated responses from the respiratory system.

SUPPLEMENTAL REFERECNES

1. Buch T et al. A Cre-inducible diphtheria toxin receptor mediates cell lineage ablation after toxin administration. *Nat. Methods* 2005;2(6):419–426.

2. Bernal L et al. Odontoblast TRPC5 channels signal cold pain in teeth. *Sci. Adv.* 2021;7(13):eabf5567.

3. Paukert M et al. Norepinephrine controls astroglial responsiveness to local circuit activity [Internet]. *Neuron* 2014;82(6):1263–1270.

4. Wen S et al. Genetic identification of GnRH receptor neurons: A new model for studying neural circuits underlying reproductive physiology in the mouse brain. *Endocrinology* 2011;152(4):1515–1526.

5. Kusumakshi S et al. A binary genetic approach to characterize TRPM5 cells in mice. *Chem. Senses* 2015;40(6):413–425.

6. Brockschnieder D, Pechmann Y, Sonnenberg-Riethmacher E, Riethmacher D. An Improved Mouse Line for Cre-Induced Cell Ablation Due to Diphtheria Toxin A, Expressed From the Rosa26 Locus. *Genesis* 2006;44(2):322–327.

7. Baral P et al. Nociceptor sensory neurons suppress neutrophil and γδ T cell responses in bacterial lung infections and lethal pneumonia. *Nat. Med.* 2018;24(4):417–426.

8. Hon YL, Bhatia M. Effect of CP-96,345 on the expression of adhesion molecules in acute pancreatitis in mice. *Am. J. Physiol. - Gastrointest. Liver Physiol.* 2007;292(5):1283–1292.

9. Engel MA et al. Opposite effects of substance P and calcitonin gene-related peptide in oxazolone colitis [Internet]. *Dig. Liver Dis.* 2012;44(1):24–29.

10. Bhatia M, Zhi L, Zhang H, Ng SW, Moore PK. Role of substance P in hydrogen sulfide-induced pulmonary inflammation in mice. *Am. J. Physiol. - Lung Cell. Mol. Physiol.* 2006;291(5):896–904.

11. Kaske S et al. TRPM5, a taste-signaling transient receptor potential ion-channel, is a ubiquitous signaling component in chemosensory cells. *BMC Neurosci.* 2007;8(1):49.

12. Hollenhorst MI et al. Tracheal brush cells release acetylcholine in response to bitter tastants for paracrine and autocrine signaling. *FASEB J.* 2020;34(1):316–332.

13. Krasteva G et al. Cholinergic chemosensory cells in the auditory tube. *Histochem. Cell Biol.* 2012;137(4):483–497.

14. Krasteva G et al. Cholinergic chemosensory cells in the trachea regulate breathing. *Proc. Natl. Acad. Sci.* 2011;108(23):9478–9483.

15. Kumar P, Scholze P, Fronius M, Krasteva-Christ G, Hollenhorst MI. Nicotine stimulates ion transport via metabotropic b4 subunit containing nicotinic acetylcholine receptors. *Br. J. Pharmacol.* [published online ahead of print: 2020]; doi:10.1111/bph.15270

16. Kretschmer S et al. Autofluorescence multiphoton microscopy for visualization of tissue morphology and cellular dynamics in murine and human airways. *Lab. Investig.* 2016;96(8):918–931.

17. Keshavarz M et al. Caveolin-3 differentially orchestrates cholinergic and serotonergic constriction of murine airways. *Sci. Rep.* 2018;8(1):1–18.

18. Lawrenz MB, Fodah RA, Gutierrez MG, Warawa J. Intubation-mediated intratracheal (IMIT) instillation: A noninvasive, lung-specific delivery system. *J. Vis. Exp.* 2014;(93):1–5.

19. Herigstad B, Hamilton M, Heersink J. How to optimize the drop plate method for enumerating bacteria. *J. Microbiol. Methods* 2001;44(2):121–129.

20. Hughes CS et al. Ultrasensitive proteome analysis using paramagnetic bead technology. *Mol. Syst. Biol.* 2014;10(10):757.

21. Lee RJ et al. T2R38 taste receptor polymorphisms underlie susceptibility to upper respiratory infection2012;122(11):4145–4159.

22. Wiehlmann L et al. Functional genomics of Pseudomonas aeruginosa to identify habitat-specific determinants of pathogenicity. *Int. J. Med. Microbiol.* 2007;297(7–8):615–623.

23. Munder A, Wölbeling F, Klockgether J, Wiehlmann L, Tümmler B. In vivo imaging of bioluminescent Pseudomonas aeruginosa in an acute murine airway infection model. *Pathog.*

Dis. 2014;72(1):74–77.

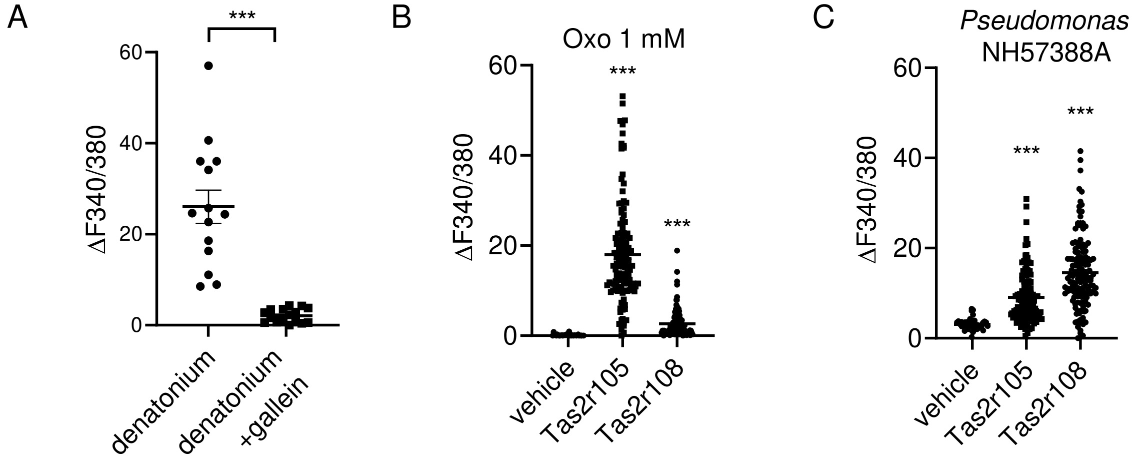

Supplemental Figure 1: Measurements of $[Ca^{2+}]_i$ in brush cells and HEK293 cells transfected with taste receptors. **A)** $ChAT^+$ brush cells responded to 1 mM denatonium with an increase in $[Ca^{2+}]_i$ levels (n=14 cells of 3 mice) that was inhibited by 10 µM gallein (n=16 cells of 3 mice), an inhibitor of $G_{\beta\gamma}$-subunits. **B-C)** Measurements of $[Ca^{2+}]_i$ in HEK293 cells transiently transfected with the mouse Tas2r105 or Tas2r108. **B)** Cells transfected with Tas2r105 (n=130) or Tas2r108 (n=143) reacted to the application of 1 mM N-(3-Oxododecanoyl)-L-homoserine lactone (Oxo) with an increase in intracellular Ca^{2+}. **C)** Cells transfected with Tas2r105 (n=130) or Tas2r108 (n=143) reacted to the application of supernatants of the mucoid cystic fibrosis patient derived *P. aeruginosa* strain NH57388A with an increase in intracellular Ca^{2+}. **A-C)** Data are shown as single values and mean ± SEM and were analyzed with the unpaired Student's t-test (A) or one-way ANOVA followed by Bonferroni's multiple comparisons (B-C). ***p<0.001

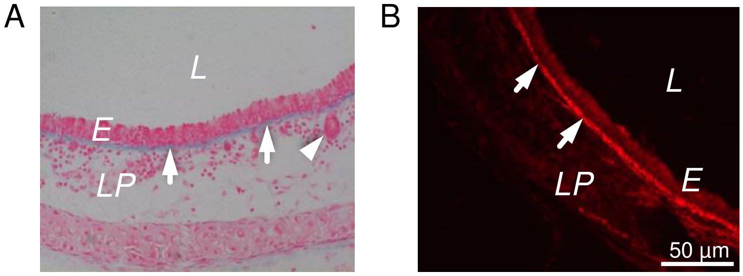

Supplemental Figure 2: Evans blue staining evaluated by light and fluorescence microscopy. Representative tracheal sections from an in vivo experiment with 10 mM denatonium. **A)** Arrows indicate the Evans blue dye bound to collagen fibers in the basal membrane. Arrowhead indicates a blood vessel. Evans blue labeling was counterstained with nuclear fast red staining as follows: sections were rinsed in distilled water for 1 minute and incubated with nuclear fast red solution for 10 minutes followed by a washing step of 1 minute in distilled water. Sections were then dehydrated by subsequent incubation in 70%, 96% and 100% ethanol, each for 1 minute, and then incubated for 2x 5 min in Xylol. Stained sections were then mounted and analyzed by light microscopy. **B)** Detection of the Evans blue dye using fluorescence microscopy and Texas Red filter is very sensitive. Arrows point to the bright Evans blue fluorescence in the basal membrane. Red staining is also observed in collagen fibers in the *lamina propria* of the trachea. L: lumen, E: epithelium, LP: *lamina propria*.

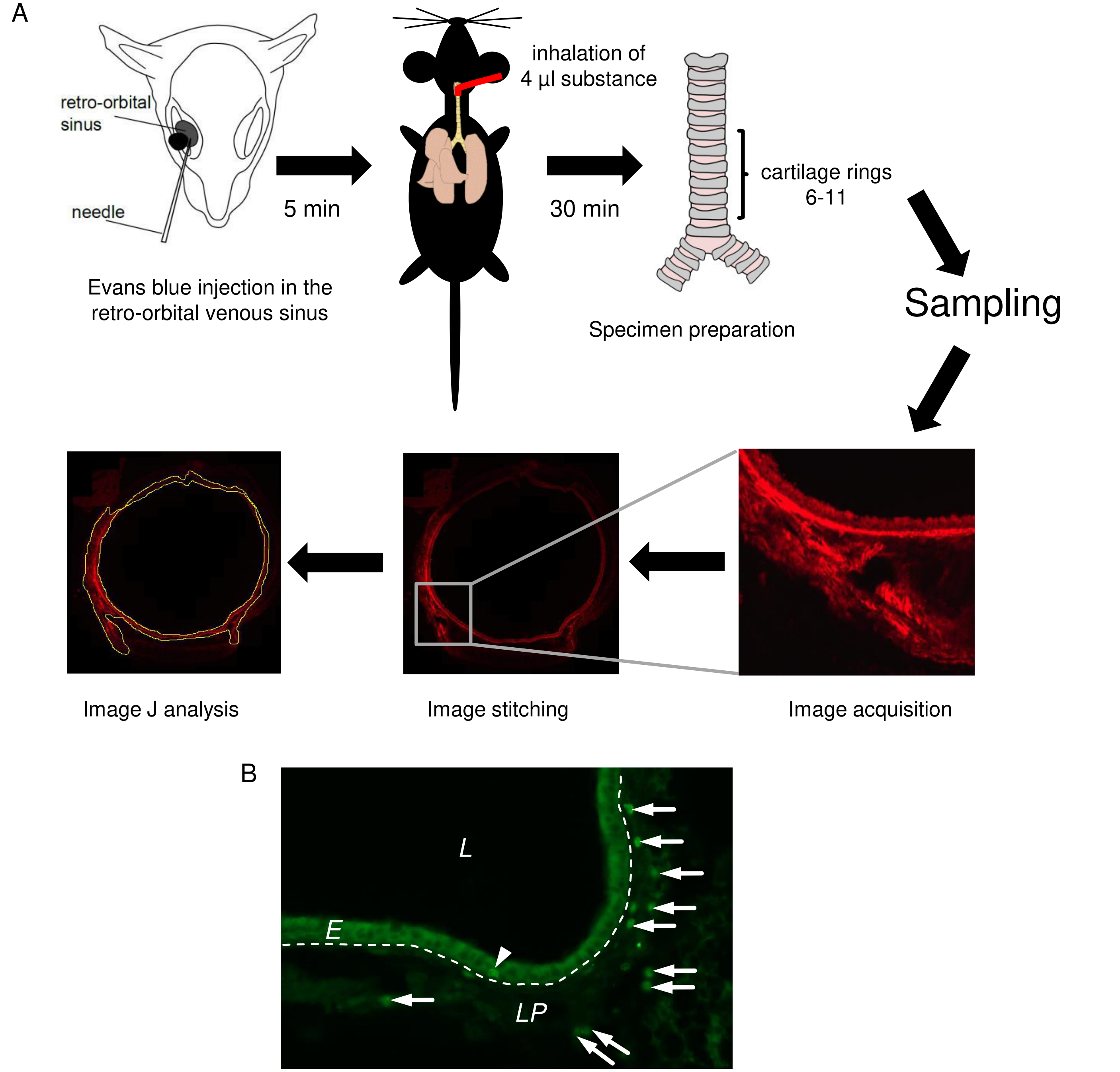

Supplemental Figure 3: A) Workflow diagram for the evaluation of Evans blue extravasation. Evans blue was delivered by a retro-orbital venous sinus injection. After cricothyrotomy, a pre-bent tubing (inhalation canula) was inserted into the trachea. Mice received an intratracheal instillation of 4 µl of substance or vehicle. After 30 min, the animals were euthanized and transcardially perfused with 4% PFA, the trachea was explanted and divided into three parts. The middle part (consisting of tracheal cartilage rings 6-11) was processed for embedding and sectioning. Evans blue fluorescence was evaluated in at least 5 histological sections/animal. The distance between two sections was 100 µm. Images from tracheae treated with different concentrations of a given substance or vehicle were taken at the same exposure time under a fluorescence microscope using a Texas red filter. Quantitative analyses of fluorescence intensity were conducted with ImageJ. **B)** Example of a tracheal section used for counting neutrophils. Arrowhead shows an intraepithelial neutrophil and arrows show extraepithelial neutrophils in the *lamina propria*. E: epithelium, LP: *lamina propria*, L: lumen

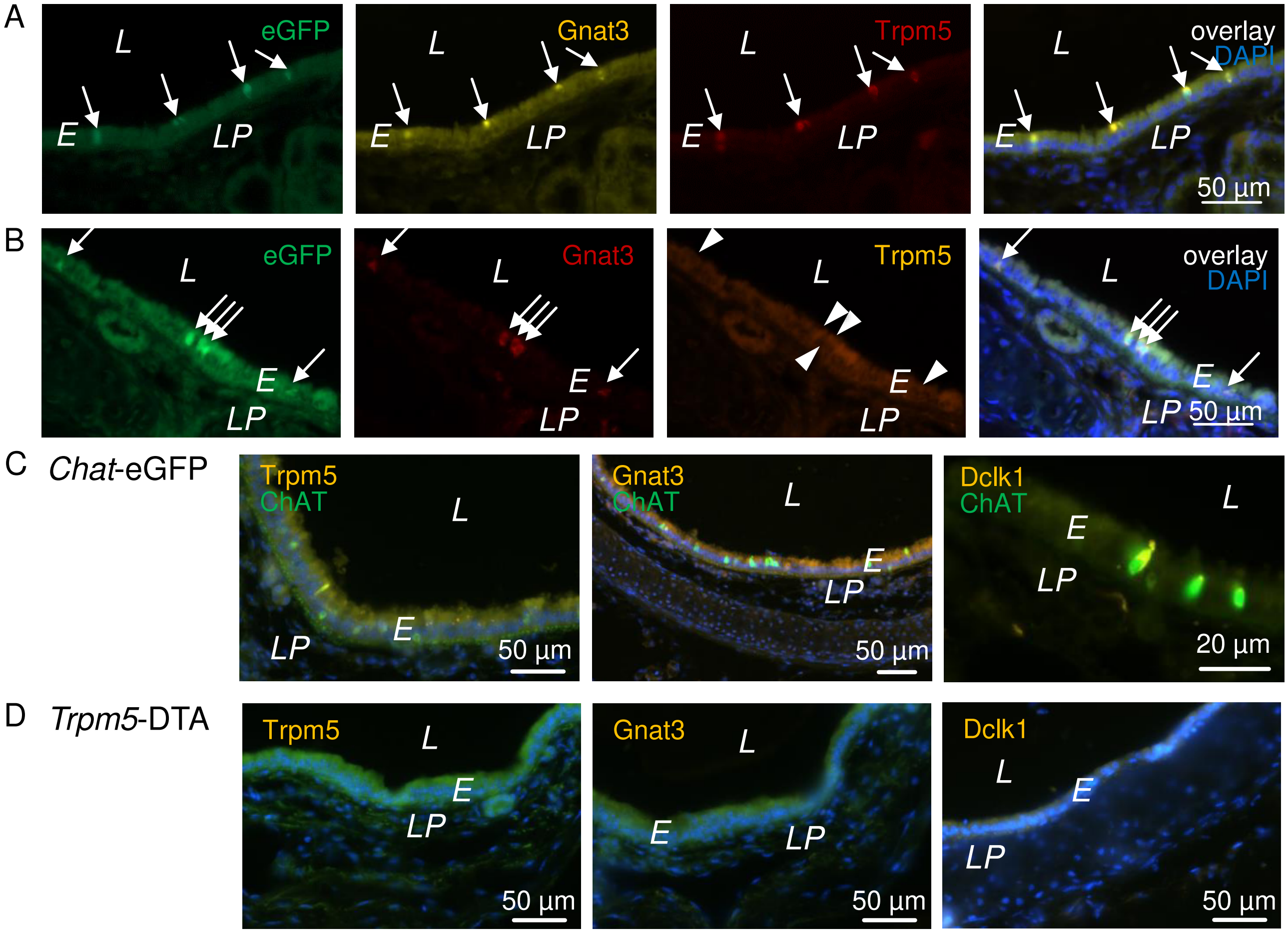

Supplemental Figure 4: A-B) Immunostaining of tracheal sections from *Chat*-eGFP and *Chat*-eGFP-*Trpm5*$^{-/-}$ mice. **A)** Chat-eGFP cells (arrows, green) are positive for Gnat3 (orange) and Trpm5 (red). **B)** Immunhistochemistry on tracheal sections from *Trpm5*$^{-/-}$/*ChAT*-eGFP mice confirmed the absence of Trpm5 (orange, arrowheads) in BC cells (green, arrows). Gnat3^{+} (red, arrows) cells colocalized with the eGFP signal in BC. **C-D)** Confirmation of the BC deficiency in *Trpm5*-DTA mice. Immunofluorescence of tracheae from *Trpm5*-DTA and *ChAT*-eGFP (control) mice. **C)** Control mice. *Chat*-eGFP^{+} cells (green) are labelled for the BC markers Trpm5, Gnat3 and Dclk1 (orange). **F)** *Trpm5*-DTA mice. No staining was observed for the BC markers Trpm5, Gnat3 and Dclk1 (compare to C). **A-D)** L: lumen, E: epithelium, LP: *lamina propria*.

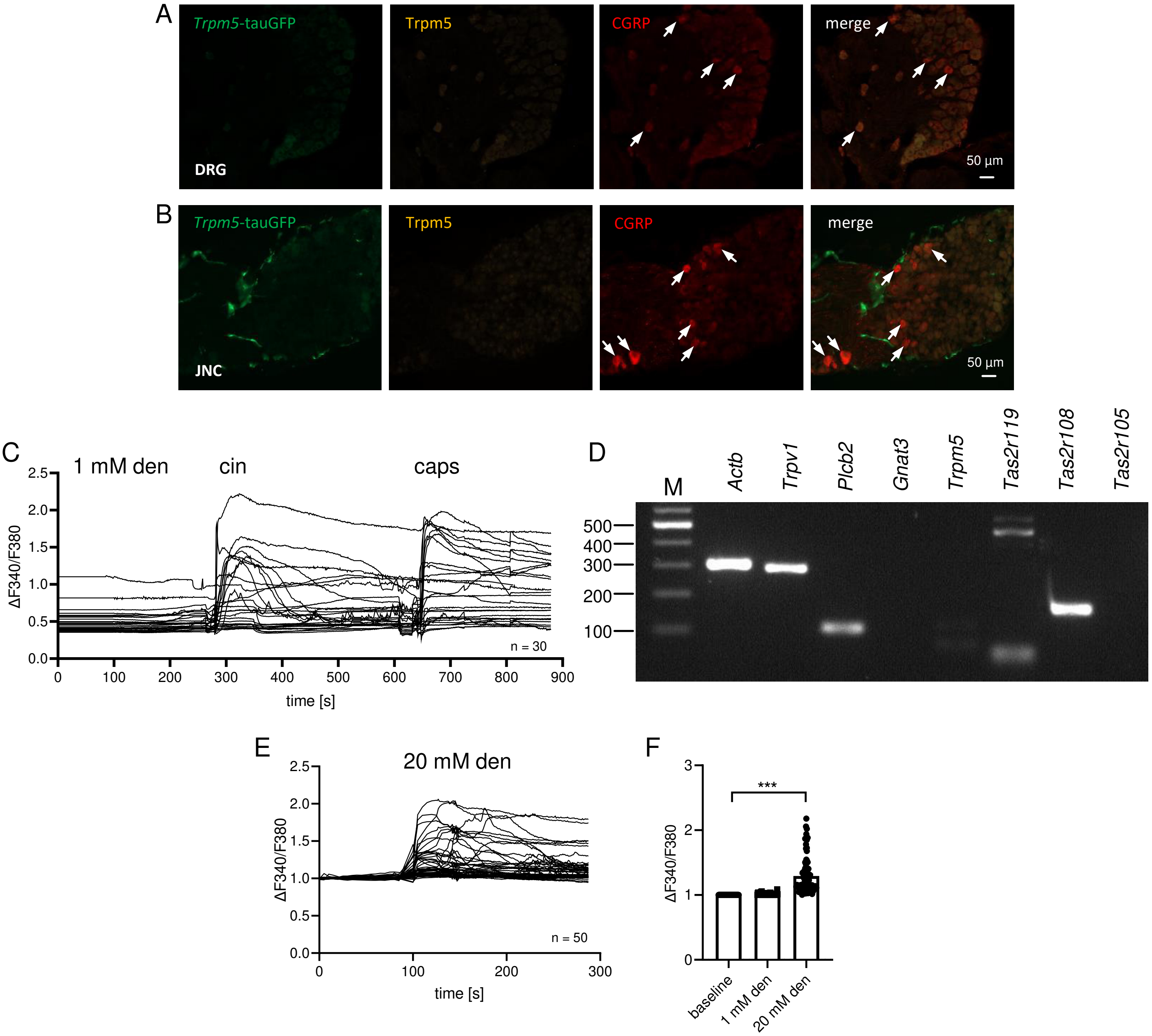

Supplemental Figure 5: Sensory neurons express functional TasR and respond to high concentrations of denatonium. **A)** Representative tissue section of a dorsal root ganglion (DRG) from *Trpm5*-tauGFP mouse (GFP: green) co-stained for Trpm5 (orange) and CGRP (red) showing the lack of Trpm5 expression. Arrows indicate CGRP^{+} neurons. **B)** Representative tissue section of jugular-nodose ganglion (JNC) from *Trpm5*-tauGFP mouse (GFP: green) showing the lack of Trpm5 expression in the neurons co-stained for Trpm5 (orange) and CGRP (red). Arrows indicate CGRP^{+} neurons. Green fluorescence was observed in the perineurium. **C)** Measurements of $[Ca^{2+}]_i$ levels in DRG neurons. DRG neurons do not respond to 1 mM denatonium (den) but to cinnamaldehyde (cin) and capsaicin (caps). **D)** RT-PCR of DRGs revealed expression of the Trpv1, Plcb2, Tas2r119 and Tas2r108 whereas Gnat3, Trpm5 and Tas2r105 were not detected. b-actin (Actb): positive control, M: marker = 100 base pairs. **E)** DRG neurons (32 %) react to 20 mM denatonium (den) with an increase in $[Ca^{2+}]_i$. **F)** Statistical analyses of the response of DRG neurons to 1 mM and 20 mM denatonium (den) compared to baseline. Data are shown as single values and mean ± SEM and were analyzed with one-way ANOVA followed by Bonferroni's multiple comparison. n = 84 neurons from 3 mice.

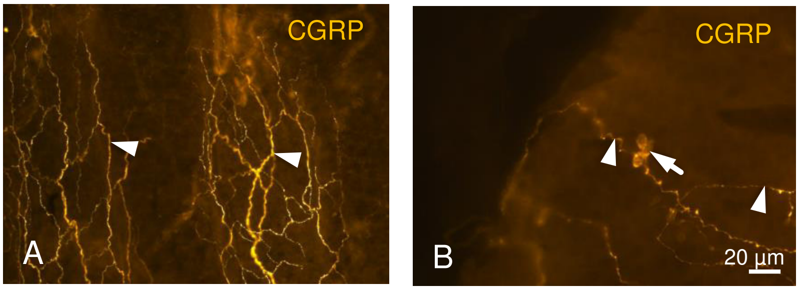

Supplemental Figure 6: Whole mount tracheal preparations. Immunofluorescence. **A)** Whole mount calcitonine gene-related peptide (CGRP) staining of mouse trachea reveals dense innervation of the tracheal mucosa with peptidergic nerve fibers. **B)** Disseminated epithelial cells, most likely neuroendocrine cells, also contain CGRP. **A-B)** Arrowheads show $CGRP^+$ nerve fibers. The arrow shows a $CGRP^+$ cell.

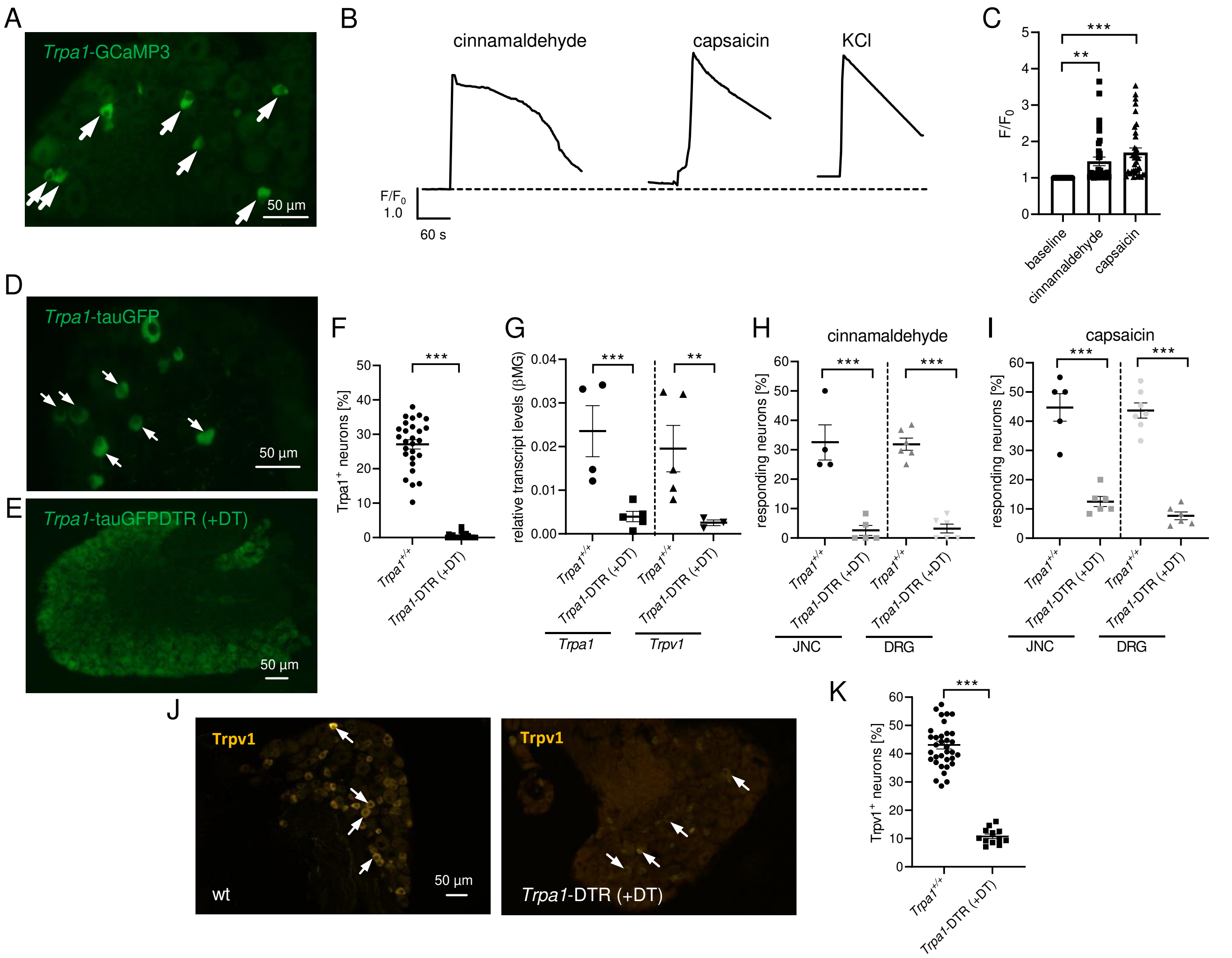

Supplemental Figure 7: Validation of the *Trpa1*-DTR mouse model, dorsal root ganglion (DRG). **A)** Representative tissue section of DRG from a *Trpa1*-GCaMP3 mouse. Arrows show GCaMP3 expressing neurons. **B-C)** *Trpa1*-GCaMP3 expressing neurons react to the Trpa1 agonist cinnamaldehyde (200 µM), the Trpv1 agonist capsaicin (100 nM) and 50 mM KCl with a significant increase in $[Ca^{2+}]_i$. **B)** Representative traces for $[Ca^{2+}]_i$ responses in *Trpa1*-GCaMP3⁺ neurons. **C)** Statistical analysis of responding neurons. **D)** Representative tissue section of a jugular-nodose-complex (JNC) (JNC) from a *Trpa1*-tauGFP mouse. Arrows indicate Trpa1 expressing neurons. **E)** No Trpa1⁺ neurons are observed in a *Trpa1*-DTR mouse model treated with diphtheria toxin (DT), confirming the successful ablation of these neurons by the DT treatment. **F)** Quantitative analyses of Trpa1 expressing neurons in DRG sections from a *Trpa1*$^{+/+}$ and a *Trpa1*-tauGFPDTR mouse model treated with DT. Almost all Trpa1⁺ neurons are ablated after DT treatment in the ganglia of the Trpa1-DTR mouse. n=30 sections **G)** Real-time PCR of Trpa1 and Trpv1 transcripts normalized to β-microglobulin (βMG) in DRGs from *Trpa1*$^{+/+}$ and *Trpa1*-DTR mice treated with DT. n=3-5 mice **H-I)** Calcium imaging experiments with Fura-2 loaded sensory neurons show that the percentage of neurons in JNC and DRG ganglia reacting to cinnamaldehyde (200 µM) or capsaicin (50 nM, dose corresponding to the EC_{50}) is significantly reduced after DT treatment of *Trpa1*-DTR mice compared to *Trpa1*$^{+/+}$ mice. **J)** Representative images of DRG tissue sections from *Trpa1*$^{+/+}$ (wt) and a *Trpa1*-DTR mouse treated with DT stained with a TRPV1 antibody (orange). Arrows indicate TRPV1⁺ neurons. **K)** TRPV1⁺ neurons were reduced from 43% in wt (*Trpa1*$^{+/+}$) mice to approximately 10% in *Trpa1*-DTR mice after treatment with DT. **C, F-I, K)** Data are shown as single values and mean ± SEM and were analyzed with one-way ANOVA followed by Bonferroni's multiple comparison (C) or the unpaired Student's t-test (F-I, K).

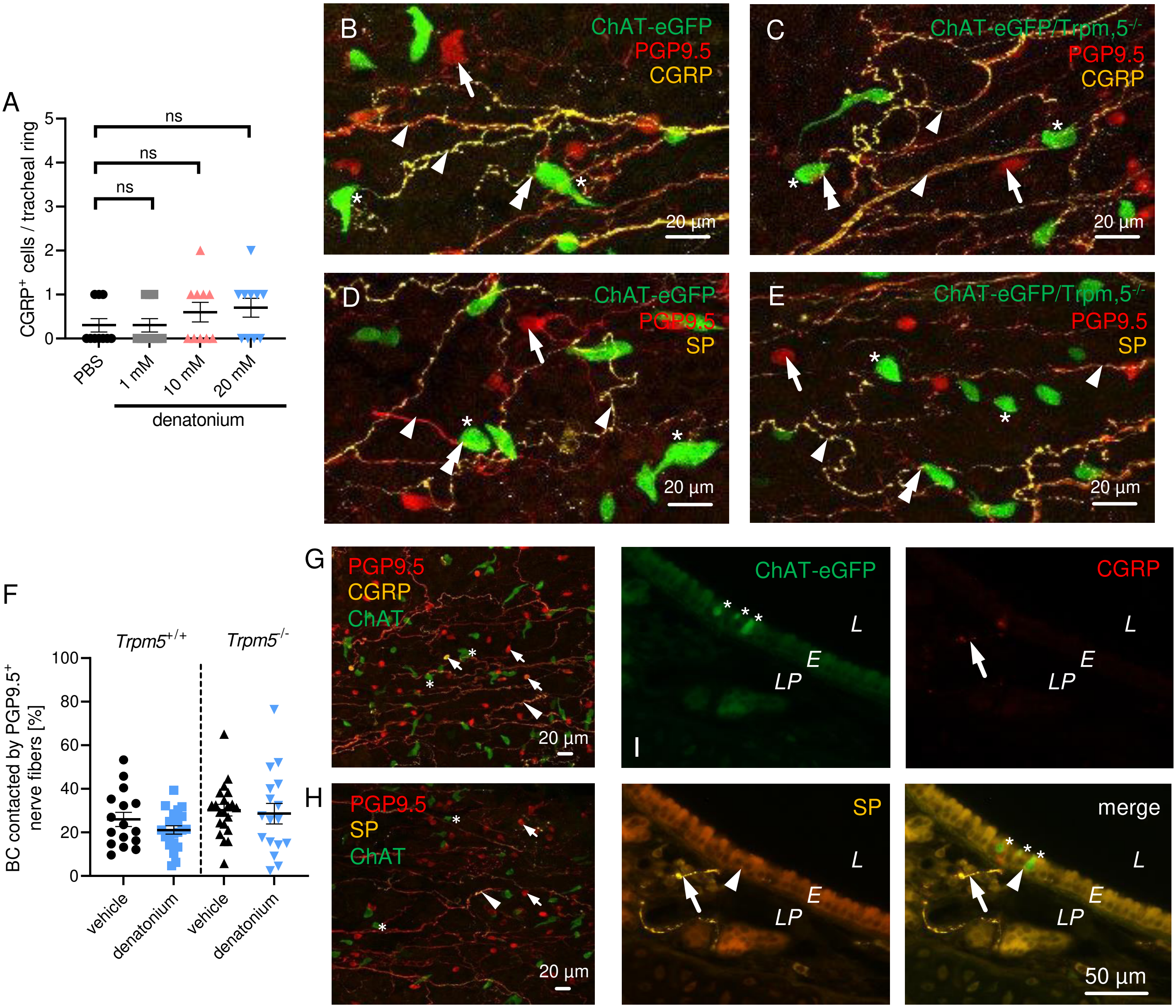

Supplemental Figure 8: Brush cells are approached by SP⁺ sensory nerve fibers. **A)** In tracheal ring sections denatonium (1, 10 or 20 mM) treatment did not have an impact on the number of CGRP⁺ cells (n.s.: not significant, n=10). **B-E)** Double immunostaining of tracheal whole-mounts *Chat*-eGFP and *Chat*-eGFP/*Trpm5*$^{-/-}$ mice show contacts (double arrowheads) of brush cells (green, stars) with CGRP⁺ (yellow, B-C) or SP⁺ (yellow, D-E) and protein gene product 9.5 positive (PGP9.5⁺) nerve fibers (arrowheads). Arrows: PGP9.5⁺ neuroendocrine cells **F)** The contacts between brush cells PGP9.5⁺ nerve fibers are not altered upon stimulation with 1 mM denatonium in *Trpm5*$^{+/+}$ and *Trpm5*$^{-/-}$ mice. n=16-21 acquisitions from 4 mice **G-H)** Double immunostaining of tracheal whole-mount preparations from *Chat*-eGFP mice. GFP-positive brush cells (green, stars) are approached by PGP9.5⁺ (red) and CGRP⁺ or SP⁺ (yellow) nerve fibers (arrowheads). PGP9.5⁺ and CGRP⁺ cells (arrows) are neuroendocrine cells. **I)** Triple immunostaining of a representative tracheal section from *Chat*-eGFP mice. GFP-positive brush cells (stars, green) are approached by CGRP⁺ (red) and Substance P⁺ (SP, yellow) nerve fibers. The arrow indicates a nerve fiber double-positive for SP and CGRP. The arrowhead indicates a contact between a nerve ending (yellow) and brush cell (green). L: lumen, E: epithelium, LP: *lamina propria*. **A, F)** Data are shown as single values and mean ± SEM analyzed with one-way ANOVA followed by Bonferroni's multiple comparisons.

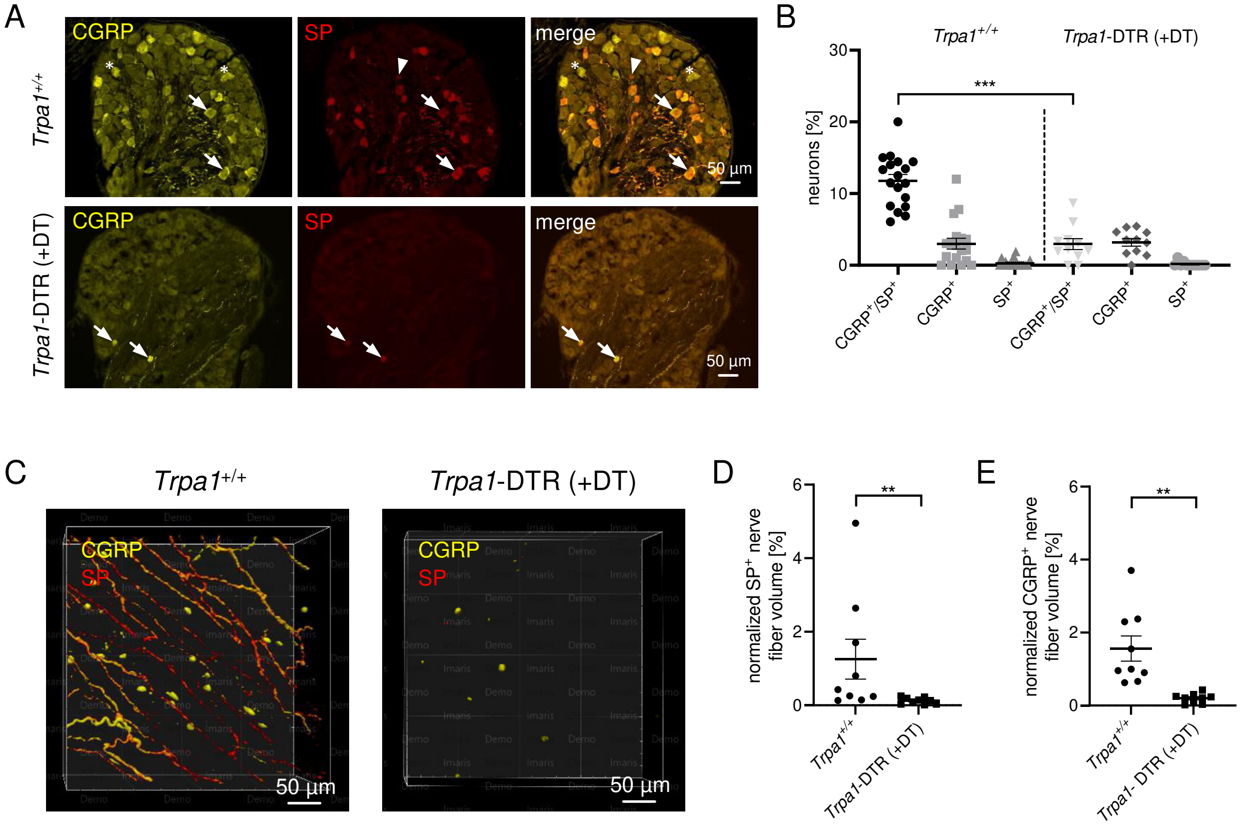

Supplemental Figure 9: Numbers of CGRP⁺ and SP⁺ neurons are reduced upon treatment of *Trpa*1-DTR mice with diphtheria toxin (DT). **A**) Immunostaining of a dorsal root ganglion section (DRG) for CGRP (yellow) and SP (red) in a wild type (*Trpa1*$^{+/+}$, upper panels) and a *Trpa1*-DTR mouse treated with diphtheria toxin (DT, lower panels). CGRP and SP double positive neurons are marked with arrows, CGRP$^+$ with a star (*) and SP$^+$ with an arrowhead. **B**) Quantification of the SP$^+$ and CGRP$^+$ neurons in *Trpa1*$^{+/+}$ mice, revealed that neurons are largely overlapping for both neuropeptides. The amount of double positive neurons (SP$^+$/CGRP$^+$) is significantly reduced in *Trpa1*-DTR mice treated with DT. **C**) Tracheal whole-mount preparations of *Trpa1*$^{+/+}$ and *Trpa1*-DTR mice treated with DT. Immunofluorescence for SP$^+$ (red) and CGRP$^+$ (yellow). 3D-reconstruction of image stacks from the tracheal mucosa with Imaris software. *Trpa1*$^{+/+}$ mice show a dense network of SP$^+$ (red) and CGRP$^+$ (yellow) nerve fibers (left panel) whereas peptidergic nerve fibers are almost absent in tracheas of *Trpa1*-DTR mice treated with DT. **D-E**) The SP$^+$ (**D**) and CGRP$^+$ (**E**) nerve fiber volume (normalized to a total volume of $1*10^7$) is significantly reduced in tracheal whole mounts of *Trpa1*-DTR mice treated with DT compared to *Trpa1*$^{+/+}$ mice. **B, D-E)** Data are presented as single values and mean ± SEM and were analyzed with one-way ANOVA followed by Bonferroni's multiple comparisons (B) or the unpaired Student's t-test (D-E).

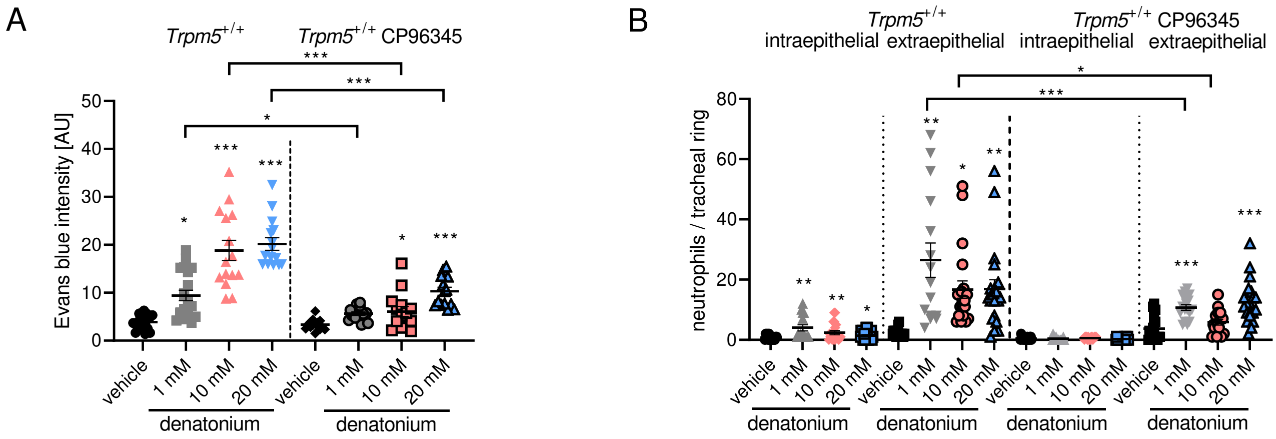

Supplemental Figure 10: Denatonium-induced neurogenic inflammation is reduced by the NK1 receptor inhibitor CP96345. **A)** Comparison of data from Figure 2E and 6C in the main article. Evans blue intensity in response to 1 mM, 10 mM and 20 mM denatonium was significantly reduced in Trpm5$^{+/+}$ mice treated with CP96345 compared to Trpm5$^{+/+}$ mice without CP96345. **B)** Comparison of data from Figure 3G and 6D in the main article. Extraepithelial neutrophil numbers in response to 1 mM and 10 mM denatonium were significantly upon treatment of Trpm5$^{+/+}$ mice with CP96345. **A-B)** Data are shown as single values and mean ± SEM and were analyzed one-way ANOVA followed by Bonferroni's multiple comparisons. *p<0.05, **p<0.01, ***p<0.001

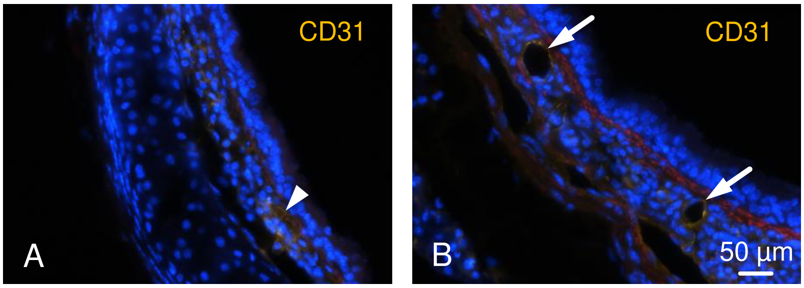

Supplemental Figure 11: Representative images from tracheal sections of *SP*$^{-/-}$ mice labeled for CD31 (blood vessels, arrowheads) and nuclei counterstain (DAPI, blue). **A)** The lumen of blood vessels in the *lamina propria* in vehicle-treated *SP*$^{-/-}$ mice was not changed (arrowheads). **B)** In contrast, dilated blood vessels with apparent lumen were observed in tracheal section of a *SP*$^{-/-}$ mice treated with 20 mM denatonium (arrows).

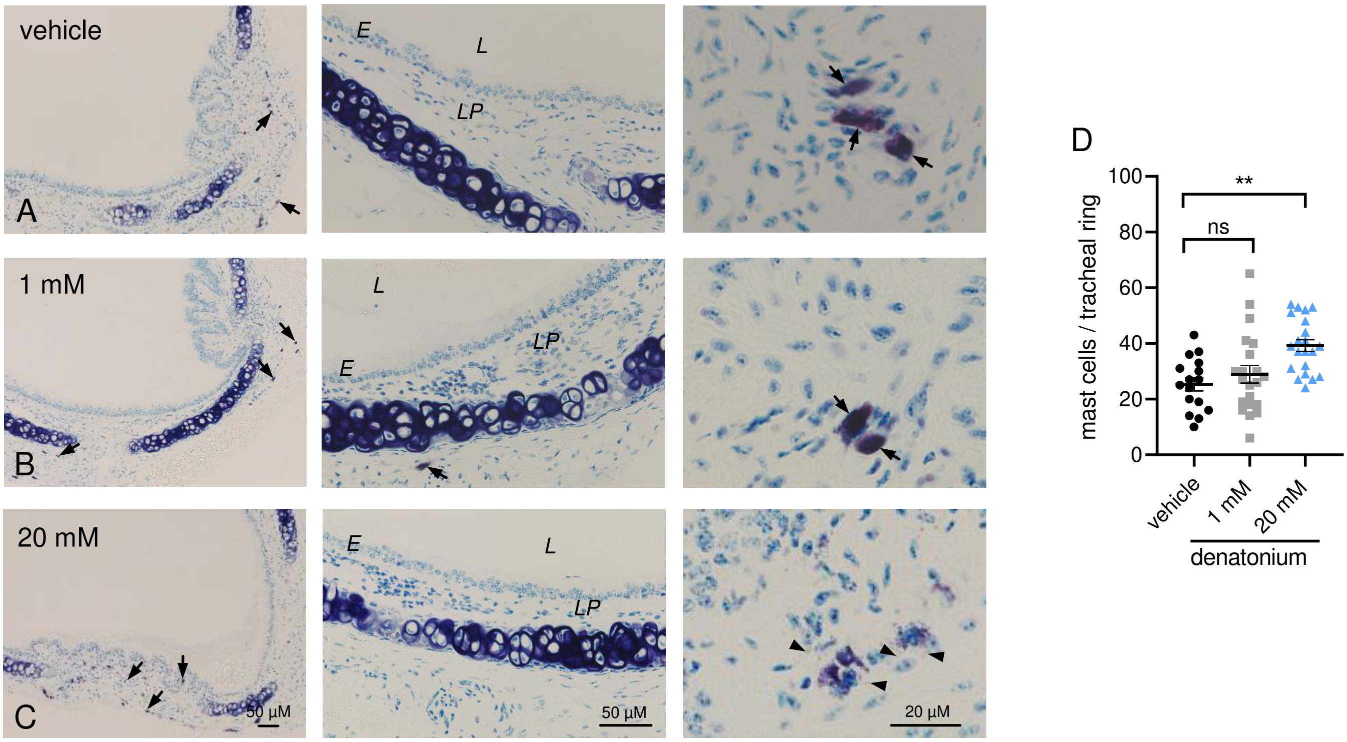

Supplemental Figure 12: The role of mast cells in denatonium-induced neurogenic inflammation. (**A-C**) Toluidine blue staining of tracheal tissue sections from wild-type mice that inhaled vehicle, 1 or 20 mM denatonium. Mast cells are found in the trachea only in the *paries* membranaceus. They are are rarely in the *lamina propria* (LP) or in close proximity to the epithelium (E). Arrows indicate mast cells. Arrowheads indicate granules from degranulated mast cells. L: lumen. (**D**) In tracheae treated with 1 mM denatonium the mast cell numbers are unchanged compared to vehicle controls, whereas they are significantly increased in tracheae treated with 20 mM denatonium. Data are depicted as mean ± SEM, analyzed with one-way ANOVA followed by Bonferroni's multiple comparison. **p<0.01, n=16-21 rings from 3 mice

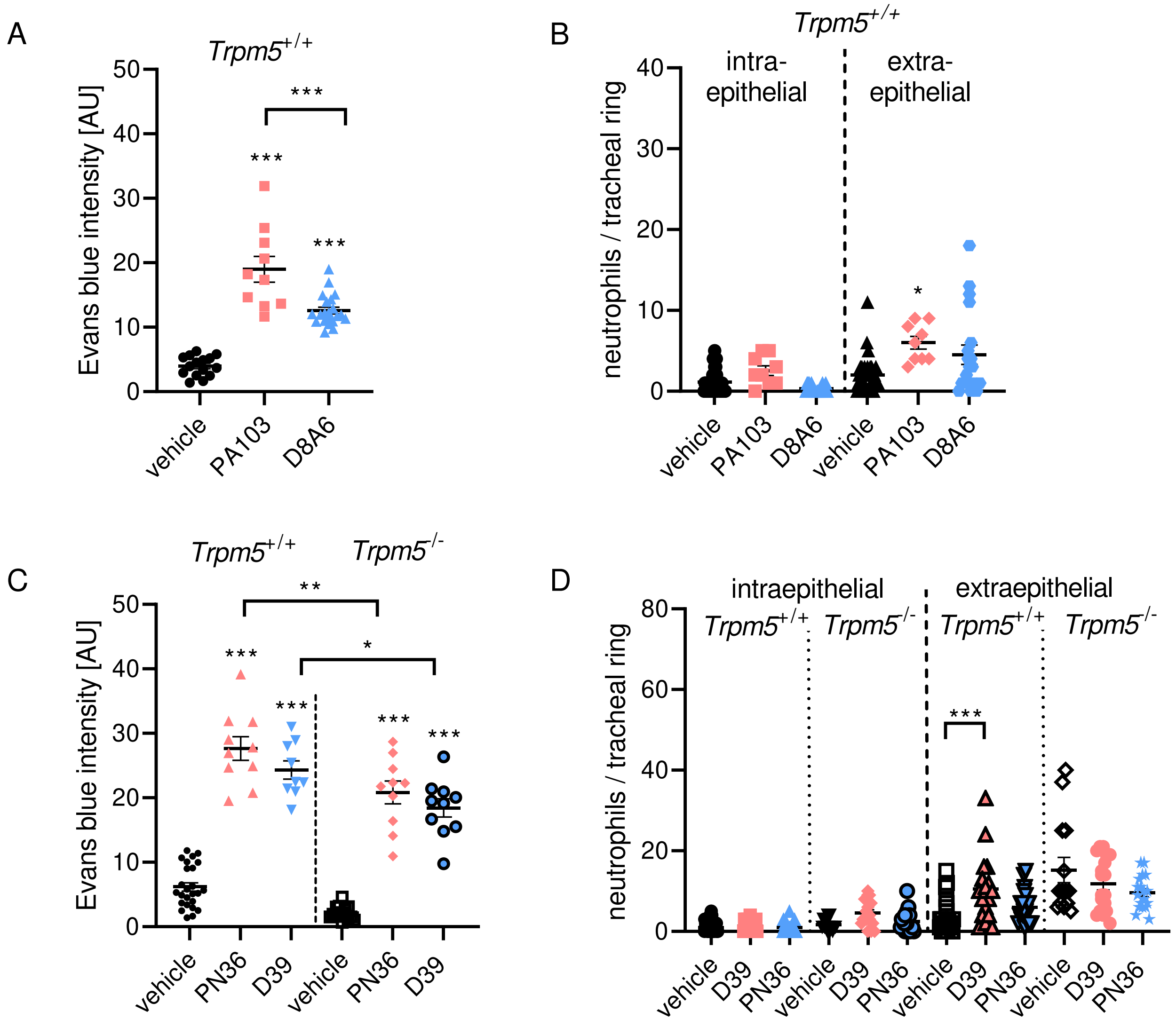

Supplemental Figure 13: Evans blue extravasation and neutrophil recruitment in response to bacterial bacterial culture supernatants. **A)** Application of the *Pseudomonas aeruginosa* Pa103 supernatant increased Evans blue intensity significantly in wild-type mice. Supernatants of the *Pseudomonas aeruginosa* strain D8A6, a quorum-sensing molecule-deficient strain, significantly increased Evans blue intensity, but Evans blue intensity was significantly less than in the Pa103 strain. **B)** Pa103 and D8A6 supernatants did not influence intra- or extraepithelial neutrophil numbers in wild-type mice. **C)** Application of supernatants of cultures of two *Streptococcus pneumoniae* strains (D39, PN36) significantly increased Evans blue intensity in wild-type and *Trpm5*$^{-/-}$ mice. The Evans blue intensity induced by PN36 and D39 was significantly reduced in *Trpm5*$^{-/-}$ mice compared to wild-type controls. The vehicle controls were not significantly different from each other. **D)** D39 and PN36 supernatants did not influence intra-epithelial neutrophil numbers in wild-type or *Trpm5*$^{-/-}$ mice. D39 supernatants increased extraepithelial neutrophils in wild-type mice, but not in *Trpm5*$^{-/-}$ mice. Supernatants of PN36 did not influence extraepithelial neutrophil numbers in wild-type or *Trpm5*$^{-/-}$ mice. **A-D)** Data are presented as single values and mean ± SEM and were analyzed with one-way ANOVA followed by Bonferroni's multiple comparisons. *: p<0.05, **: p<0.01, ***: p<0.001

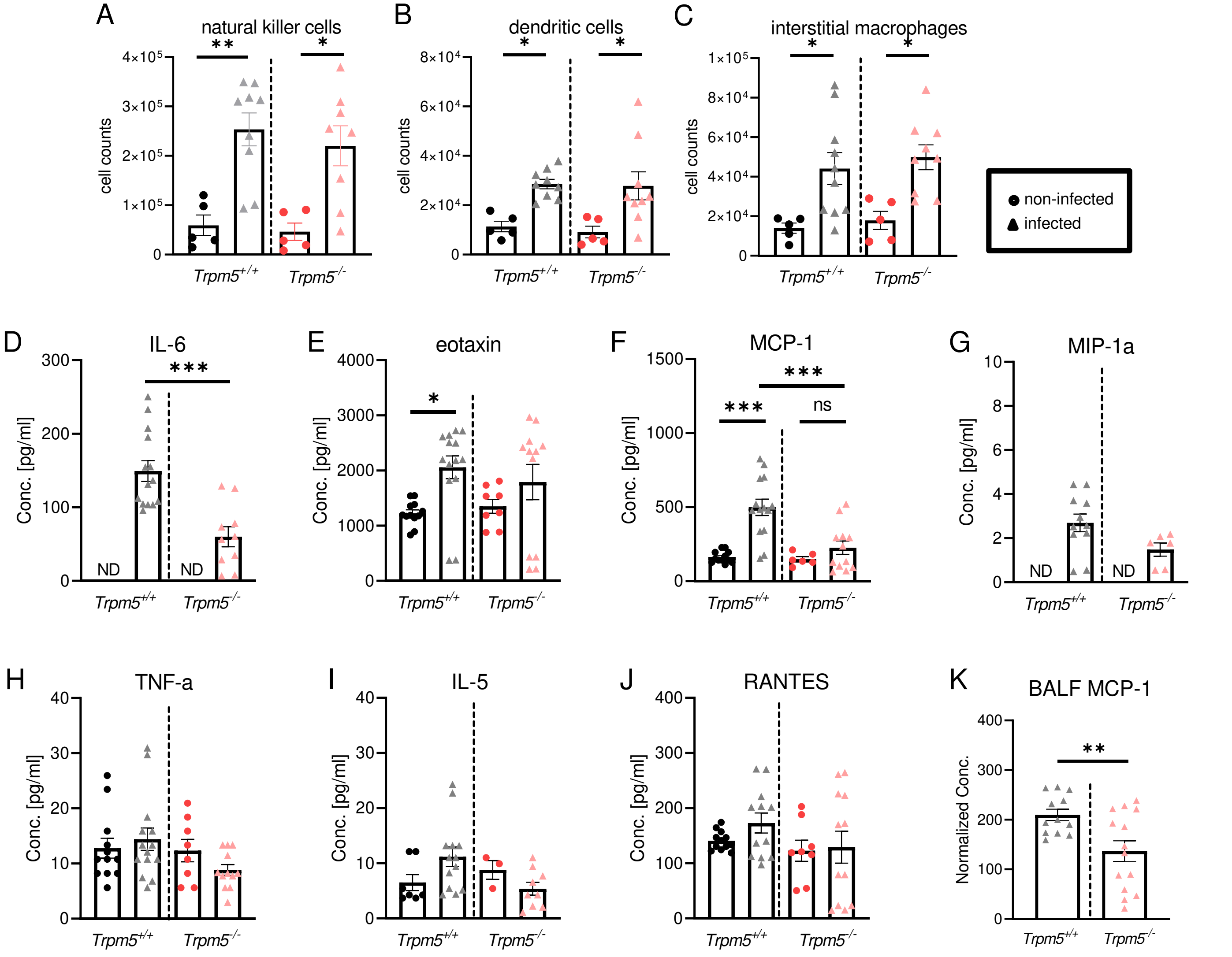

Supplemental Figure 14: Immune cell and cytokine profiles of *Trpm5*$^{+/+}$ and *Trpm5*$^{-/-}$ mice before and after infection. Circles represent values from non-infected samples and triangles from infected. **A-C)** FACS analysis of immune cells in lungs 4 h post infection and in healthy control animals. The number of natural killer cells, dendritic cells and interstitial macrophages increased upon infection in *Trpm5*$^{+/+}$ and *Trpm5*$^{-/-}$ mice. **D-J)** Cytokine measurements of blood samples of *Trpm5*$^{+/+}$ and *Trpm5*$^{-/-}$ mice before and after 4 h of infection. **D)** The increase in IL-6 concentration was more prominent in *Trpm5*$^{+/+}$ mice than in *Trpm5*$^{-/-}$ mice after infection. (ND: not detectable, ***: $p<0.001$) **E)** The eotaxin concentration was significantly increased in *Trpm5*$^{+/+}$ mice after infection. (*: $p<0.05$) **F)** MCP-1 levels were significantly increased in *Trpm5*$^{+/+}$ mice but not in *Trpm5*$^{-/-}$ mice. (***: $p<0.001$, ns: not significant) **G)** MIP-1a was not detectable before infection and after infection increased to a similar extend in *Trpm5*$^{+/+}$ and *Trpm5*$^{-/-}$ mice. **H-J)** No significant changes in TNF-α, IL-5 and RANTES concentrations were observed. **K)** Cytokine measurements of bronchoalveolar lavage fluid (BALF) samples of *Trpm5*$^{+/+}$ and Trpm5$^{-/-}$ mice 4 h post infection. MCP-1 levels were higher in *Trpm5*$^{+/+}$ mice than in *Trpm5*$^{-/-}$ mice. (**: $p<0.01$). **A-K)** Data are presented as single values and mean ± SEM and were analyzed with one-way ANOVA followed by Bonferroni's multiple comparisons. *: $p<0.05$, **: $p<0.01$, ***: $p<0.001$

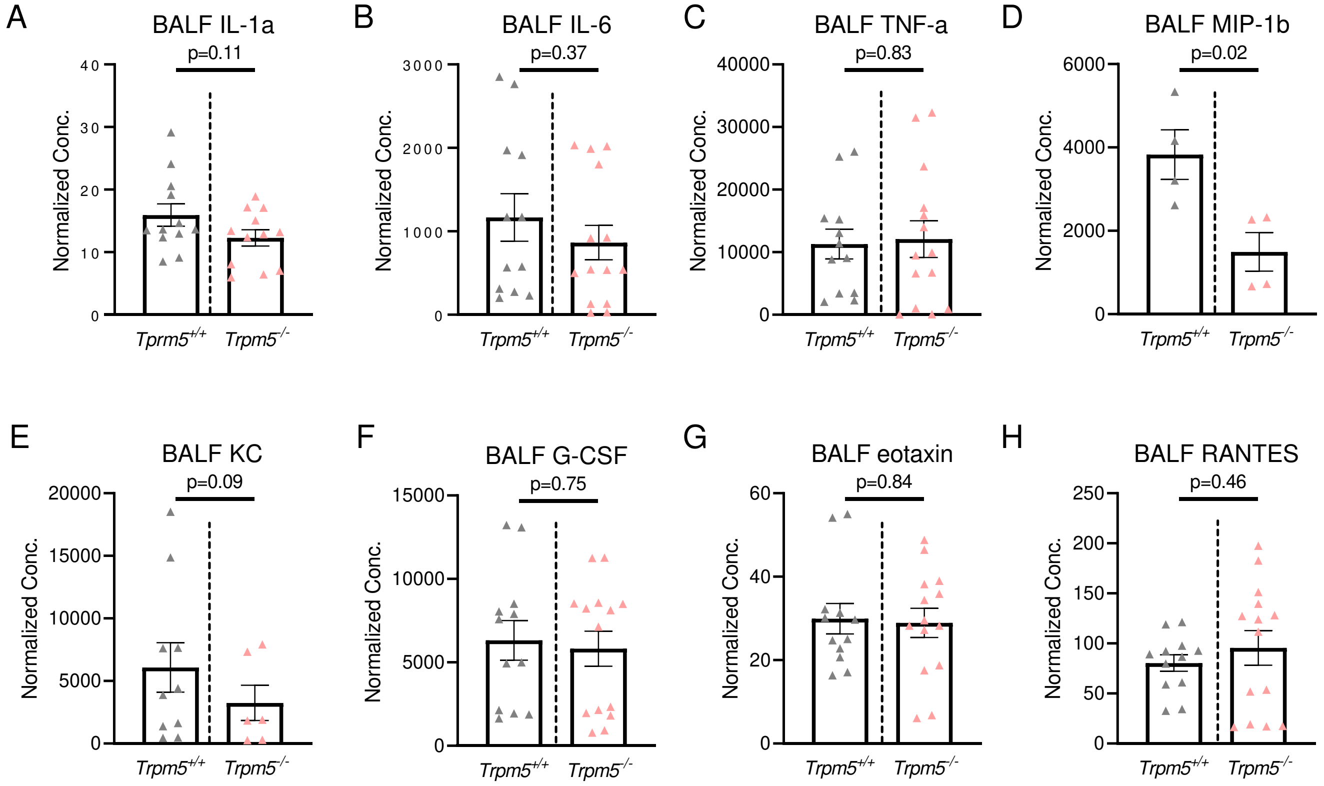

Supplemental Figure 15: Cytokine profile of *Trpm5*$^{+/+}$ and *Trpm5*$^{-/-}$ mice in bronchoalveolar lavage fluid (BALF) 4 h post infection. **A)** Measurements of IL-1α which generally promotes inflammation and can be released by neutrophils, macrophages, epithelial and endothelial cells trended towards lower levels in *Trpm5*$^{-/-}$ than in *Trpm5*$^{+/+}$ mice. **B-C)** Measurements of IL-6 and TNFα which both can be released by macrophages were not different in *Trpm5*$^{+/+}$ and *Trpm5*$^{-/-}$ mice. **D)** MIP-1b, the chemoattractant for natural killer cells and monocytes, was significantly lower in *Trpm5*$^{-/-}$ than in *Trpm5*$^{+/+}$ mice. Most samples were out of range and therefore were not included with the values for *Trpm5*$^{+/+}$ mice being too high for reliable measurement and the values of the *Trpm5*$^{-/-}$ mice too low. **E)** KC, the neutrophil chemoattractant, trended to show lower concentrations in *Trpm5*$^{-/-}$ compared to *Trpm5*$^{+/+}$ mice. Values that were out of range were excluded. **F)** The concentration of G-CSF, a cytokine involved in survival, proliferation, differentiation and function of neutrophils, was similar in *Trpm5*$^{+/+}$ and *Trpm5*$^{-/-}$ mice. **G-H)** The concentrations of the cytokines eotaxin and RANTES, both chemotactic for T lymphocytes and eosinophils, were similar in *Trpm5*$^{+/+}$ and *Trpm5*$^{-/-}$ mice. **A-H)** Data are presented as single values and mean ± SEM and were analyzed with one-way ANOVA followed by Bonferroni's multiple comparisons. n=4-14 samples *: $p<0.05$, **: $p<0.01$, ***: $p<0.001$

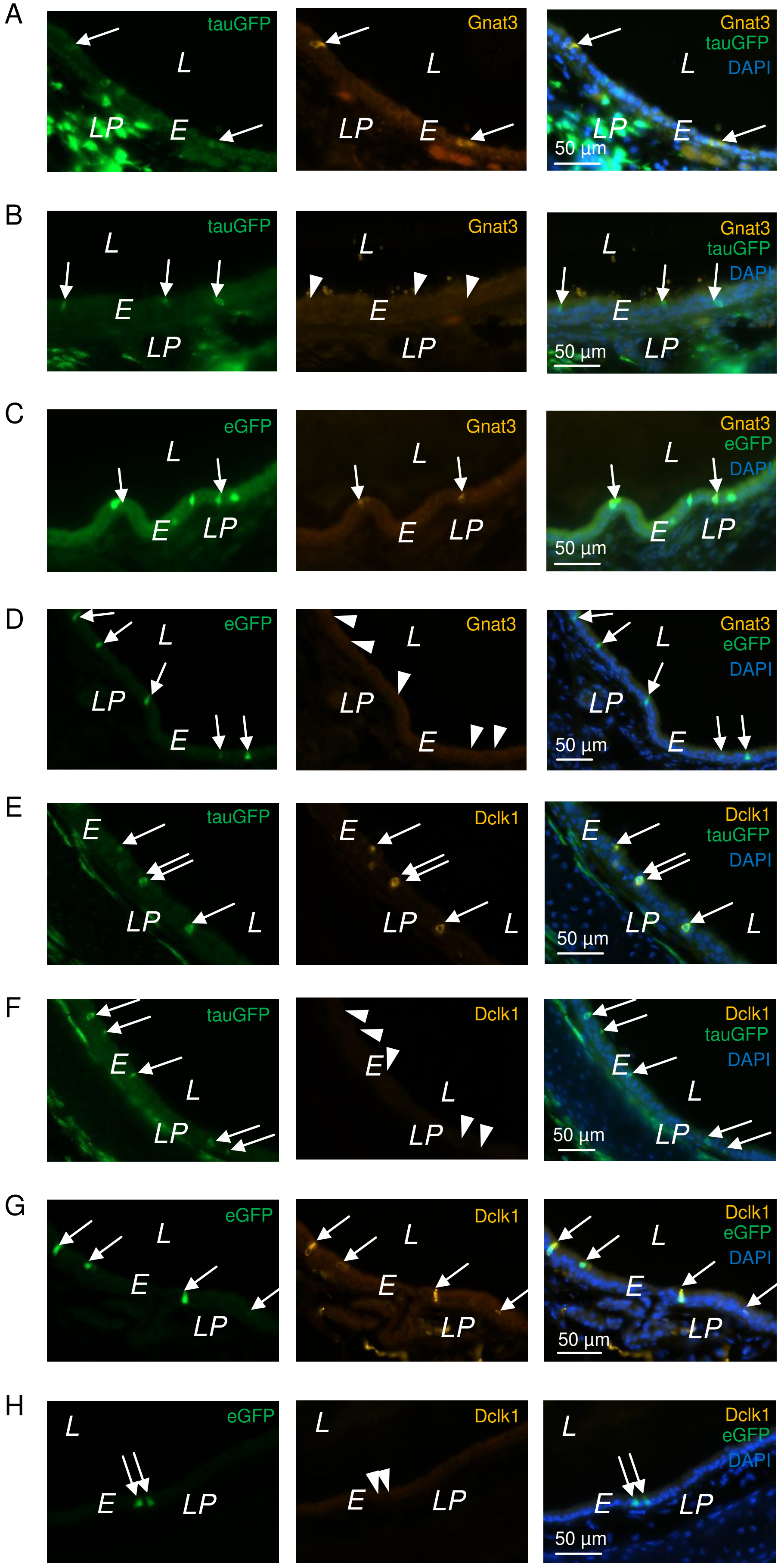

Supplemental Figure 16: Pre-absorption controls for the specificity of the antibodies. Immunohistochemistry, trachea, *Trpm5*tauGFP and *ChAT*-eGFP mice. **A, C)** *Trpm5*-tauGFP⁺ and ChAT-eGFP⁺ BC (arrows, green) exhibit Gnat3 staining (orange). **B, D)** No Gnat3 staining is observed in tauGFP⁺ and eGFP⁺ cells after pre-absorption of the antibody with the corresponding peptide. **E, G)** Immunostaining of *Trpm5*-tauGFP⁺ and ChAT-eGFP⁺ BC (arrows, green) for Dclk1 (orange). Dclk1 (orange) colocalizes with tauGFP and eGFP. **F, H)** Pre-absorption of the Dclk1 antibody. No Dclk1 staining is detectable in BC in both mouse strains. **A-H)** L: lumen, E: epithelium, LP: *lamina propria*.

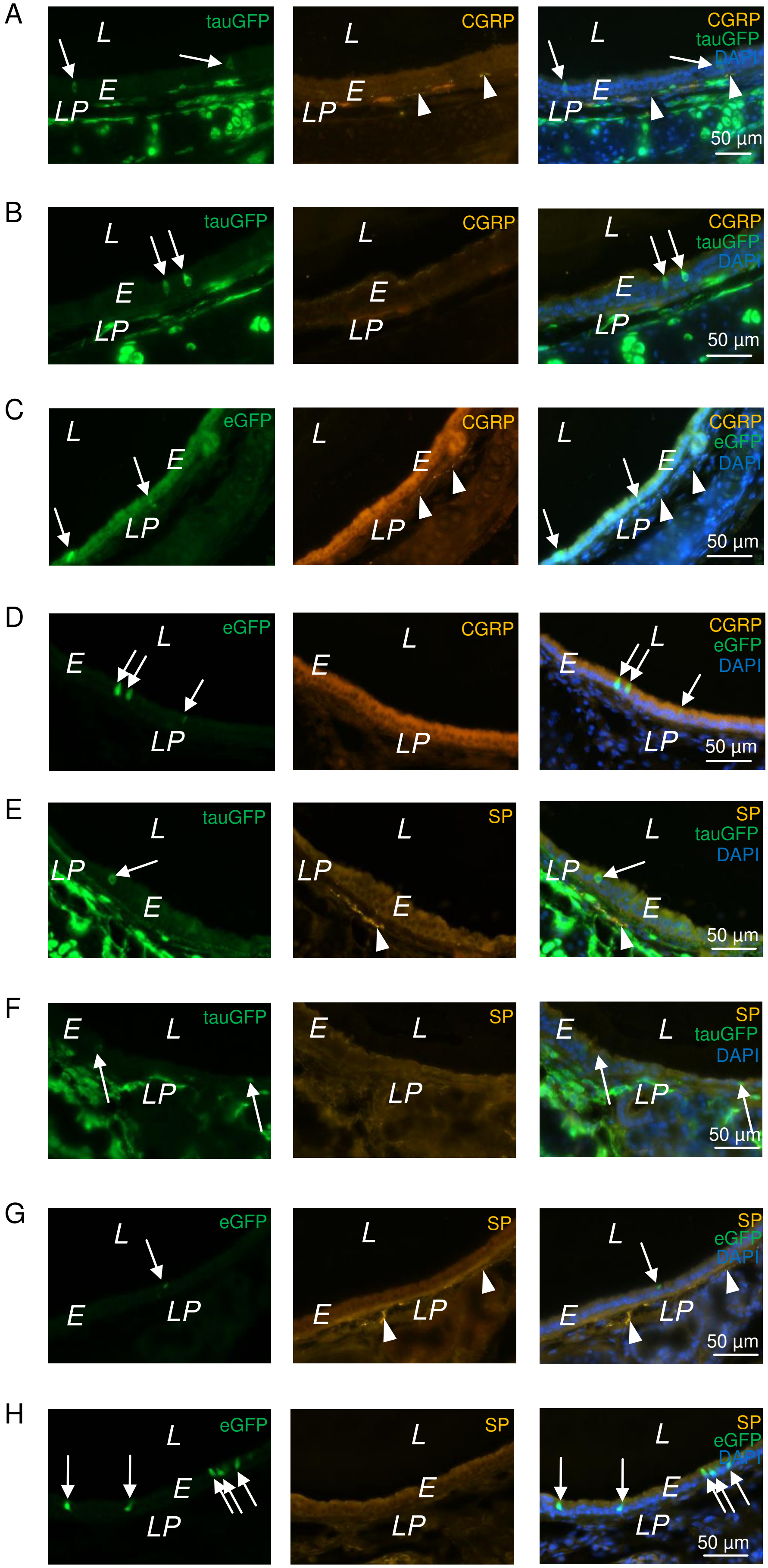

Supplemental Figure 17: Pre-absorption controls for the specificity of the antibodies. Immunohistochemistry, trachea, *Trpm5*-tauGFP and *ChAT*-eGFP mice. **A, C)** CGRP⁺ nerve fibers (orange, arrowheads) are present in tracheal slices of *Trpm5*-tauGFP and *ChAT*-eGFP mice (*Trpm5*-tauGFP⁺ and ChAT-eGFP⁺ BC: arrows, green) **B, D)** No CGRP staining is observed in both mouse strains after pre-absorption of the antibody with the corresponding peptide. **E, G)** SP⁺ nerve fibers (orange, arrowheads) are present in tracheal slices of *Trpm5*-tauGFP and ChAT-eGFP mice (*Trpm5*-tauGFP⁺ and ChAT-eGFP⁺ BC: arrows, green). **F, H)** No SP staining is observed in either mouse strains after pre-absorption of the antibody with the corresponding peptide. **A-H)** L: lumen, E: epithelium, LP: *lamina propria*.

Supplementary Table 3: Table of primary and secondary antibodies used for immunohistochemistry and FACS

**Antigene**	**Immunogen and/or Conjugate**	**Species/clone**	**Concentration**	**Catalog no.**	**Source**
DCAMKL1	synthetic peptide corresponding to mouse DCAMKL1 aa 700 to the C-terminus (C terminal) conjugated to keyhole limpet haemocyanin.	rabbit / polyclonal	1:1600	ab31704<br>Lot no. GR310497-1	abcam
GNAT3	synthetic peptide C-KNQFLDLNLKKEDKE from an internal region of human GNAT3	goat / polyclonal	1:400	pab73402<br>Batch 65974	Covalab
TRPM5	C-terminal fragment of mouse TRPM5 (RefSeq NP_064673.2)	rabbit	1:800	generated in-house	VF
TRPM5	peptides coupled via the N-terminus to keyhole limpet hemocyanin described in Kaske et al. 2007	rabbit / polyclonal	2 µg/ml	generated in-house	VC, TG
CD31	murine amino acid fragment (amino acids 610-681 of mouse CD31)	rat / monoclonal	1:400	DIA-310<br>Lot 15916/05	dianova
CGRP	KLH-conjugated synthetic peptide encompassing a sequence within the C-term region of mouse CGRP	rabbit / polyclonal	1:1000	250602<br>Lot no. 17010909	Abbiotec
SP	epitope mapping near the C-terminus	rat / monoclonal	1:400	SC-21715<br>Lot no. B1012	Santa Cruz
Complement C3	C57BL76 thymocytes saturated with rat anti-Thy-1 monoclonal antibody of IgG2b subclass (RmT1)	rat / monoclonal	1:400	NB200-540<br>Lot no. AB042114a-09	Novus Biologicals
Pseudomonas aeruginosa	whole formaldehyde killed bacteria, *Pseudomonas aeruginosa* strains PAO1 and Habs1	chicken / polyclonal	1:800	ab74980<br>Lot no. GR3236883-5	abcam
Ly6G-FITC	not specified, FITC	rat / monoclonal	1:200	RM3001<br>Lot 1647833	Life technologies
PGP9.5	Recombinant full-length human PGP9.5 protein	rabbit / polyclonal	1:200	ab15503<br>Lot no. GR256354-1	abcam

TRPV1	Peptide (C)EDAEVFKDSMVPGEK, corresponding to amino acid residues 824-838 of rat TRPV1	rabbit / polyclonal	1:2000	ACC-030<br>Lot no. ACC030AN3525	alomone labs
donkey anti-chicken Cy3	Cyanine Cy3	donkey	1:1500	703-165-155<br>Lot no. 136499	Jackson ImmunoResearch
donkey anti-goat Cy3	Cyanine Cy3	donkey	1:500	AP180C<br>Lot 2707848	Merck Millipore
donkey anti-rat Cy3	Cyanine Cy3	donkey	1:1000	712-165-150<br>Lot no. 123375	Jackson ImmunoResearch
donkey anti-rabbit Cy3	Cyanine Cy3	donkey	1:1000	AP182C<br>Lot 2548999	Merck Millipore
donkey anti-rabbit Cy5	Cyanine Cy5	donkey	1:500	711-175-152<br>Lot no. 122922	Jackson ImmunoResearch
donkey anti-rat Cy5	Cyanine Cy5	donkey	1:400	712-175-153<br>Lot no. 144475	Jackson ImmunoResearch
CD11c Super Bright 436	Super Bright 436	armenian hamster / monoclonal	1:40	62-0114-82<br>Lot 1967081	eBioscience
CD11b FITC	FITC	rat / monoclonal	1:40	11-0112-82<br>Lot 1989138	eBioscience
Ly6G APC	APC	rat / monoclonal	1:40	17-9668-82<br>Lot 2055824	eBioscience
CD45 PerCP Cy5.5	PerCP-Cyanine Cy5.5	rat / monoclonal	1:150	45-0451-82<br>Lot 1994158	eBioscience
F4/80 PECy7	PE-Cyanine Cy7	rat / monoclonal	1:40	25-4801-82<br>Lot 2066766	eBioscience
CD86 PE	PE	rat / monoclonal	1:133	12-0862-82<br>Lot 2045467	eBioscience

Supplementary Table 4: Oligonucleotide primers for PCR analysis

Gene	Genebank accession No.	Primer	Product length (bp)
B2m	NM_009735.3	fwd accctggtctttctggtgct<br>rev aatgtgaggcgggtggaa	150
Actb	NM_007393.5	fwd gtgggaatgggtcagaagg<br>rev ggcatacagggacagcaca	300
Gnat3	NM_001081143.1	fwd tcatccataagaatggttacagc<br>rev cccacagtcgtttaatgatttc	231
Plcb2	NM_177568.2	fwd ttccagatgtttcctgctga<br>rev gggaagtcctctgggttgat	101
Tas2r105	NM_020501.1	fwd gactggcttccttctcatcg<br>rev gcaaacaccccaagagaaaa	284
Tas2r108	NM_020502.1	fwd tggatgcaaacagtctctgg<br>rev ggtgagggctgaaatcagaa	158
Tas2r119	NM_020503.3	fwd cgatgctctccattctgtca<br>rev tgatgagtagcaggcactgg	289
Trpa1	NM_001348288.1	fwd gtccagggcgttgtctatcg<br>rev cgtgatgcagaggacagagat	163
Trpm5	NM_020277.2	fwd tgaggaacgacctttggcta<br>rev acacggatcttggtggatgt	183
Trpv1	NM_001001445.2	fwd tcaccgtcagctctgttgtc<br>rev gggtctttgaactcgctgtc	285
